# Multiplexed smFISH Reveals the Spatial Organization of Neuropil Localized mRNAs Is Linked to Abundance

**DOI:** 10.1523/ENEURO.0184-25.2025

**Published:** 2025-12-09

**Authors:** Renesa Tarannum, Grace Mun, Fatima Quddos, Sharon A. Swanger, Oswald Steward, Shannon Farris

**Affiliations:** ^1^Fralin Biomedical Research Institute at Virginia Tech Carilion, Center for Neurobiology Research, Roanoke, Virginia 24016; ^2^Translational Biology, Medicine & Health Graduate Program, Virginia Tech, Blacksburg, Virginia 24061; ^3^Department of Biomedical Sciences & Pathobiology, Virginia-Maryland College of Veterinary Medicine, Virginia Tech, Blacksburg, Virginia 24061; ^4^Virginia Tech Carilion School of Medicine, Roanoke, Virginia 24016; ^5^Department of Anatomy & Neurobiology, University of California Irvine School of Medicine, Irvine, California 92697

**Keywords:** mRNA localization, multiplex imaging, neurons, RNA granule, RNP composition

## Abstract

RNA localization to neuronal axons and dendrites provides spatiotemporal control over gene expression to support synapse function. Neuronal messenger RNAs (mRNAs) localize as ribonucleoprotein particles (RNPs), commonly known as RNA granules, the composition of which influences when and where proteins are made. High-throughput sequencing has revealed thousands of mRNAs that localize to the hippocampal neuropil. Whether these mRNAs are spatially organized into common RNA granules or distributed as independent mRNAs for proper delivery to synapses is debated. Here, using highly multiplexed single-molecule fluorescence in situ hybridization (HiPlex smFISH) and colocalization analyses, we investigate the subcellular spatial distribution of 15 synaptic neuropil localized mRNAs in the male and female rodent hippocampus. We observed that these mRNAs are present in the neuropil as heterogeneously sized fluorescent puncta with spatial colocalization patterns that generally scale by neuropil mRNA abundance. Indeed, differentially expressed mRNAs across cell types displayed colocalization patterns that scaled by abundance, as did simulations that reproduce cell-specific differences in abundance. Thus, the probability of these mRNAs colocalizing in the neuropil is best explained by stochastic interactions based on abundance, which places constraints on the mechanisms mediating efficient transport to synapses.

## Significance Statement

RNA localization establishes compartment-specific gene expression that is critical for synapse function. Thousands of mRNAs localize to the hippocampal synaptic neuropil; however, whether mRNAs are spatially organized as similar or distinctly composed ribonucleoprotein particles for delivery to synapses is unknown. Using multiplexed smFISH to assess the spatial organization of 15 neuropil localized mRNAs, we find that these mRNAs are present in variably sized puncta suggestive of heterogeneous transcript copy number states. RNA colocalization analyses in multiple hippocampal cell types suggest that the spatial relationship of these mRNAs is best described by their abundance in the neuropil. Stochastic RNA–RNA interactions based on neuropil abundance are consistent with models indicating that global principles, such as energy minimization, influence population localization strategies.

## Introduction

Neuronal morphology is incredibly complex, and in order for neurons to function efficiently, messenger RNA (mRNA) transcripts need to be delivered to distant sites for on-demand translation. In particular, mRNA localization to synapses and subsequent local translation are required for the synaptic plasticity underlying learning (Huber et al., 2000; Bradshaw et al., 2003). Dysregulation of these processes is a common cause of intellectual disability and autism (Huber et al., 2002; Fernandopulle et al., 2021). Thus, uncovering how mRNA cargoes are delivered to and locally regulated at the synapse is central to our understanding of the molecular basis of learning and memory. Furthermore, studying the fundamental principles of neuronal mRNA localization can uncover key aspects of post-transcriptional regulation, which could be applicable to various other organisms and cell types, such as yeasts, drosophila germ cells, cardiomyocytes, etc., that use compartmentalization for gene regulation (Martin and Ephrussi, 2009; Lewis et al., 2018).

The hippocampus is a brain region critical for learning and memory that has a laminar organization ideal for cataloging and visualizing localized mRNAs in the axon- and dendrite-rich neuropil layer. Compartment-specific deep sequencing studies have revealed the presence of thousands of mRNA species localized in the rodent hippocampal synaptic neuropil (Cajigas et al., 2012; Farris et al., 2019). These localized mRNAs are assembled and transported throughout the neuropil as “ribonucleoprotein particles” (RNPs, otherwise known as RNA granules), which are dynamic, spherical, membraneless macromolecular complexes of nontranslating mRNAs, mRNA-binding proteins (RBPs), and translational machinery (Knowles et al., 1996; Kiebler and Bassell, 2006). Several flavors of RNPs localize to the synaptic neuropil, including transport RNPs, RISC RNPs, translating RNPs, p-bodies, and stress granules (Bauer et al., 2023; Kiebler and Bauer, 2024). These RNPs are typically characterized by their RBP constituents [e.g., FMRP (Antar et al., 2005), DCP1, AGO2 (Cougot et al., 2008; Zeitelhofer et al., 2008), CPEB (Huang et al., 2003), EIF4E (Napoli et al., 2008), G3BP (Sahoo et al., 2018), staufen (Sharangdhar et al., 2017)] despite significant heterogeneity in composition due to the overlap and exchange of RBPs between RNP types. Recent in vitro studies in neurons and human cell lines show that mRNA is a key driver of mRNA–protein condensates by influencing RNP composition and size through seeding of higher order molecular assemblies (Maharana et al., 2018; Garcia-Jove Navarro et al., 2019; Bauer et al., 2022). However, investigations into the mRNA composition of RNPs and whether it contributes to molecular specificity have traditionally been overlooked.

Several different models have been proposed to explain how mRNAs are assembled into RNPs to dictate their destination. One hypothesis is that mRNAs are transported as single or low copy number molecules per RNP. Single-molecule fluorescence in situ hybridization (smFISH) studies in cultured neurons revealed that individual dendritic RNAs, whether in transport or localized, carry no more than one or two molecules of a specific type of transcript (Mikl et al., 2011; Batish et al., 2012). Similar studies also revealed whether specific pairs of dendritically localized mRNAs coexist in common particles (Gao et al., 2008) or not (Tübing et al., 2010; Mikl et al., 2011; Batish et al., 2012). However, larger-scale observations were precluded due to limitations in multicolor RNA labeling. Thus, there is limited evidence on the heterogeneity (how much of a given mRNA) and diversity (how many types of different mRNAs) of neuronal RNP compositions as well as their spatial distribution in intact neural circuits. Nevertheless, selective delivery of low copy number RNPs appears at odds with sustaining the localization of thousands of diverse mRNAs in the synaptic neuropil with varying abundances and encoded protein functions (Cajigas et al., 2012; Ainsley et al., 2014; Farris et al., 2019). In contrast, immunoprecipitation studies from brain lysates or dissociated cultured neurons suggest that mRNAs selectively associate within larger mRNA granules that contain many different types of transcripts and RBPs, some that are selective for specific granules (Kanai et al., 2004; Elvira et al., 2006, p. 22; Fritzsche et al., 2013; Heraud-Farlow et al., 2013). Although this model seems plausible and efficient for localizing vast amounts of mRNAs, these studies are technically limited by the lack of spatial resolution and nonspecific RNA interactions. Addressing this question requires subcellular-resolution imaging of many endogenous molecules at once, which is now technically feasible using highly multiplexed, single-molecule fluorescence in situ hybridization with iterative imaging (HiPlex smFISH).

In this paper, we used single, three-color (3Plex), and HiPlex smFISH to characterize the spatial distributions of localized neuronal RNAs in the rodent hippocampal neuropil. We generally focused on neuropil localized mRNAs that are targets of FMRP (fragile X messenger ribonucleoprotein), a ubiquitous RBP involved in neuronal mRNA localization and translational regulation (Richter et al., 2015). FMRP is associated with nearly 400 localized mRNAs in the hippocampal neuropil (Sawicka et al., 2019; Hale et al., 2021). However, it remains unclear whether localized FMRP mRNA targets segregate into distinct or similarly composed RNPs, which could contribute to the diversity and/or selectivity of FMRP-RNP compositions. Here we show, based on heterogeneity in mRNA fluorescent puncta area and intensity, that 15 neuropil localized RNAs likely vary in individual transcript copy numbers, existing as either low or high copy number populations, or more frequently, as both populations within the same mRNA. Simultaneous visualization of 12 neuropil localized FMRP-target mRNAs revealed that these mRNAs are spatially organized as such that their pairwise codistribution, assessed as colocalization, is best predicted by their abundance in the neuropil. When assessing the colocalization of all 12 RNAs at once, the highly abundant mRNAs were overwhelmingly present in mRNA clusters defined by the presence of three or more mRNAs. This finding remained true when mRNA clusters were defined by the presence of FMRP protein. We further show that cell-specific differences in mRNA colocalization can largely be explained by differences in mRNA abundance. Lastly, we used simulations to show that increased mRNA abundance can achieve the colocalization levels observed in experimental images. Collectively, these data provide evidence that, for the hippocampal mRNAs studied here, mRNAs are localized to the neuropil in heterogeneously sized puncta that may reflect differences in individual mRNA copy number and display colocalization patterns that are best explained by neuropil abundance. These data from intact rodent hippocampal neuropil are generally in agreement with imaging studies from cultured neurons that show mRNAs in neuronal dendrites are present in distinctly sized RNPs (Tübing et al., 2010; Donlin-Asp et al., 2021) and dendritic mRNA spatial proximity clustering can be partially explained by their distribution (Wang et al., 2020), suggesting that both systems are subject to similar intrinsic mechanisms that favor independent localization with stochastic overlaps as opposed to coordinated assembly of these RNAs into selective multimeric granules in the neuropil.

## Materials and Methods

### Animals

An adult female Sprague Dawley rat was used for the *Arc* dilution study. Postnatal day (1) 7 male mice were used for HiPlex RNAscope experiments. Adult (8–16 weeks) male mice were used for *Shank2* and 3plex RNAscope experiments. Animals were group-housed under a 12 h light/dark cycle with access to food and water *ad libitum*. All procedures were approved by the Animal Care and Use Committee of Virginia or University of California Irvine and were in accordance with the National Institutes of Health guidelines for care and use of animals.

### Stimulation paradigm

An electroconvulsive seizure (ECS) was induced in an unanesthetized adult female Sprague Dawley rat by delivering AC current (60 Hz, 40 mA for 0.5 s). Anesthesia was induced immediately after ECS by intraperitoneal injection of 20% urethane. The rat was then placed in a stereotaxic apparatus and a stimulating electrode was positioned to selectively activate one side of the medial perforant path projections (1.0 mm anterior to transverse sinus and 4.0 mm lateral from the midline). The electrode depth was empirically determined to obtain a maximal evoked response in the dentate gyrus (DG) at a minimal stimulus intensity, typically 3–4 mm deep from the cortical surface. A recording electrode was positioned in the molecular layer of the dorsal blade of the dentate gyrus (3.5 mm posterior from bregma, 1.8 mm lateral from the midline, 3–3.5 mm from the cortical surface based on evoked responses generated by stimulation). Single test pulses were then delivered at a rate of 1/10 s for 20 min to determine baseline response amplitude. Two hours after the ECS delivery, high-frequency stimulation (HFS; trains of eight pulses at 400 Hz) were delivered at a rate of 1/10 s. After 60 min the brain was removed and flash frozen. Brains were embedded in OCT and sectioned in the coronal plane on a cryostat at 20 μm and processed for FISH as described below.

### Fluorescence in situ hybridization

The plasmid used to generate the nearly full-length (∼3 kbp) *Arc* antisense riboprobe was a gift from John Guzowski (UC Irvine) and was generated using the Ambion MAXIscript kit with premixed RNA labeling nucleotide mix containing digoxigenin-labeled UTP (Roche). Brain sections were incubated at 56°C with labeled antisense riboprobe (1–2 ng/μl) for 14–16 h. After treatment with RNase A (10 μg/ml; Sigma) and extensive washes, including a stringency wash (0.5× SSC, 30 min at 56°C), the brain sections were incubated with a horseradish peroxidase (HRP)-conjugated antibody to digoxigenin (1:400; Roche). The HRP was detected using the Tyramide Signal Amplification Fluorescence (TSA-Cy3) kit from PerkinElmer Life and Analytical Sciences. Finally, cellular nuclei were stained with DAPI, and the slides were coverslipped using Vectashield mounting media (Vector Laboratories). For the dilution experiments, a 1× saturating stock of full-length dig-labeled *Arc* probe was serially diluted with full-length unlabeled *Arc* probe at 1:2, 1:4, and 1:8.

### Quantitative analyses of *Arc* puncta number, intensity, and size

Sections processed for FISH were imaged across the molecular layer of the dentate gyrus at 63× using a confocal microscope. The diameter, count, and intensity of *Arc*-positive puncta were determined using ImageJ particle analysis function (NIH). Briefly, a 348 × 348 pixel region of interest (ROI) was selected per layer per section and positioned to minimize saturating signals (from the cell body layer) from impacting threshold-based segmentation. To compare Arc puncta diameter and count in ECS versus ECS + HFS undiluted (1×) images, ROI cropped images were set to a common threshold calculated as the average automated maxEntropy threshold across ROIs per section (inner, middle, and outer molecular layer ROIs from ECS and ECS + HFS hemispheres). To compare puncta count in the ECS dilution images, ROI cropped images were set to the auto Otsu threshold per ROI and manually edited to reflect ground truth counts. To compare puncta diameter in the middle molecular layer ECS dilution images, each entire image was set to the average auto Otsu threshold from the 1× images. To compare puncta count and intensity in the middle molecular layer ECS dilution images, each entire image was set to the auto Otsu threshold per image and manually edited to reflect ground truth counts. All thresholds were confirmed to show little to no detectable signal on negative control sections. Segmented individual *Arc* puncta were then subjected to particle analysis. The number, average intensity, and Feret's diameter of *Arc* puncta at each dilution were averaged across three sections (technical replicates) from one animal and data are presented as mean ± SEM across sections (technical replicates).

### Single-molecule fluorescence in situ hybridization

Flash frozen brains embedded in OCT were sectioned in the horizontal plane (mouse studies) on a cryostat at 20 μm and processed for smFISH according to the RNAscope Fluorescent Multiplex or HiPlex kit instructions (Advanced Cell Diagnostics). RNAscope in situ hybridization probes can efficiently detect single mRNA transcripts (Wang et al., 2012), and smFISH RNA signals detected by this commercially available kit are strongly correlated with RNA sequencing read counts from dissected hippocampal neuropil (Farris et al., 2019). The following *Mus musculus* specific probes were used with the RNAscope fluorescent multiplex reagent kit (catalog #320850): *Rgs14* (catalog #416651), *Adcy1* (catalog #451241), *Ppp1r9b* (catalog #546311), *Shank2*-O2 Pan (catalog #513711, NM_001113373.3/ENSMUST00000105900.8), *Shank2*-O3 Short 2a (catalog #851661-C2, ENSMUST00000146006.2/NM_001113373.3), *Shank2*-O4 long 2e (catalog #852961-C3, ENSMUST00000105900.8/NM_001081370.2), mouse 3 plex positive control (catalog #320881), 3 plex negative control (catalog #320871). *Mus musculus* specific probes used for HiPlex assay (catalog #324400) include *Adcy1* (catalog #451241-T1), *Aco2* (catalog #1120581-T2), *Psd* (catalog #449711-T3), *Dlg4* (catalog #462311-T4), *Calm1* (catalog #500461-T5), *Bsn* (catalog #1119681-T6), *Camk2a* (catalog #445231-T7), *Pum2* (catalog #546751-T8), *Ddn* (catalog #546261-T9), *Pld3* (catalog #507241-T10), *Ppfia3* (catalog #1119691-T11), *Cyfip2* (catalog #561471-T12), HiPlex positive control (catalog #324321), and negative control (catalog #324341).

### RNAscope *Shank2* smFISH, image acquisition, and analysis

*Shank2* smFISH was performed according to the instructions provided in the kit (catalog #320851) using 20 µm sections from *N* = 3 mice (adult, all male). Probes labeling *Shank2e*-long, *Shank2a*-short, and *Shank2-*pan in the hippocampal CA2 cell body region were imaged with Alexa-647, Atto-555, and Atto-488, respectively. ROIs captured from CA2 cell body were 211 µm × 211 µm in *x*–*y* plane and 5 µm in *z* (25 steps, step size: 0.21 µm) at 63× magnification (numerical aperture 1.4) using a Leica Thunder (Leica DMi 8) wide-field epifluorescence microscope. *Z*-stack images were exported as 16 bit TIFF and denoised and deconvolved using NIS Batch Denoise.ai (v5.21) and NIS Batch Deconvolution (v5.21) software. Richardson–Lucy deconvolution algorithm was used to increase signal-to-noise ratio and remove background noise. After all computational processing steps for signal optimization, maximum projection images were used for further analysis in NIS Elements AR (v5.41.01). A binary segmentation layer was created using the “bright spot” command in NIS AR based on the fluorescent intensity of the smFISH puncta. Segmentation was based on intensity threshold chosen according to the corresponding negative control image such that the same threshold would result in <1–3% signals detected on the negative control image. Segmentation allowed each mRNA puncta to be considered as a segmented 2D binary object for subsequent “object-based colocalization” analysis.

#### Colocalization analysis

To quantify colocalized mRNA puncta in between two individual channels, the “having” command in NIS elements AR software (v5.41.01) was used to create a new intersection binary layer that includes any object in channel 1 that overlaps any object in channel 2 by at least 1 pixel. This binary layer was then used to count the number of overlapping puncta between two mRNA channels. Thus, colocalization was defined as any segmented mRNA puncta in channel 1 having at least 1 pixel overlap with any segmented mRNA puncta in channel 2. This default 1 pixel overlap within the “having” command is noneditable. For spot-checking, we also applied the reverse binary layer (channel 2 puncta having channel 1 puncta) and observed a similar number of colocalized puncta in between the two channels. The number of colocalized mRNA puncta was then expressed as the percentage of overlapping puncta relative to the total number of puncta for that individual channel of interest. Example equation is as follows:

$$\begin{array}{rl} & \mathrm{Percentage}\;\mathrm{of}\;Shank2a\;\mathrm{puncta}\;\mathrm{that}\;\mathrm{overlaps}\;\mathrm{with}\;Shank2-\mathrm{pan}\;\mathrm{puncta}=\\ & \frac{\mathrm{No}.\;\mathrm{of}\;Shank2a\;\mathrm{puncta}\;\mathrm{that}\;\mathrm{overlaps}\;\mathrm{with}\;\mathrm{any}\;\mathrm{puncta}\;\mathrm{in}\;Shank2-\mathrm{pan}\;\mathrm{channel}}{\mathrm{Total}\;\mathrm{number}\;\mathrm{of}\;Shank2a\;\mathrm{puncta}}\;X\;100. \end{array}$$

To quantify the number of *Shank2*-pan mRNA puncta that overlaps with any puncta from *Shank2a* and/or *Shank2e* channel, we first created a union binary layer that included all mRNA puncta in these two channels (*Shank2a* union *Shank2e*) and then created an intersection binary layer using *Shank2*-pan *having* (*Shank2a* union *Shank2e*) that would include only those *Shank2*-pan puncta with at least 1 pixel overlap with any puncta of the (*Shank2a* union *Shank2e*) binary layer.

#### Random colocalization

To calculate the percentage of puncta that would be randomly colocalized (Dunn et al., 2011; McDonald and Dunn, 2013; Hildyard et al., 2020; Sauerbeck et al., 2020), one image from each pair was rotated 180° due to the diagonal orientation of the CA2 cell body layer in the acquired images and then the binary layers were created, as was done for properly registered images, to quantify colocalized mRNA puncta presented as “random.” Paired one-tailed *t* tests were performed due to a priori expectation that the experimental colocalization would be greater than the random overlap. It is worth mentioning that colocalization in fluorescence microscopy can be quantified using pixel-based correlation coefficients or object-based segmentation methods (Bolte and Cordelières, 2006; Dunn et al., 2011). Global intensity-based correlation coefficients, such as Pearson's and Manders’, are susceptible to background noise and uneven illumination and can only detect relationships between two channels of interest. Since our objective was to assess colocalization—i.e., the spatial relationship between at least two and up to 12 different smFISH signals detected in separate channels—we opted for an object-based approach. The distinct architecture and margins of discrete fluorescent spots in deconvolved high-resolution multiplex smFISH images provided an advantage, enabling effective segmentation of signals for subsequent object-based colocalization metrics. Further, due to the scale of our dataset (66 pairwise quantification for 12 mRNA channels) and our goal to quantify multitranscript containing RNPs in addition to pairwise colocalization, we chose the semiautomated pixel overlap approach within NIS elements (defined above) as opposed to the commonly used centroid-to-centroid distance-based calculation of colocalization (Batish et al., 2012; Eliscovich et al., 2017). Although, for a subset of our data, we performed the 2D centroid distance-based colocalization as described below for the 3plex dataset analysis.

### RNAscope HiPlex smFISH

HiPlex smFISH was performed on slide mounted 20 µm sections from *N* = 4 mice (P17, all male, 2 mice per run) using RNAscope HiPlex Assay V2 (catalog #324400). After fixation, samples were dehydrated in 50, 70, 100, and 100% ethanol and treated with protease IV for 30 min at room temperature. Samples were hybridized at 40°C with the 12 probes for 2 h followed by signal amplification steps. T1–T4 fluorophores were added to label *Adcy1*, *Aco2*, *Psd*, and *Dlg4* mRNAs in round 1. For each round, 488, 550, 647, and 750 nm LED were used to image four mRNAs at 63× magnification (numerical aperture 1.4). A Leica Thunder epifluorescence microscope (Leica DMi 8) was used for imaging with recorded stage positions to acquire the same ROIs across rounds. Individual channel acquisition parameters were selected to optimize signal per mRNA in the experimental images with <1–3% of the number of experimental puncta in the associated negative control images per channel per round. *Adcy1* mRNA was labeled in round one to identify area CA2, and the DAPI signal, acquired using a 390 nm LED, was used to anatomically identify area CA1 and DG. After round one, coverslips were taken off by keeping slides in 4× SSC; fluorophores were cleaved and FFPE reagent was used to decrease background. Subsequently, T5–T8 fluorophores were added to image *Calm1*, *Bsn*, *Camk2a*, and *Pum2* in the second round. This was followed by similar steps of cleaving the fluorophores and background removal. For the final round, T9–T12 fluorophores were added to image *Ddn*, *Pld3*, *Ppfia3*, and *Cyfip2* mRNAs. Exposure was adjusted in each round matching with the expression of individual mRNAs but kept consistent across all animals per run (*N* = 2 mice per run for 2 separate runs). After completion of imaging round three, fluorophores were cleaved, and blank slides were imaged without any fluorophores. Slides were washed in TBS for 2 × 5 min, blocked in TSA blocking solution for 30 min, and incubated with anti-rabbit-FMRP primary antibody (1:100, Abcam, catalog #ab17722, Lot# 632949982) at 4°C for 48 h. Subsequently, slides were washed in TBS-T (0.05% Tween) three times for 5 min and 2% H_2_O_2_ in TBS for 10 min at room temperature. Following that, slides were incubated with goat-anti-rabbit HRP (1:250, Jackson ImmunoResearch, catalog #111035144, Lot# 149770) for 2 h at room temperature. Slides were washed in TBS-T before they underwent incubation with TSA-Cy3 (1:50, catalog #NEL704A001KT, Lot# 210322048) for 30 min at room temperature. Slides were then washed in TBS-T several times and coverslipped with prolong gold antifade mounting medium (refractive index 1.51). FMRP immunostaining was done on *N* = 2 animals, imaged using a 550 LED, and acquisition parameters were adjusted to minimize signal in the (no primary) negative control slide. Images from distal neuropil of CA2 were 211 µm × 211 µm in *x*–*y* plane and 5 µm in *z*-plane (step size 0.21 µm) at 63× magnification.

### RNAscope HiPlex smFISH image analysis

All CA2 distal neuropil *z*-stack images of individual channels and rounds were exported and postprocessed similarly as described above for *Shank2* images. Images were then maximum projected and registered using ACD RNAscope HiPlex image registration software (version 1.0.0) based on the DAPI signal of each round. After registration, a composite image of 12 mRNA channels and a DAPI channel (plus the FMRP channel for *N* = 2 mice) was created for segmentation in NIS Elements AR software. Experimental images and negative control images were processed identically at acquisition and postprocessing. Each channel was segmented to a binary layer using thresholding. The lower and upper bound of the intensity interval for thresholding was decided based on the fluorescence intensity of the entire processed experimental image and corresponding negative control image (bacterial gene *DapB* with T1–T12 channel specific fluorophores) such that the negative control produced little to no segmented objects (<3% of detected puncta of the experimental image). Resulting binaries were then manually edited to best represent the ground truth. Any segmented fluorescent puncta overlapping DAPI + 10% surrounding area was removed from the further analysis to examine only neuropil localized mRNA puncta.

#### HiPlex puncta area analysis

Fluorescent puncta area data was exported per mRNA channel per mouse (*N* = 4 mice) and then plotted as relative % histograms per mouse using the same bin width in GraphPad Prism (v10). Although there were differences in the number of mRNA puncta detected for each mRNA per mouse per run, the relative % area distribution was consistent per mRNA across mice and therefore, averaged across mice for plotting on the heatmap. To hierarchically cluster the data based on similarities in their puncta area distribution patterns, we imported the average relative % area distribution data for all 12 mRNAs written as rows in a csv file to R (v4.2.2) and used “manhattan” distance (function: “dist” in R base package) and “ward.D2” clustering (function “hclust” in R base package) technique. The “manhattan” distance metric was used because of its robustness on data with skewed or non-normal distributions. Heatmaps were generated in R using the ComplexHeatmap package (v2.15.2). We describe each of the four clusters by their peak relative % area bin (0.1–1.0 µm^2^) and the average total relative % across area bins 0.6–1.0 µm^2^ and >1.0 µm^2^. The data are presented as the average relative % ±SEM of the RNAs per cluster.

#### HiPlex puncta intensity analysis

To assess fluorescent puncta intensity distributions, we pasted the segmented puncta binary layer from the processed images onto the raw images and exported the mean intensity (average pixel intensity in each punctum) and total fluorescent intensity (total of all pixel intensities in that punctum) of each binary object from each channel for *N* = 2 mice (1 mouse per HiPlex run). Although similar probe designs (20 zz pairs, ∼1,100 basepair target region length) were used for each mRNA, the range of total intensities per puncta varied based on image acquisition parameters that varied per mRNA per experimental run. To compare fluorescent puncta intensities across mRNAs, we divided the puncta total intensity value by the mean intensity of the puncta in each mRNA channel that resulted in a normalized range of total intensity per puncta. For each individual punctum, the normalized total intensity theoretically should give an arbitrary value that correlates with the area of the puncta on a given image (9.6679 pixels per µm). Therefore, we then correlated raw and normalized total intensity values with puncta areas. Next, we plotted the relative % intensity distributions with the same bin width for all 12 mRNAs. The average relative % distribution of normalized total intensity per puncta of each mRNA was then plotted on a heatmap. The same hierarchical clustering (distance: manhattan, clustering: ward.D2) was used as above to identify mRNAs with similar puncta intensity distributions.

#### HiPlex colocalization analysis

Four 52 × 52 µm^2^ ROIs were cropped from the 211 × 211 µm^2^ image. For each mRNA, subsequent binary layers were created, using the “having” command as used above for the *Shank2* analysis, that would contain only mRNA puncta from the channel of interest having at least 1 pixel overlap with puncta from each of the other 11 mRNA channels in consideration. For example, 11 new binary layers were created for *Psd* mRNA channel, each of which included only those *Psd* mRNA that overlaps with one of the other eleven mRNA channels (e.g., “*Psd* having *Camk2a*” binary layer only includes *Psd* mRNA puncta that overlaps with *Camk2a* mRNA puncta). These binaries were used to calculate the number of overlapping mRNA puncta for each pair, which was then expressed as a percentage of the given mRNA and as a fraction of the pair of mRNAs, plotted separately as heatmaps as described above.

For the quantification of the random level of overlaps, the same method of creating binaries and quantification was followed only after rotating the image of the given mRNA to 90° right as well as 180° (e.g., “rotated *Psd* having *Camk2a*” binary layer only included *Psd* mRNA puncta from rotated *Psd* image that overlaps with any *Camk2a* mRNA puncta by at least 1 pixel). Both 90 and 180° rotated images resulted in a similar percentage of random overlap so only data from 90° rotated images are shown. Percent random colocalization (from rotated images) was subtracted from the percentage of colocalization calculated from experimental images for each RNA and plotted as a heatmap as above. Hierarchical clustering was performed as described above for the puncta analyses, except that “euclidean” distance was used to identify RNAs with overall similar colocalization profiles.

#### Psd mRNA puncta composition analysis

Calculation of *Psd* % colocalization with any mRNA in the HiPlex dataset was done by creating a union layer of all intersect binary layers for *Psd*. Thus “*Psd* having *Camk2a* (mRNA 1)”, “*Psd* having *Ddn* (mRNA 2)”…“*Psd* having *Ppfia3* (mRNA 11)” layers were merged to create a union layer that includes *Psd* mRNA puncta with at least 1 overlapping pixel with puncta from any of the other 11 channels. This step was repeated for rotated *Psd* mRNA images across all four mice. To calculate *Psd-Camk2a* multimers, we created an intersect layer using the “having” command to include colocalized *Psd-Camk2a* puncta. Then, we created a separate union binary layer that includes *Psd* mRNA having overlaps with the other nine mRNAs combined. Finally, we created an intersection binary layer of these two layers that subsetted the *Psd* mRNA puncta that had overlaps with *Camk2a* and at least another of the nine mRNAs. These steps were then similarly executed on the rotated *Psd* image. The resulting data were expressed as percentage of *Psd* puncta and plotted as experimental and random pie charts (mean ± SEM, *N* = 4 mice) using GraphPad Prism (v10).

#### Correlation plots of colocalization and abundance

We calculated the average % colocalization of *Psd* with each of the other 11 mRNAs and the average abundance of those mRNAs and plotted the correlation in GraphPad Prism (v10). The correlation of % colocalization with mRNA abundance was similarly calculated for *Ddn* and *Pum2*.

#### FMRP-defined Psd puncta colocalization analysis

Additional binary layers were created per channel as above including only mRNA signals that colocalized with FMRP. Pairwise colocalization and *Psd* mRNA composition analysis were repeated similarly on this subset.

### RNAscope 3plex (*Rgs14*, *Adcy1*, and *Ppp1r9b*) smFISH

*Rgs14*, *Adcy1*, and *Ppp1r9b* smFISH was performed using RNAscope fluorescent multiplex reagent kit (catalog #320851) as described in the kit protocol using 20 µm sections from *N* = 4 adult mice (all male). A total of 211 µm × 211 µm ROI (in *x*–*y* plane) of 5 µm thickness *Z*-stack images (25 steps, step size 0.21 µm) were acquired by a Leica thunder (Leica DMi 8) wide-field fluorescence microscope at 63× magnification (numerical aperture 1.4). CA2 was identified using *Rgs14* and *Adcy1* mRNA labeling. *Rgs14*, *Adcy1*, and *Ppp1r9b* were imaged using 488, 550, and 647 nm LEDs. CA1 and CA2 proximal and distal neuropil regions and the DG molecular layer were imaged from *N* = 4 adult mouse hippocampus. Exported TIFF images were then denoised and deconvolved as described above. In NIS Elements AR, a binary segmentation layer was created per channel, using intensity threshold of the experimental and corresponding negative control image and manually edited to best represent the data (similar to the thresholding process in HiPlex dataset). Any mRNA puncta overlapping plus 10% of DAPI was excluded from quantification to confirm only mRNAs in the neuropil but not in glia or interneurons are included in the analysis except for the simulation analysis where all detected signals and their *x–y* coordinates were included as described below. After manually editing each binary layer, the number of mRNA puncta and fluorescent puncta area data was exported to Excel and plotted with Prism.

### RNAscope 3Plex smFISH image analysis

A total of 180 µm × 180 µm ROI was cropped from CA2, CA1, and DG images for object-based colocalization analysis on binary segmented images. Similar to previous analyses, the “having” command was used in NIS Elements to define colocalized objects (at least by 1 pixel) between two separate channels of interest. The number of mRNA puncta that colocalized between two channels was then divided by the total number of mRNA puncta in both channels and plotted as a percentage.

#### Centroid-based colocalization analysis

To compare lenient (>1% overlap) versus stringent (>50% overlap) definitions of colocalization, we exported puncta centroid *x–y* coordinates (geometric center of each puncta), puncta area, and Feret's diameter from NIS Elements for DG molecular layer images from *N* = 4 mice. We chose images from DG because this subregion had sufficient puncta in all three mRNA channels that would result in a non-0 number of observations with the stringent definition of colocalization. There is no customizable function to define the degree of colocalization in NIS Elements. So, we executed the subsequent analysis in Python using NumPy, panda, and scipy.spatial packages (Virtanen et al., 2020). Centroid to centroid 2D distance between two puncta from two channels was calculated using “cdist” function from scipy.spatial package. Colocalization was defined as >1% overlap [if the distance between centroid *x–y* coordinates of two puncta from two channels is less than 0.99 × sum of radii (Feret's diameter / 2) of each puncta pair] and >50% overlap [if the distance between centroid *x–y* coordinates of two puncta from two channels is less than 0.50 × sum of radii (Feret's diameter / 2) of each puncta pair] as a syntax using NumPy in Python. The number of mRNA puncta that colocalized between two channels was then expressed as a fraction of the pair of mRNAs and as a percentage of the given mRNA, plotted separately as bar plot and heatmap.

#### Simulated colocalization analysis

The *Adcy1* mRNA puncta simulation analysis was done in Python (3.9.13) using pandas, NumPy, and scipy.spatial packages (Virtanen et al., 2020) in spyder (5.2.2). We exported puncta centroid *x–y* coordinates, puncta area, Feret's diameter, and total intensity per puncta from CA2 proximal dendrite and DG molecular layer images (*N* = 4 mice, 180 × 180 µm^2^ ROI per mouse). These images were segmented similarly as above except without removal of puncta overlapping DAPI signal (interneuron clusters) to avoid bias in the specific *x–y* coordinates in randomly simulated data. A list of synthetic data points (the number of which equaled the difference of Adcy1 mRNA puncta between CA2 and DG images in the same section) was generated from bootstrap-sampled values from the minimum-to-maximum range for CA2 image puncta area, Feret's diameter, and total intensity per puncta using “sample(*n* = …, replace = True).values” function. Centroid (*x*,*y*) coordinates for these synthetic data points were uniformly drawn at random (without replacement) within the 0–180 µm *x* and *y* ROIs using “np.random.uniform” function (no duplicate centroid coordinates were used, either in the randomly generated values or the experimental dataset). Then, this simulated data was appended to the CA2 experimental dataset to generate the CA2 simulated dataset. We plotted CA2 experimental and simulated data points using the “sns.scatterplot” function to visually inspect that the *x–y* coordinates were added randomly. Ten bootstrapped iterations were performed for each CA2 proximal dendrite image and the % colocalization of *Adcy1/Ppp1r9b* mRNAs (defined as >1% overlap as above) per iteration was averaged to generate a single value per image that was compared with the CA2 and DG experimental images.

#### 3Plex puncta area analysis

Similar to HiPlex puncta area analysis described above, fluorescent puncta area data of *Rgs14*, *Adcy1*, and *Ppp1r9b* mRNAs from proximal and distal neuropil CA2 and CA1 images, and DG molecular layer images (211 µm × 211 µm ROIs) were first plotted as individual relative % histograms in Prism for each animal (data not shown). Due to the consistent pattern of area distribution for each mRNA across animals, data from proximal and distal neuropil were combined to represent one population for CA2 and CA1 neuropil. The average relative % area distribution (*N* = 4 mice) for each mRNA in each cell type (CA1, CA2, and DG) was then plotted as a heatmap with hierarchical clustering as described above.

### Statistical analyses

All statistical tests were two-tailed except for comparison of experimental and random colocalization which were one-tailed due to the a priori hypothesis that experimental colocalization would be higher than randomly colocalized puncta. When ANOVA statistical tests are reported, Tukey's or Dunn's multiple-comparison post hoc tests are shown on the figure plots and described in the figure legend accordingly. When multiple *t* tests are reported, the two-stage step-up method of Benjamini, Krieger, and Yekutieli with 1% false discovery rate (FDR) was used for correcting for multiple comparisons. All statistical analyses were done using GraphPad Prism (v10) with a significance level of 0.05 or lower (*α* = 0.05).

### Code accessibility

No custom code was generated to support the conclusions in the manuscript. Established Python and R packages and versions are listed in the methods. Raw and processed imaged files and RNA puncta coordinates used to generate the simulated abundances used in Figure 5 are available in the VT data repository: https://doi.org/10.7294/30479759.v1.

## Results

### *Arc* mRNA puncta contain multiple copies of *Arc* transcripts

**Figure 1.**
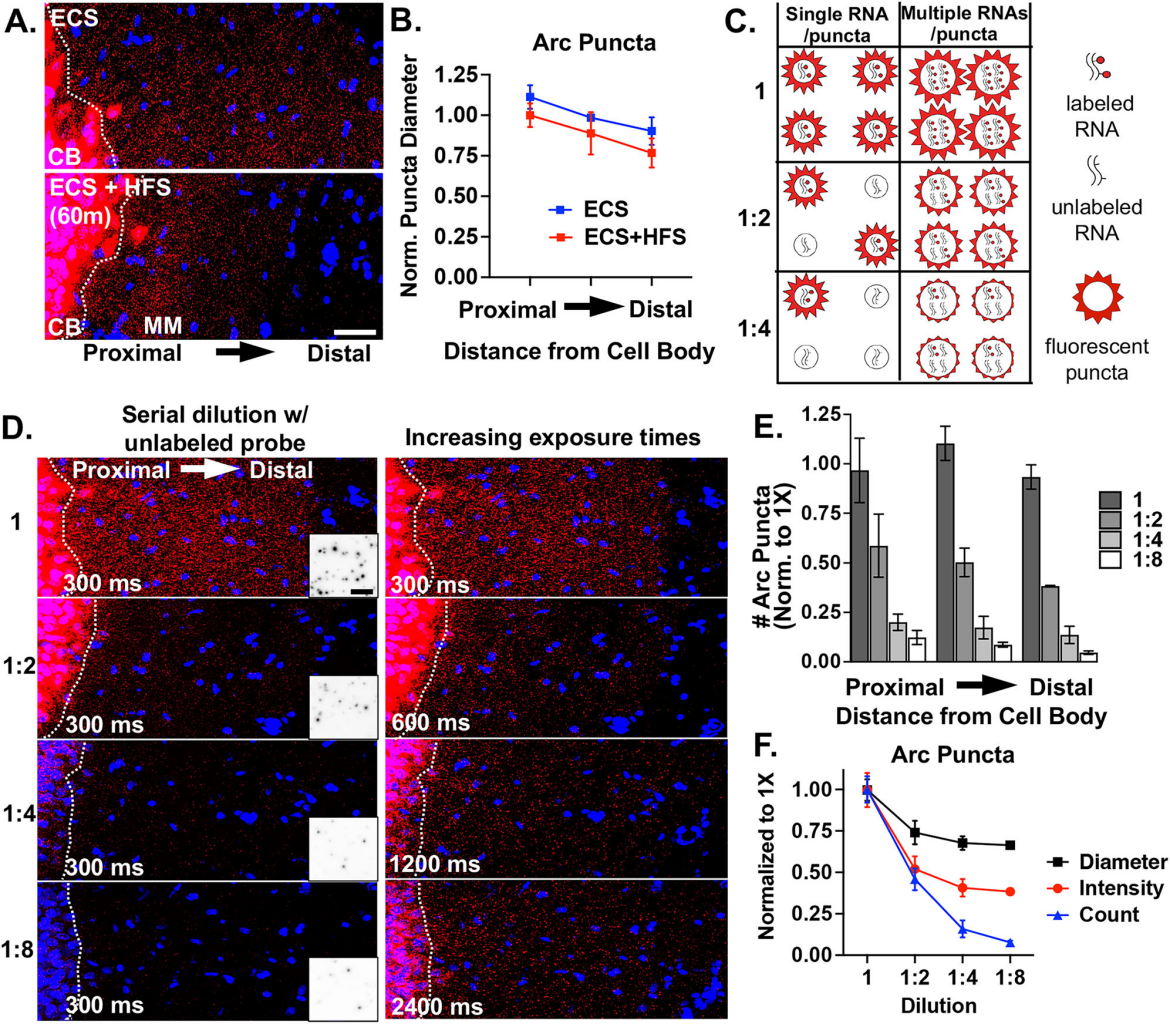
Arc mRNA fluorescent puncta diameter, number, and intensity upon probe dilution reveal multiple pools of mRNAs. ***A***, Representative images of Arc mRNA localization to the middle molecular layer (MM) of the rat dentate gyrus following ECS and 60 min unilateral HFS. The fluorescence signal in the cell body layer is saturated to visualize the fluorescence signal in the dendrites. Scale bar = 25 µm. ***B***, Quantification of the Feret's diameter of localized Arc mRNA puncta (HFS condition) versus nonlocalized puncta (ECS) by distance from the cell body layer (CB, white dashed lines). Average diameter (normalized to ECS) ± SEM per layer from *n* = 3 technical replicates. ***C***, Possible outcomes of serial probe dilution on single and multiple copy number Arc puncta in terms of number and intensity or apparent size of fluorescent puncta. ***D***, Representative images after ECS only labeled with 1× undiluted full-length Arc probe or serially diluted with unlabeled full-length Arc probe and imaged with identical exposure times, 300 ms (left). Images acquired with doubling exposure times (600, 1,200, 2,400 ms) revealed undetected puncta at 300 ms (right), indicating a decrease in puncta intensity as would be expected with multiple RNAs per puncta. Inverted inset scale bar, 2.5 µm. ***E***, Quantification of Arc puncta number for each dilution at 300 ms (normalized to 1×). Stepwise decrease in Arc puncta number suggests low copy number-containing puncta. Average number ± SEM per layer from *n* = 3 technical replicates. ***F***, Quantification of Arc puncta Feret's diameter, intensity, and number for the middle molecular layer of each dilution at 300 ms. Normalized average ± SEM from *n* = 3 technical replicates. Representative MM layer inverted images inset in ***D***.

To begin to address whether neuronal mRNAs are localized in the neuropil as low- and/or multiple copy number-containing mRNA puncta, we investigated the RNA properties of the well-known neuropil localized mRNA *Arc* (activity-regulated cytoskeleton-associated mRNA). *Arc* mRNA expression in the hippocampal dentate granule (DG) cell dendrites is unique in that it is tightly regulated by activity-dependent transcription and degradation (Farris et al., 2014). At baseline, dendritic *Arc* expression is low or absent in most DG cells, but after a single ECS (ECS), *Arc* mRNA is rapidly transcribed (within ∼3 min) and transported throughout the DG dendritic laminae by 30 min to 1 h (Steward et al., 1998). Given the short half-life of *Arc* mRNA (∼45 min; Rao et al., 2006), the prolonged presence of *Arc* mRNA in DG dendrites (e.g., at 2 h) is maintained by ongoing transcription and dendritic transport (Farris et al., 2014). Subsequent unilateral HFS of the entorhinal cortical perforant path inputs to the DG further boosts *Arc* transcription and leads to the accumulation of newly transcribed *Arc* mRNA selectively near the activated synapses in the middle molecular layer and a depletion of *Arc* mRNA from the outer molecular layer (Steward et al., 1998; Farris et al., 2014). Using this stimulation paradigm (ECS + HFS) on a single adult female rat and FISH, we assessed the size of dendritically localized (ECS) and synaptically localized (HFS) *Arc* mRNA puncta to examine whether *Arc* mRNA composition changes with synaptic localization (Fig. 1). We found that *Arc* mRNA puncta are generally smaller, as measured by Feret's diameter, in the ECS + HFS condition versus ECS condition, although there is considerable variability across replicates (Fig. 1*A*,*B*, average from three technical replicates). Moreover, Arc puncta are generally larger in proximal versus distal dendrites under both conditions. These data suggest that resident and newly transcribed dendritic *Arc* mRNA puncta contain a similar amount of *Arc* mRNA per puncta despite differences in the number of puncta.

Next, in order to assess the transcript occupancy of *Arc* puncta, we measured fluorescent puncta diameter and number on the contralateral ECS hemisphere (dendritically localized) after serial dilution of 1× labeled full-length *Arc* probe with unlabeled (cold) full-length *Arc* probe (1:2, 1:4, 1:8). We reasoned that a stepwise decrease in *Arc* mRNA puncta number would reflect mRNAs transported singly or at low copy numbers that were no longer detectable when half, fourth, or eighth of the probe was labeled (Fig. 1*C*). Alternatively, a decrease in apparent fluorescent puncta size would reflect a reduction in the number of labeled transcripts from multiple copy-containing *Arc* mRNA puncta (Fig. 1*C*). When acquiring images using identical acquisition parameters (optimized for 1× labeled probe), we detected a stepwise decrease in *Arc* RNA puncta number, with the largest drop-offs at one-half and one-fourth cold probe dilutions (Fig. 1*D*,*E*). These data are consistent with a population of low copy number *Arc* mRNAs. However, when we doubled the exposure time after each dilution, we qualitatively saw an increase in the number of *Arc* mRNA puncta indicating that a proportion of the *Arc* mRNAs labeled with cold probe dropped below the detection threshold (Fig. 1*D*). This is in agreement with the measured decrease in fluorescence intensity and apparent size (Feret's diameter) of *Arc* mRNA puncta between undiluted and diluted conditions (Fig. 1*F*), which we interpret to reflect a population of *Arc* mRNA puncta containing multiple copies of *Arc* transcripts. Collectively, these data suggest that there are multiple populations of *Arc* mRNAs, those with both low and high *Arc* copy numbers that exist in DG dendrites.

### smFISH probes can detect mRNA colocalization

**Figure 2.**
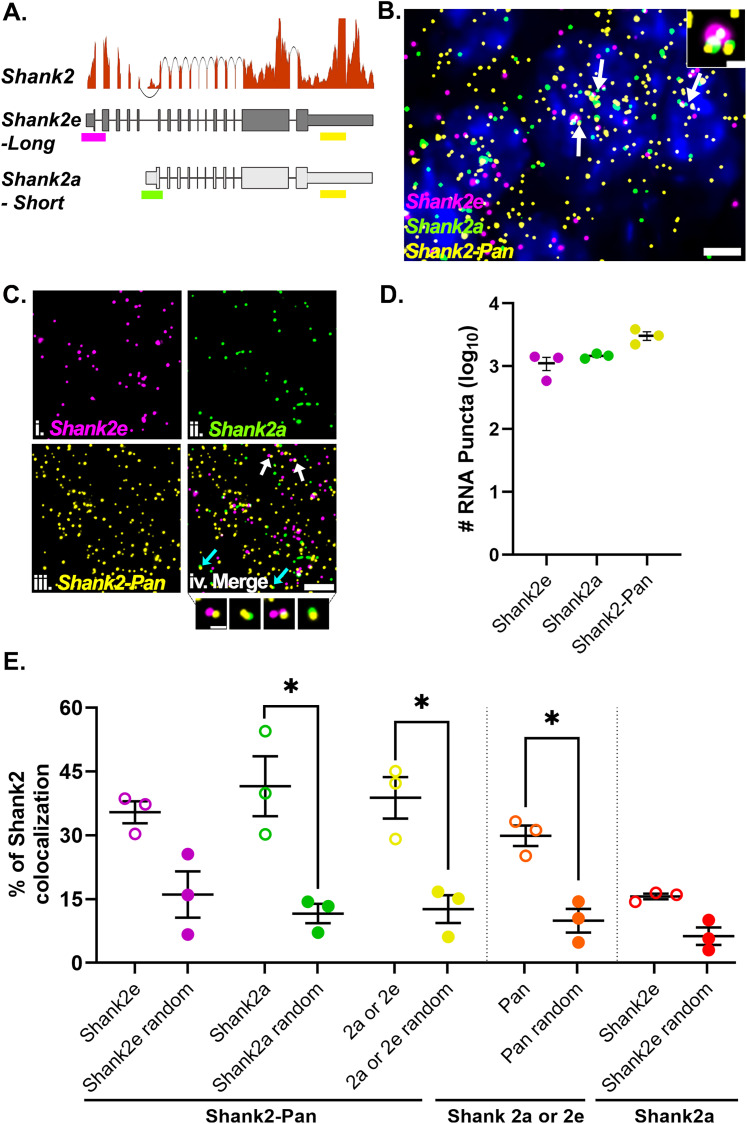
*Shank2* isoform-specific 5′ probes are highly colocalized with the Pan 3′ probe. ***A***, *Shank2* isoform gene models with RNAseq read depth data showing the relative expression levels in hippocampal CA2. Sequences from either long (*Shank2e*) or short (*Shank2a*) transcripts targeted by different 5′ probes (magenta and green, respectively) and both targeted by the Pan 3′ probe (yellow) are shown. ***B***, Representative image of the three *Shank2* probes in CA2 cell bodies. Nuclei are labeled with DAPI (blue). White arrows indicate example transcriptional foci. Dashed white box is the inset showing a transcriptional focus labeled by all three probes. ***C***, High-magnification images of (***i***) *Shank2e*, (***ii***) *Shank2a*, (***iii***) *Shank2*-Pan, and (***iv***) the merged image. Arrows indicate example colocalization of *Shank2*-Pan 3′ probe with either *Shank2e* 5′ probe (white arrows) or *Shank2a* 5′ probe (cyan arrows) as shown below. ***D***, *Shank2e*, *Shank2a*, and *Shank2*-pan mRNA puncta count in the CA2 cell body layer. ***E***, Quantification of the % colocalization between the *Shank2e* (magenta) and *Shank2a* (green) or both (yellow) with the *Shank2*-Pan probe (open circles) compared with that observed by random colocalization (closed circles). % of *Shank2*-Pan colocalized with either *Shank2a* or *Shank2e* (orange) compared with random colocalization and the % of *Shank2e* colocalizing with *Shank2a* (red) compared with random, many of which are transcription foci, as shown in ***B***. Error bars indicate SEM; *N* = 3 mice; *denotes *p* < 0.05 from paired one-tailed *t* test. Scale bars: (***B***) 5 µm, 1 µm, (***C***) 5 µm, 1 µm.

In addition to neuropil localized RNAs consisting of low or multiple copies of the same mRNA (homotypic), we wanted to test whether they are composed of multiple species of mRNAs (heterotypic) as described in situ for established RNA granules, like germ plasm granules (Trcek et al., 2015) or p-bodies (Cougot et al., 2008; Ford et al., 2019) and identified biochemically for neuronal transport granules (Elvira et al., 2006; Fritzsche et al., 2013; Heraud-Farlow et al., 2013; Fatimy et al., 2016). In order to assess the colocalization of different mRNA transcripts into common RNA puncta, we first needed to confirm that we can reliably detect colocalized smFISH signals. To test this, we took advantage of the fact that there are (at least) two isoforms of *Shank2* expressed in hippocampal area CA2 (Farris et al., 2019). The two isoforms are generated via alternative 5′ promoters and thus differ in their 5′ untranslated regions (UTRs) but have identical 3′ UTRs (Jiang and Ehlers, 2013; Monteiro and Feng, 2017). Using isoform-specific probes targeted to the two distinct 5′ UTRs (*Shank2e*-long and *Shank2a*-short) and a pan *Shank2* probe targeted to the common 3′ UTR (*Shank2*-pan; Fig. 2*A*), we calculated the percentage of colocalized signals, defined as puncta in separate channels overlapping by at least 1 pixel. In agreement with RNAseq expression data (Fig. 2*A*), we detected *Shank2* expression from all three probes in area CA2 (Fig. 2*B*,*C*). In general, the *Shank2*-pan probe detected more *Shank2* mRNAs than the 5′ UTR probes combined (# of mRNA: *Shank2*-pan = 3,056 ± 472, *Shank2e* = 1,122 ± 268, *Shank2a* = 1,463 ± 79, *N* = 3 mice; Fig. 2*D*), either due to the (presumed) greater accessibility of the 3′ UTR from less RNA secondary structure compared with the 5′ UTRs, or potentially due to expression of other isoforms that include the 3′ UTR but not either of the two 5′ UTRs (e.g., *Shank2C*; Monteiro and Feng, 2017) that cannot be resolved via short-read sequencing. We found that nearly 40% of *Shank2e* (35.41 ± 2.57%) or *Shank2a* (41.53 ± 7.05%) colocalized with the *Shank2*-pan probe (Fig. 2*E*). The *Shank2*-pan probe also colocalizes with either 5′ UTR probe at ∼30% (29.88 ± 2.42%). The fact that this relative percentage is not greater than the colocalization of the individual 5′ UTR colocalization is due to both the greater number of *Shank2*-pan labeling (described above) and several instances where all three probes colocalized at presumed transcriptional foci (Fig. 2*B*, inset). These data are consistent with previous findings, where ∼30% of 5′ and 3′ Arc mRNA probes colocalize in the dendrites of dentate granule cells in rats (Farris et al., 2014). Thus, we reason that RNAscope smFISH is more limited in its ability to detect colabeling of the same individual RNA transcript with two probes (∼30%; Farris et al., 2014; Hildyard et al., 2020) perhaps due to steric hindrance or competition of the DNA-based labeling approach, but it is highly likely to detect colocalization when more than one transcript is being labeled (e.g., two transcripts of the same RNA or two distinct neuropil localized RNAs).

To account for the amount of colocalization expected to occur by randomly overlapping puncta, which is also influenced by expression levels, we rotated one of the channels from each probe pair 180° and remeasured “random” colocalization (Dunn et al., 2011; McDonald and Dunn, 2013). Image rotation is a validated technique for assessing random colocalization of synaptic molecules in the hippocampal neuropil (Sauerbeck et al., 2020; Frye et al., 2021). Here, because the cell body layer is in a diagonal orientation, we rotated 180° instead of the commonly used 90°. In most cases, we detected a significantly greater % colocalization than was observed at random (Fig. 2*D*). In the instance where % colocalization is near random, as with the two 5′ probes, we assume this to indicate that these two transcripts do not colocalize often into common complexes. In summary, our method is able to reliably detect colocalization of two probes targeted to the same mRNA, which is a higher bar than for detecting two mRNAs within the same RNA puncta, which we assess below.

### Putative FMRP-target mRNAs have heterogeneous puncta area distributions

Subcellular localization of a given mRNA is assumed to be affected by the composition of the RNA–RBP complexes (Mikl et al., 2011; Mitsumori et al., 2017). Once we demonstrated that our method can reliably detect colocalization when we expect it, we explored whether known neuropil mRNA transcripts localize independently or in association (colocalized) with each other as heterotypic complexes of two or more RNAs. We rationalized that mRNAs with a shared RBP interactor would be more likely to demonstrate colocalization patterns reflecting some degree of selectivity in how they associate with each other, if at all. We took advantage of the relatively well-characterized RBP, FMRP, and its neuropil localized target mRNAs to quantitatively map their colocalization. We generated a list of candidate target mRNAs by cross-referencing datasets that identified hundreds of putative FMRP-target mRNAs using HITS-CLIP (high-throughput sequencing of mRNA isolated by cross-linking immunoprecipitation) on whole brain (Darnell et al., 2011) and hippocampal CA1 neuropil (Sawicka et al., 2019) with datasets that identified high-confidence hippocampal neuropil mRNAs (Cajigas et al., 2012; Ainsley et al., 2014; Farris et al., 2019). This list of neuropil localized candidate FMRP-target mRNAs was further curated based on expression, different encoded protein functions (signaling, cytoskeletal, synaptic plasticity, etc.), and target destinations (mitochondria, cytoplasm, cell membrane, dendritic spine) to further stratify colocalization patterns (Table 1).

**Table 1.** HiPlex RNA probe information (refers to Figs. 3, 4)

Probe	Gene symbol	NCBI transcript ID	Ensembl ID	UniProt	Allen Brain Atlas	Protein name (symbol)	Protein function	Subcellular localization	FMRP CLIP rank (Darnell et al., 2011)	CA2 soma expression (Log2; Farris et al., 2019)	CA2 neuropil expression (Log2; Farris et al., 2019)	Target region	Target region length_bp	Full transcript length_bp	5′ UTR _bp	3′ UTR_bp	Imaging round	Area distribution cluster in HiPlex smFISH
T1	*Adcy1*	NM_001281768.2	ENSMUSG00000020431	https://www.uniprot.org/uniprotkb/O88444/entry	https://mouse.brain-map.org/gene/show/129123	Adenylate cyclase type 1 (ADCY1)	Adenylate cyclase activity, synaptic transmission, G-protein signaling, Postsynaptic density	Cytoplasm	7	16.30	16.44	829–1,704	875	12,315	111	8,791	Round1	Large broad
T2	*Aco2*	NM_080633.2	ENSMUSG00000022477	https://www.uniprot.org/uniprotkb/Q99KI0/entry	https://mouse.brain-map.org/experiment/show?id=67978674	Aconitate hydratase, Aconitase mitochondrial (ACO2)	Essential enzyme in the tricarboxylic acid cycle, isocitrate metabolic processes, mitochondrial metabolism	Cytoplasm, mitochondrial matrix	228	14.40	14.47	1,444–2,363	919	2,785	204	371		Large broad
T3	*Psd*	NM_028627.2	ENSMUSG00000037126	https://www.uniprot.org/uniprotkb/Q5DTT2/entry	https://mouse.brain-map.org/gene/show/49569	Pleckstrin homology and SEC7 domain-containing protein 1 (PSD, also known as Exchange factor for ARF6)	Dendritic spine, postsynaptic density, phospholipid binding	Dendritic spine	297	12.63	13.76	1,167–2,308	1,141	3,950	213	612		Small broad
T4	*Dlg4*	NM_007864.3	ENSMUSG00000020886	https://www.uniprot.org/uniprotkb/Q62108/entry	https://mouse.brain-map.org/gene/show/13164	Disks large homolog 4 (DLG4, also known as PSD95)	Postsynaptic scaffolding protein, synaptic plasticity, postsynaptic density	Dendritic spine	52	14.66	15.25	1,386–2,395	1,009	3,339	476	837		Large
T5	*Calm1*	NM_001313934.1	ENSMUSG00000001175	https://www.uniprot.org/uniprotkb/P0DP26/entry	https://mouse.brain-map.org/gene/show/12098	Calmodulin-1 (CALM1)	Calcium signal transduction pathway	Cytoplasm	453	16.82	16.09	1,121–2,121	1,000	4,020	279	3,386	Round 2	Small
T6	*Bsn*	NM_007567.2	ENSMUSG00000032589	https://www.uniprot.org/uniprotkb/O88737/entry	https://mouse.brain-map.org/gene/show/12003	Bassoon (BSN)	Presynaptic scaffolding protein, protein localization to synapse	Axonal cytoplasm	1	16.14	15.4	8,020–9,090	1,070	15,953	126	3,983		Large broad
T7	*Camk2a*	NM_009792.3	ENSMUSG00000024617	https://www.uniprot.org/uniprotkb/P11798/entry	https://mouse.brain-map.org/gene/show/12107	Calcium/calmodulin-dependent protein kinase type II subunit alpha (CAMKIIa)	Calmodulin-binding, synaptic plasticity, dendritic spine development, postsynaptic density	Dendritic Spine	39	17.12	18.87	896–1,986	1,090	4,268	161	3,372		Small broad
T8	*Pum2*	NM_001160219.1	ENSMUSG00000020594	https://www.uniprot.org/uniprotkb/Q80U58/entry	https://mouse.brain-map.org/experiment/show?id=68845514	Pumilio homolog 2 (PUM2)	Cytosolic RNA-binding protein, translation regulation	Cytoplasm	398	13.14	13.35	242–1,544	1,302	6,311	275	2,841		Large
T9	*Ddn*	NM_001013741.1	ENSMUSG00000059213	https://www.uniprot.org/uniprotkb/Q80TS7/entry	https://mouse.brain-map.org/experiment/show?id=71212512	Dendrin (DDN)	Enables RNA polymerase II *cis*-regulatory region sequence-specific DNA binding activity	Cytoplasm, dendritic spine membrane	180	15.01	16.32	696–1,889	1,193	3,740	112	1,500	Round 3	Small
T10	*Pld3*	NM_001317355.2	ENSMUSG00000003363	https://www.uniprot.org/uniprotkb/O35405/entry	https://mouse.brain-map.org/experiment/show?id=77464848	Phospholipase D3 (PLD3)	Lysosomal protein, phospholipase activity,	Lysosomal membrane, endoplasmic reticulum membrane	537	14.16	13.31	1,227–2,132	905	2,317	532	298		Small
T11	*Ppfia3*	NM_029741.2	ENSMUSG00000003863	https://www.uniprot.org/uniprotkb/P60469/entry	https://mouse.brain-map.org/experiment/show?id=69202693	Liprin-alpha-3 (PTPRF-interacting protein alpha-3, PPFIA3)	Synaptic vesicle docking, presynaptic active zone cytoplasmic component	Cytoplasm	736	12.55	12.26	3,295–4,388	1,093	4,882	158	904		Large
T12	*Cyfip2*	NM_001252459.1	ENSMUSG00000020340	https://www.uniprot.org/uniprotkb/Q5SQX6/entry	https://mouse.brain-map.org/experiment/show?id=74357791	Cytoplasmic FMR1-interacting protein 2 (CYFIP2)	Actin filament reorganization, neuronal projection development	Cytoplasm	9	16.76	15.13	1,220–2,889	1,669	6,385	392	2,450		Large broad

Information in columns Target region and Target region length_bp are obtained from ACD Bio-Techne website (https://www.bio-techne.com/). Information in column Full transcript length_bp is obtained from NCBI website (https://www.ncbi.nlm.nih.gov/nuccore). Information in columns 5' UTR _bp and 3' UTR_bp are obtained from UCSC Genome browser (https://genome-euro.ucsc.edu/cgi-bin/hgGateway?hgsid=381790182_QVzlOBAPJh8GAsutlt4hv7T6yXcX).

bp, base pairs.

**Figure 3.**
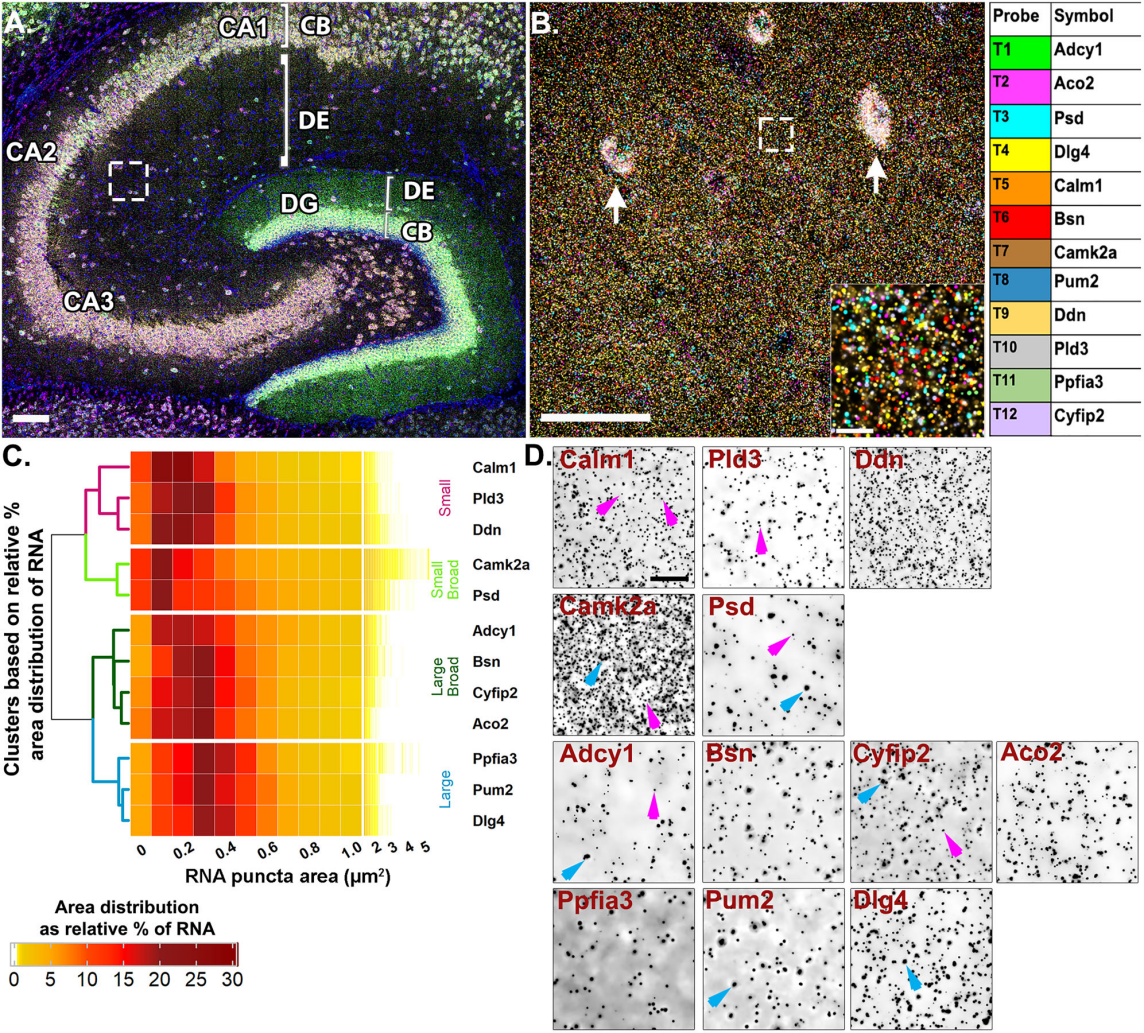
Highly multiplexed mRNA imaging reveals neuropil localized mRNAs have distinct puncta area distributions. ***A***, Representative image of mouse hippocampus with *Adcy1*, *Aco2*, *Psd*, and *Dlg4* labeling in round 1 of HiPlex smFISH. White box represents ROI from CA2 dendrites. ***B***, Representative high-magnification merged image of 12 mRNAs from CA2 dendrites. Arrows denote interneuron expression in the neuropil layer that is removed before analysis (see Materials and Methods) and inset is the dashed white box. Each mRNA is colored based on the table on the right. ***C***, Heatmap of RNA fluorescent puncta area distributions hierarchically clustered by similarity (*N* = 4 mice). ***D***, Representative inverted images of each mRNA. Magenta arrows denote small RNAs in *Calm1*, *Pld3*, *Camk2a*, *Psd*, *Adcy1*, and *Cyfip2*. Blue arrows denote larger sized RNAs in *Camk2a*, *Adcy1*, *Cyfip2*, *Ppfia3*, and *Dlg4*. Scale bar: ***A***, 100 µm; ***B***, 50 µm, 10 µm; ***D***, 10 µm (refer to Extended Data Figs. 3-1–3-4 for more details).

To spatially map the association of these putative FMRP-target mRNAs, we probed for 12 endogenous mRNAs at once and iteratively imaged four at a time using RNAscope HiPlex smFISH followed by FMRP immunostaining (Fig. 3*A*,*B*; Extended Data Fig. 3-1). We used P17 mice to coincide with the period of hippocampal synaptic maturation with the highest FMRP protein levels (Zang et al., 2009). Experimental and negative control images were postprocessed with denoising and deconvolution and each mRNA channel was segmented based on an intensity threshold that produced negligible signal in the corresponding negative control image (Extended Data Fig. 3-2). Each of the mRNAs were present in CA2 dendrites with varying degrees of abundance (Extended Data Fig. 3-3). Based on the findings from our previous experiments (Fig. 1), which indicated the presence of distinctly sized populations of homotypic RNA puncta within the same mRNA species, we first calculated the median fluorescent puncta area (Table 2) and plotted the relative % area distributions (Fig. 3*C*). Unexpectedly, we found these mRNAs vary considerably in their fluorescent puncta area distributions, which is not explained by fluorophore, imaging round, or abundance in the neuropil (Table 1).

**Figure 4.**
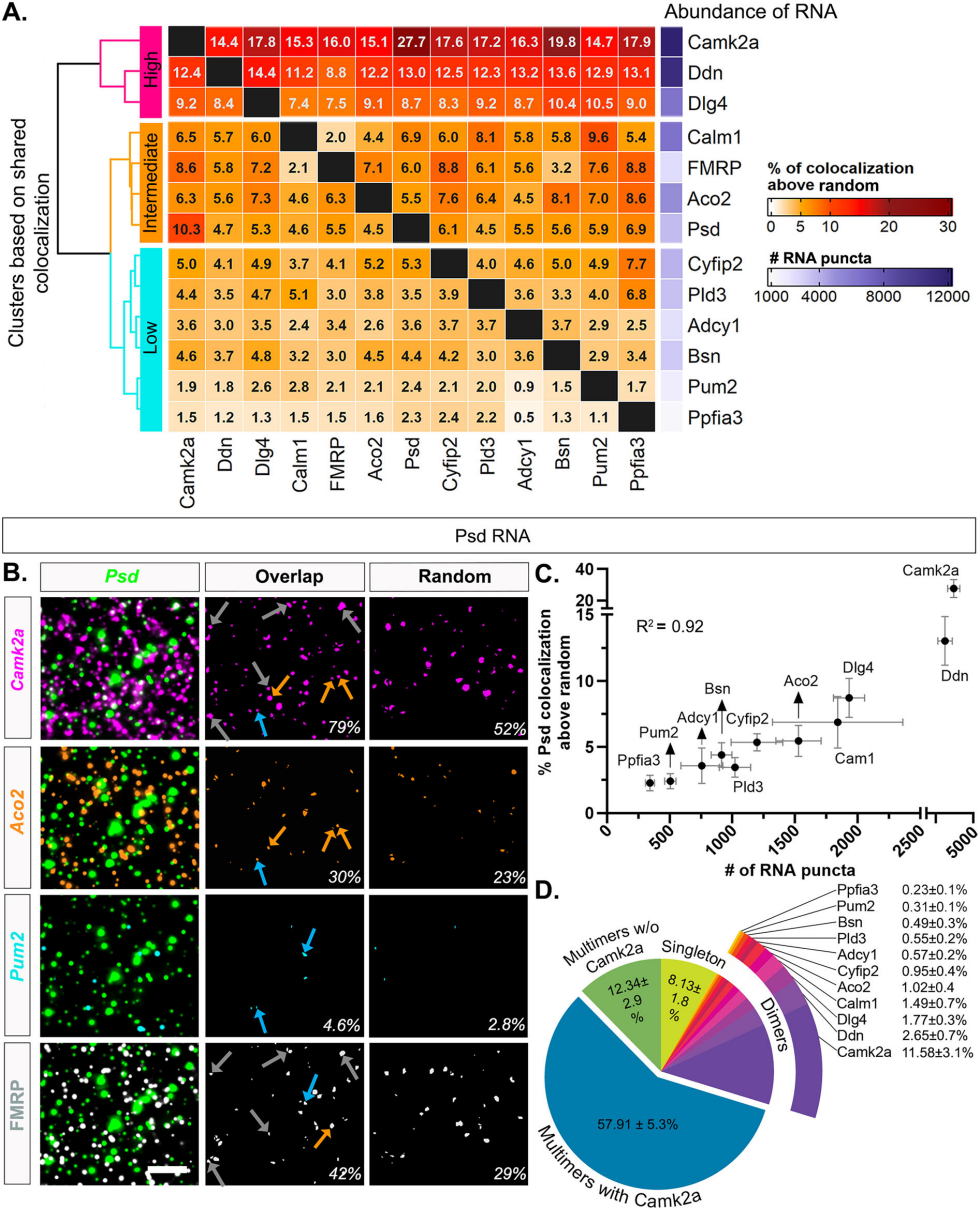
mRNA pairwise colocalization patterns correlate with abundance. ***A***, Percentage of RNAs colocalized in pairwise combinations above random (averaged across *N* = 4 mice). Percentage was calculated by dividing the number of overlapping puncta by the total number of RNAs that correspond to each channel (columns), then subtracting the percentage obtained after rotating one of the images 90° (Extended Data Fig. 4-1). ***B***, Representative images of colocalization with *Psd*. The first column consists of merged images of *Psd* (green,) with (***i***) *Camk2a* (magenta), (***ii***) *Aco2* (orange), (***iii***) *Pum2* (cyan), and (***iv***) FMRP protein (white). The middle column shows the intersecting area of each pair of RNAs *Psd*/*Camk2a* (magenta, 85 out of 107 or 79% of *Psd* RNA colocalize with *Camk2a* in this image), *Psd*/*Aco2* (orange, 33/107, 30%), *Psd*/*Pum2* (cyan, 5/107, 4.6%), and *Psd*/FMRP (white, 45/107, 42%). The third column shows the intersect of *Psd* colocalized at random (rotated 90°; *Psd*/*Camk2a* = 56/107, 52%; *Psd*/*Aco2* = 25/107, 23%; *Psd*/*Pum2* = 3/107, 2.8%; *Psd*/FMRP = 32/107, 29%). Scale bar, 5 µm. ***C***, Correlation plot of % *Psd* colocalized with the other 11 mRNAs after subtraction of random colocalization and their abundance (*R*^2^ = 0.92). ***D***, Pie chart of *Psd* mRNA puncta compositions. The data shown here are averaged from four 52 × 52 µm^2^ ROIs per animal then averaged across *N* = 4 mice and presented as mean ± SEM. 91.86 ± 1.8% (vs 65.1 ± 5.4% random in Extended Data Fig. 4-4) of *Psd* RNA have overlapping puncta from all other mRNA channels combined, i.e., colocalized mRNAs that include dimers (only one other mRNA) and multimers (at least two other mRNAs). The percentage of colocalized *Psd* dimers is almost at a level that was observed at random for each pair, except for *Camk2a*, where the colocalization is higher than random (11.58 ± 3.1% of *Psd-Camk2a* dimers vs random 6.68 ± 1.1%). 57 ± 5.3% of *Psd* mRNA puncta are multimers that have *Camk2a* and at least one other colocalized mRNA, which is higher than random (34.89 ± 4.9%). 12.34 ± 2.9% of *Psd* mRNA are also multimers (vs random 8.29 ± 1.6%) but do not have *Camk2a*. Lastly, 8.13 ± 1.8% of *Psd* mRNA are not colocalized with any of the other mRNAs in our dataset, which is noticeably lower than observed at random (34.9 ± 5.4%; *N* = 4 mice; Refer to Extended Data Figs. 4-1–4-6 for more details).

10.1523/ENEURO.0184-25.2025.f3-1 Figure 3-1 **(Refers to Figure 3 & 4) Schematic showing workflow of HiPlex smFISH**. Download Figure 3-1, TIF file.

10.1523/ENEURO.0184-25.2025.f3-2 Figure 3-2 **(Refers to Figure 3 & 4) HiPlex image processing and segmentation.** Raw and processed negative control images probed for the bacterial RNA *DapB* in each channel. Negative control images were acquired with identical acquisition parameters as experimental images shown below from all three rounds of HiPlex smFISH. Experimental images are presented with the same intensity thresholds as the corresponding negative control channels. The last row displays segmented binary layers for the *Psd, Camk2a*, and *Ppfia3* channels, created using intensity thresholds determined from the negative control image of the corresponding channels in each round. Scale: 5 µm. Download Figure 3-2, TIF file.

10.1523/ENEURO.0184-25.2025.f3-3 Figure 3-3 **(Refers to Figure 3 & 4) Abundance of each mRNA in the CA2 neuropil.** Each symbol represents data from a biological replicate (N=4 mice). Error bars indicate SEM. Download Figure 3-3, TIF file.

10.1523/ENEURO.0184-25.2025.f3-4 Figure 3-4 **(Refers to Figure 3): mRNA puncta intensity distributions mirror mRNA puncta area distributions** **A.** Correlation plots of individual mRNA puncta area and raw total intensity from representative candidate mRNAs from each cluster from a representative mouse (small: *Calm1*, small broad: *Psd*, large broad: *Adcy1*, large: *Dlg4*). **B.** Same as (**A**) but with puncta total intensity normalized by mean intensity. **C.** Heatmap of relative % distribution of normalized puncta total intensity for 12 mRNA channels (Ave +/- SEM, N = 2 mice). Hierarchical clustering of the intensity % distributions revealed the same 4 clusters as the puncta area distributions. Download Figure 3-4, TIF file.

**Table 2.** Average median fluorescent puncta area and normalized total puncta intensity of the HiPlex mRNAs by size cluster (refers to Fig. 3)

Cluster based on size^a^	RNA	Average median puncta area (µm^2^)	Peak relative % area bin size (% of RNA ± SEM)	Peak relative % normalized intensity bin size a.u. (% of RNA ± SEM)	% of RNA (±SEM) within area bin (0.6–1.0 µm^2^)	% of RNA (±SEM) with area bin >1 µm^2^
Small	*Calm1*	0.20 ± 0.03	0.20 µm (27.02 ± 2.8%)	20 (30.88 ± 3.5%)	9.29 ± 1.46%	2.50 ± 0.64%
	*Ddn*	0.24 ± 0.02	0.20 µm (23.07 ± 1.8%)	20 (29.89 ± 3.4%)		
	*Pld3*	0.25 ± 0.03	0.20 µm (20.98 ± 1.4%)	10 (27.47 ± 8.7%)		
Small broad	*Camk2a*	0.24 ± 0.02	0.10 µm (21.93 ± 0.8%)	10 (22.51 ± 2.04%)	15.89 ± 1.71%	9.38 ± 1.34%
	*Psd*	0.30 ± 0.05	0.10 µm (20.48 ± 2.1%)	10 (21.78 ± 9.02%)		
Large broad	*Adcy1*	0.25 ± 0.01	0.20 µm (18.47 ± %)	20 (20.24 ± 0.9%)	14.47 ± 1.21%	2.69 ± 0.60%
	*Bsn*	0.32 ± 0.03	0.30 µm (20.88 ± 1.1%)	30 (20.71 ± 2.9%)		
	*Cyfip2*	0.29 ± 0.02	0.30 µm (20.90 ± 0.8%)	20 (21.27 ± 1.9%)		
	*Aco2*	0.28 ± 0.01	0.30 µm (22.33 ± 0.7%)	20 (26.95 ± 6.2%)		
Large	*Ppfia3*	0.34 ± 0.01	0.30 µm (20.88 ± 0.6%)	40 (19.64 ± 4.1%)	16.32 ± 1.49%	3.14 ± 0.26%
	*Pum2*	0.34 ± 0.02	0.30 µm (20.62 ± 1.4%)	30 (23.15 ± 2.1%)		
	*Dlg4*	0.36 ± 0.02	0.30 µm (19.50 ± 2.1%)	30 (23.67 ± 1.3%)		

Puncta area data are from *N* = 4 mice and intensity data from *N* = 2 mice.

a Clusters were determined via hierarchical clustering of the relative % area distributions.

Unsupervised hierarchical clustering analysis of the mRNA puncta area distributions (see Materials and Methods) was used to determine mRNAs with similar area distribution patterns (Fig. 3*C*,*D*; Table 2). Based on manhattan distances, the dendrogram revealed four distinct distribution patterns that segregate primarily based on peak relative % area and the shape of the area distribution (narrow vs broad; Fig. 3*C*). The first cluster is composed of mRNAs with consistently “small” mRNA puncta (*Ddn*, *Pld3*, and *Calm1*, labeled magenta in the dendrogram; Fig. 3*C*), whereby, on average, ∼55% of mRNA puncta are <0.3 µm^2^ (54.57 ± 4.84%, averaged across mRNAs in this cluster from *N* = 4 mice) with the largest relative % peak (23.69 ± 1.77%) at 0.2 µm^2^. These consistently small mRNAs have fewer than 10% puncta (9.29 ± 1.46%) sized 0.6–1.0 µm^2^ and only 3% (2.50 ± 0.64%) of puncta larger than 1.0 µm^2^. In contrast, the fourth cluster is composed of mRNAs with consistently “large” mRNA puncta (*Dlg4*, *Pum2*, and *Ppfia3*; Fig. 3*C*, labeled blue) whereby, on average, ∼50% of the puncta are 0.3–0.5 µm^2^ (49.80 ± 1.16%) with the largest relative peak (20.33 ± 0.42%) at 0.3 µm^2^. These consistently large mRNAs have <5% puncta larger than 1.0 µm^2^ (3.14 ± 0.26%). There are two intermediary clusters, “small broad” (*Camk2a* and *Psd*; Fig. 3*C*, labeled light green) and “large broad” (*Adcy1*, *Bsn*, *Aco2*, and *Cyfip2*; Fig. 3*C*, labeled dark green) that have relatively broader mRNA puncta area distributions that segregate with either the “small” or “large clusters,” respectively. The “small broad” cluster (*Camk2a* and *Psd*) shows a very broad distribution with the largest relative peak (21.21 ± 0.72%) equal to or less than 0.2 µm^2^ and a larger population of mRNA puncta sized 0.6–1.0 µm^2^ that accounts for >15% (15.89 ± 1.71%). These “small broad” mRNAs have the largest fraction of mRNA puncta >1.0 µm^2^ at nearly 10% (9.38 ± 1.34%). The “large broad” cluster (*Adcy1*, *Bsn*, *Aco2*, and *Cyfip2*) also shows a broad distribution with the largest relative peak (38.85 ± 1.12%) between 0.2 and 0.3 µm^2^ and a larger population of mRNA puncta sized 0.6–1.0 µm^2^ that accounts for ∼15% (14.47 ± 1.21%). However, these “large broad” mRNAs have only ∼3% of puncta larger than 1.0 µm^2^ (2.69 ± 0.60%). Small and large populations are denoted on the representative images with magenta and blue arrows, respectively (Fig. 3*D*).

It is interesting to note that even some of the most abundant neuropil mRNAs visualized here contain populations with consistently small RNA puncta areas (i.e., *Ddn*, *Calm1*). These data suggest that mRNAs, regardless of abundance, vary considerably in RNA puncta area, both within a transcript population and across different transcripts. Consistent with the *Arc* probe dilution results, we interpret larger RNA puncta areas to likely represent RNA complexes with multiple copies of the same transcript, whereas the smaller RNAs likely represent RNA complexes containing fewer copies of transcripts or perhaps a single copy. To confirm that larger mRNA puncta have higher fluorescence intensity values, we correlated individual puncta areas with raw and normalized puncta total intensity values on a subset of the data. We found strong correlations for each mRNA channel. Representative examples are shown from each puncta area-defined cluster (Extended Data Fig. 3-4*A*,*B*). When we plotted the average relative % normalized total intensity per puncta for each mRNA and hierarchically clustered the data as above, we observed the same four clusters as revealed by clustering the puncta area distribution data (Extended Data Fig. 3-4*C*).

### Putative FMRP-target mRNAs colocalize in the neuropil based on abundance

To systematically characterize whether any particular FMRP-target mRNAs display similar colocalization profiles (and thus suggestive of coregulation), we measured the number of overlapping fluorescent puncta between two mRNA channels (at least 1 pixel overlap with of the reference RNA puncta) to determine pairwise colocalization values (66 pairwise combinations; Carson et al., 2008; Gao et al., 2008; Batish et al., 2012). For each pair across the 12 mRNAs, we expressed the colocalization values as a percentage of each individual mRNA (Fig. 4, Extended Data Fig. 4-1) and as a percentage of the combined pair (Extended Data Fig. 4-2). We reasoned that, when the number of colocalized molecules are expressed as a fraction of one RNA for each of the other 11 RNAs, this would reveal if an RNA species has a tendency of clustering more as a pair with one species versus others. In addition, when the number of colocalized molecules is expressed as a fraction of both mRNAs, it controls for differences in abundance across RNA pairs (Batish et al., 2012). We included well-characterized neuropil localized RNAs (*Camk2a*, *Dlg4* also known as *Psd95*, *Cyfip2*, *Ddn*) and uncharacterized mRNAs (*Aco2*, *Psd*, *Pld3*). The degree of colocalization across pairs in properly registered experimental images ranged from 4.21 ± 0.48% (*Adcy1*/*Ppfia3*) to 71.38 ± 4.91% (*Camk2a*/*Ppfia3*; Extended Data Fig. 4-1*A*). The degree of colocalization that was observed at random (one image from every pair rotated 90°, which controls for the differences in expression across mRNA pairs) ranged from 3.07 ± 0.41% (*Aco2*/*Ppfia3*) to 53.74 ± 4.75% (*Pum2*/*Camk2a*; Extended Data Fig. 4-1*B*). We then subtracted the random colocalization percentage from the percentage obtained from the properly registered experimental images, anticipating that random colocalization subtraction would eliminate the relationship with abundance, and visualized the result as a heatmap (Fig. 4*A*). The range of colocalization percentages above random spanned from 0.53 ± 0.36% (*Adcy1*/*Ppfia3*) to 27.74 ± 5.70% (*Psd*/*Camk2a*). Thus, after correcting for random colocalization, some mRNAs were rarely colocalized whereas others showed ∼20–30 times more colocalization, suggesting a difference in the propensity of mRNA species to be colocalized.

10.1523/ENEURO.0184-25.2025.f4-1 Figure 4-1 **(Refers to Figure 4A) Heatmaps showing the total average pairwise colocalization of mRNAs in properly registered (experimental) images (A) and in rotated (random) images (B).** The percentage values in each column are calculated by dividing the number of column mRNAs colocalizing with each row mRNA by the total number of column mRNAs, i.e. 5.2% of *Ddn* colocalizes with *Ppfia3*, whereas 54.4% of *Ppfia3* colocalizes with *Ddn* before random colocalization subtraction (see Figure 4A). Values are the average of N=4 mice (four 52 X 52 µm^2^ images averaged per mouse). Download Figure 4-1, TIF file.

10.1523/ENEURO.0184-25.2025.f4-2 Figure 4-2 **(Refers to Figure 4) Pairwise colocalization of neuropil localized mRNAs analyzed as in Batish et al.** For each pair of comparisons, the number of overlapping mRNA puncta between two channels was divided by the combined count of the two mRNAs being compared and expressed as a percentage (average of N=4 mice). Hierarchical clustering of the data revealed a very similar pattern (as shown in Figure 4A) showing that every mRNA is colocalized more with highly abundant mRNAs (*Camk2a, Ddn, Dlg4*) and show fewer instances of colocalization with mRNAs that are of lower abundance (*Pum2, Ppfia3*). mRNAs in intermediary clusters also show a similar trend although their specific orders are more variable compared to Figure 4A. Download Figure 4-2, TIF file.

10.1523/ENEURO.0184-25.2025.f4-3 Figure 4-3 **(Refers to Figure 4C) The positive correlation between pairwise colocalization and mRNA abundance exists regardless of expression** *Ddn* **(A)** and *Pum2* **(B)** exhibit high and low abundance, respectively, in CA2 neuropil. However, these mRNAs display a consistent positive correlation between % colocalization (random colocalization subtracted) and the abundance of the 11 paired mRNAs (*Ddn* R^2^ = 0.98 and *Pum2* R^2^ = 0.95). (N=4 mice. Error bars indicate SEM.) Download Figure 4-3, TIF file.

10.1523/ENEURO.0184-25.2025.f4-4 Figure 4-4 **(Refers to Figure 4D) Pie chart of *Psd* mRNA composition that was observed due to random overlap of mRNA fluorescent puncta** *Psd* image was rotated 90 degrees and colocalization of *Psd* with other eleven mRNAs combined were quantified and averaged from the same four 52X52 µm^2^ ROIs per animal as done for the registered experimental images. Individual animal averages were then averaged across N=4 mice and presented here as mean ± SEM. 65.1 ± 5.4% of *Psd* mRNA puncta (vs. 91.86 ± 1.8% in properly registered images) overlap randomly with at least one other mRNA that include dimers (*Psd* with only one other mRNA) or multimers (*Psd* with at least two other mRNAs). Consistent with the pairwise colocalization data where the extent of colocalization scales with mRNA abundance, the percentage of randomly colocalized dimers increases as mRNA abundance increases. However, the percentage of random dimers is equal to or greater than the percentage of dimers from properly registered images, with the exception of *Psd/Camk2a* dimers that are present at lower percentage than experimental (random *Psd/Camk2a* dimers 6.68 ± 1.1% versus properly registered *Psd/Camk2a* dimers 11.58 ± 3.1%). Random *Psd*-multimers with *Camk2a* (34.9 ± 5.0%) and without *Camk2a* (8.29 ± 1.6%) are appreciably lower than experimental images (57.91 ± 5.3% and 12.34 ± 2.9%, respectively), indicating multimer populations dominate colocalized Psd mRNA puncta compositions in our data. Download Figure 4-4, TIF file.

Next, we hierarchically clustered the shared colocalization patterns, which revealed three distinct clusters displaying consistently “high,” “intermediate,” or “low” levels of colocalization across all pairwise comparisons. Unexpectedly, levels of colocalization increased with mRNA abundance such that the three most abundant mRNAs in our dataset, *Camk2a*, *Ddn*, and *Dlg4* (# of RNA puncta: *Camk2a* = 12,829 ± 1,646; *Ddn* = 11,114 ± 1,262, *Dlg4* = 6,426 ± 424, *N* = 4 mice; Extended Data Fig. 3-3), exhibited uniformly high colocalization patterns with each of the other mRNAs. mRNAs with intermediate levels of abundance (*Calm1* = 6,451 ± 2,096, *Aco2* = 5,054 ± 450, *Psd* = 3,648 ± 764; *N* = 4 mice; Extended Data Fig. 3-3) consistently demonstrated intermediary levels of colocalization across all pairwise comparisons. mRNAs with relatively lower levels of abundance (*Pld3* = 3,457 ± 427, *Cyfip2* = 3,919 ± 725, *Adcy1* = 2,327 ± 407, *Bsn* = 3,157 ± 450, *Pum2* = 1,694 ± 208, *Ppfia3* = 1,162 ± 178, *N* = 4 mice; Extended Data Fig. 3-3) typically showed lower levels of colocalization across all pairwise comparisons.

Despite our lenient definition of overlap, which overestimates the occurrence of true heterotypic granules and results in a high level of “random” colocalization, it is possible that the effect of abundance was not fully controlled for. Therefore, we also expressed the pairwise colocalization values as a percentage of the sum of both mRNAs in the pair. Hierarchical clustering of these values was similarly scaled by mRNA abundance (Extended Data Fig. 4-2). The degree of colocalization ranged from 2.8% (*Adcy1*/*Ppfia3*) to 24.5% (*Camk2a*/*Ddn*). In both analyses, the most abundant mRNAs (*Camk2a*, *Ddn*, *Dlg4*) colocalized the most and the least abundant mRNAs (*Ppfia3*, *Pum2*) colocalized the least across all pairwise comparisons. The intermediary expressors were more variable in their specific order but followed a similar trend. Representative images of high (*Camk2a*), intermediate (*Aco2*), and low (*Pum2*) levels of colocalization with *Psd* mRNA are shown in Figure 4*B*, including the intersecting pixel overlaps for properly registered “experimental” and rotated “random” images. We chose *Psd* as an example mRNA due to its intermediate level of abundance, high % colocalization with *Camk2a*, and its broad fluorescent puncta area distribution presumably reflective of multiple populations of homotypic RNA particles. To visualize the influence of mRNA abundance on colocalization with the other 11 mRNAs, we correlated average % *Psd* colocalization above random versus average abundance (Fig. 4*C*). We found that the abundance of the mRNAs is highly correlated with the % *Psd* colocalization (*R*^2^ = 0.92). We observed the same pattern regardless of the expression of the mRNA that is being colocalized to, as *Ddn* (high expressor) and *Pum2* (low expressor) colocalization values were also highly correlated with abundance (*Ddn R*^2^ = 0.98, *Pum2 R*^2^ = 0.95; Extended Data Fig. 4-3).

### *Psd* mRNA puncta multimeric composition stratifies by mRNA abundance

Considering the technical limitation that pairwise colocalization values cannot portray the colocalization of multiple (more than two) RNAs, we then quantified the percentage of *Psd* mRNA that are localized in association with at least one (dimers) or more mRNA species (multimers) in properly registered experimental and rotated random images (Fig. 4*D*, Extended Data Fig. 4-4). Because the sum of measured pairwise colocalization of *Psd* mRNA (Extended Data Fig. 4-1*A*) exceeded 100%, it implied that a percentage of *Psd* mRNA was multimers (more than two mRNAs colocalized). The pie chart of *Psd* mRNA compositions from experimental images shows that 8.13 ± 1.8% of *Psd* mRNAs are not colocalized with any mRNA in our dataset (singleton, Fig. 4*D*), which is lower than observed for the 90° rotated random image (34.9 ± 5.4%; Extended Data Fig. 4-4). This suggests that a large fraction of the *Psd* mRNAs are heterotypic mRNA puncta, containing different types of mRNA transcripts, including *Psd* dimers, which have *Psd* colocalized with only one other type of transcript (measured here) and *Psd* multimers which contain more than two different transcripts including *Psd* (Fig. 4*D*, Extended Data Fig. 4-4). The percentage of *Psd* dimers range from 0.23 ± 0.1% (*Psd*/*Ppfia3*) to 11.58 ± 3.1% (*Psd*/*Camk2a*) which mirrored the abundance of the mRNAs. However, only the percentage of *Psd*/*Camk2a* dimers (11.58 ± 3.1%) is higher than random (6.68 ± 1.1%); all other *Psd* dimers are near random levels. We observed that 70.25 ± 4.8% of *Psd* mRNAs have at least three or more (including *Psd*) transcripts (multimers) of which the greatest fraction has *Camk2a* (57.91 ± 5.3%). Only 12.34 ± 2.9% of multimer *Psd* RNAs were without *Camk2a*, underscoring the dominating presence of *Camk2a* in both *Psd* dimers and multimers in our data. In contrast, the percentage of *Psd* multimers with (34.89 ± 4.9%) and without *Camk2a* (8.29 ± 1.6%) are lower in the rotated random images. Furthermore, when other mRNAs were quantified similarly, we found that an average of 88.6 ± 0.73% of each neuropil localized mRNA is localized with at least one other mRNA in our dataset compared with 65.62 ± 0.66% observed at random (all 12 comparisons are significantly higher than random, multiple unpaired two sample Welch's *t* tests with FDR correction; Extended Data Fig. 4-5). Only ∼11.40 ± 0.73% of the mRNAs are not colocalized with any of the other 11 mRNAs in this dataset. To confirm that the anatomical orientation of the neuropil has no effect on the calculation of random colocalization, we also rotated each mRNA image 180° to calculate the percentage of random overlapping puncta and did not observe any noticeable difference from 90° (Extended Data Fig. 4-5). However, we note the important caveat that there are assumed anatomical constraints (areas unavailable for colocalization) that are missing in the rotated image comparison that make these colocalization values an underestimate.

**Figure 5.**
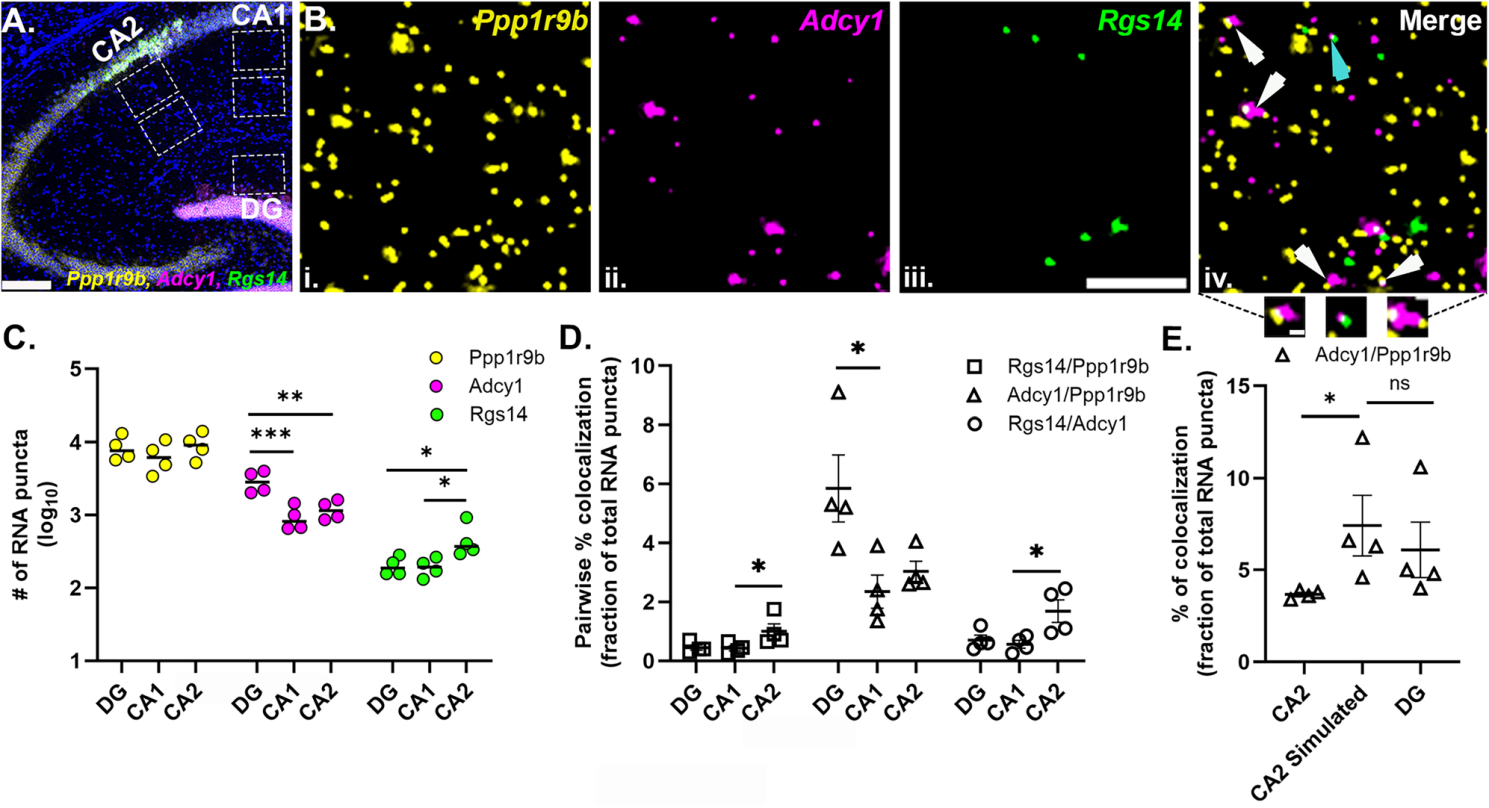
Neuropil mRNA colocalization patterns scale with mRNA abundance across cell types with varied mRNA expression. ***A***, Representative tilescan image of *Ppp1r9b* (yellow), *Adcy1* (magenta), and *Rgs14* (green) mRNAs and nuclei (blue) in adult mouse hippocampus. Dashed white boxes are regions analyzed from CA1 and CA2 proximal and distal dendrites and DG. ***B***, High-magnification representative images of (***i***) *Ppp1r9b*, (***ii***) *Adcy1*, (***iii***) *Rgs14*, and (***iv***) merged in CA2 distal dendrites. Arrows indicate colocalization of *Adcy1* with either *Ppp1r9b* (white) or *Rgs14* (cyan). Three example images of RNA colocalization in the callout section. ***C***, Quantification of the number of *Ppp1r9b*, *Adcy1*, and *Rgs14* RNAs in DG, CA1, and CA2 (# of *Ppp1r9b* mRNA in DG: 8,579 ± 1,685, CA1: 6,679 ± 1,619, CA2: 9,409 ± 1,881, RM ANOVA: *F* = 4.566, *p* = 0.132; # of *Adcy1* mRNA in DG: 2,969 ± 501, CA1: 943 ± 187, CA2: 1,202 ± 180, RM ANOVA: *F* = 108.3, *p* = 0.0007; # of *Rgs14* mRNA in DG: 204 ± 30, CA1: 196 ± 28, CA2: 490 ± 145, RM ANOVA: *F* = 21.36, *p* = 0.0030). Stats were run on the transformed (log_10_) values as plotted to meet the normality assumption. Tukey's post hoc tests reported on the plot. ***D***, Pairwise % colocalization of *Rgs14*/*Ppp1r9b*, *Adcy1*/*Ppp1r9b*, and *Adcy1*/*Rgs14* expressed as a percentage fraction of total number of RNAs in the pair. Some comparisons failed the Shapiro–Wilk normality test, so a Friedman rank-based ANOVA was run. *Adcy1*/*Ppp1r9b* Friedman statistic = 8.00, *p* = 0.0046, *Adcy1*/*Rgs14* Friedman statistic = 6.50, *p* = 0.041, *Rgs14*/*Ppp1r9b* Friedman statistic = 6.50, *p* = 0.041, Dunn's post hoc test reported on the plot. ***E***, *Adcy1*/*Ppp1r9b* pairwise colocalization in experimental CA2 and DG images compared with simulated CA2 images where *Adcy1* RNA puncta were randomly added to make CA2 equivalent to DG. Data failed the Shapiro–Wilk normality test, so Friedman rank-based ANOVA was run. Friedman statistic = 8.00, *p* = 0.0046. Dunn's post hoc test reported on the plot. *N* = 4 mice. Error bars indicate SEM. *p < 0.05, **p < 0.01, ***p < 0.001. Scale bars: (***A***) 200 µm, (***B***) 5 µm, 0.5 µm (refer to Extended Data Figs. 5-1, 5-2 for more details).

10.1523/ENEURO.0184-25.2025.f4-5 Figure 4-5 **(Refers to Figure 4D) The majority of neuropil localized mRNAs spatially interact with at least one other mRNA** Total % colocalization of each mRNA with any of the other 11 mRNAs from properly registered experimental images and 90 degree as well as 180-degree rotated images. Each symbol represents mean ± SEM from four biological replicates. % colocalization from experimental images were significantly higher compared to that in 90-degree rotated images (multiple unpaired two sample Welch’s t-test with FDR correction, p<0.01 for every pair) and 180-degree rotated images (multiple unpaired two sample Welch’s t-test with FDR correction, p<0.01 for every pair). N=4 mice. Error bars indicate SEM. Download Figure 4-5, TIF file.

To test whether association with FMRP influences the relationship of pairwise colocalization or heterotypic RNP associations, we subsetted the dataset by only selecting the mRNA puncta from each channel that colocalized with FMRP protein (Extended Data Fig. 4-6). Our hypothesis was that the observed colocalization after random subtraction would be higher in the presence of FMRP indicating that having a shared multivalent transacting protein makes mRNAs more likely to multimerize with each other into common RNPs. In properly registered experimental images, the degree of pairwise mRNA–mRNA colocalization in FMRP-containing RNPs ranged from 2.8% (*Pld3*/*Ppfia3*) to 60.3% (*Psd*/*Camk2a*; Extended Data Fig. 4-6*A*i). We then, as mentioned above, calculated pairwise colocalization by rotating one image from each mRNA pair to 90° (Extended Data Fig. 4-6*A*ii). Random pairwise colocalization ranged from 0.3% (*Adcy1*/*Ppfia3*) to 16.6% (*Adcy1*/*Camk2a*). After random colocalization was subtracted from the experimental colocalization, the percentage of pairwise colocalization ranged from 1.8% (*Pld3*/*Ppfia3*) to 47.4% (*Psd*/*Camk2a*), revealing a similar trend of pairwise colocalization that tracks with mRNA abundance (Extended Data Fig. 4-6*B*). Although the general trend was similar, we saw an overall gain of % colocalization for each pair in the FMRP-containing RNPs compared with the computation including all RNAs as shown by the correlation plot comparing *Psd* pairwise % colocalization with and without FMRP (Extended Data Fig. 4-6*C*).

10.1523/ENEURO.0184-25.2025.f4-6 Figure 4-6 **(Refers to Figure 4) Colocalization of mRNAs within FMRP-containing RNPs.** **A.** Heatmaps of experimental (Ai. left) and random (Aii. right, 90 degree rotated) % colocalization of each mRNA pair within FMRP containing RNP. Percentage was calculated by dividing the number of overlapping puncta by the total number of the column mRNA puncta. **B.** Correlation plot of the % FMRP colocalized with each mRNA (random colocalization subtracted) and mRNA abundance (R^2^ = 0.94). **C.** Correlation plot of pairwise *Psd* colocalization percentage with other RNAs within all neuropil RNA puncta (X-axis) and FMRP-containing RNPs (Y-axis). **D.** FMRP-containing *Psd* RNP compositions in experimental images. **E.** FMRP-containing *Psd* RNP compositions in random 90-degree rotated images. (N=2 mice). Download Figure 4-6, TIF file.

We then quantified the percentage of FMRP-containing *Psd* mRNAs that are colocalized with at least one (dimer FMRP cargo) or more species of mRNAs (multimer FMRP cargo) from both properly registered experimental and 90° rotated random images. These data are plotted as experimental and random *Psd* RNP composition pie charts (Extended Data Fig. 4-6*D*,*E*). As we noted previously for all mRNAs detected, the majority of FMRP-containing *Psd* mRNAs (86.4%) are colocalized with at least one other mRNA along with FMRP protein, which is higher than random (25.3%). The difference in experimental versus random colocalization (∼3.3-fold) is greater than reported for analyses without FMRP (∼1.3-fold; Extended Data Fig. 4-4), driven by a decrease in the random but not experimental values. Of these *Psd*-FMRP cargoes, the percentage of *Psd* having only one other mRNA (*Psd* dimers) is at the level of random colocalization except for *Psd*-*Camk2a* (22.6% vs random 5.1%). A total of 41.06% *Psd*-FMRP cargoes are multimers with *Camk2a* mRNA as opposed to only 7.8% observed at random, indicating the predominance of *Camk2a* in both *Psd*-FMRP dimers and *Psd*-FMRP multimers. Then, 8.8% of *Psd*-FMRP cargoes are also multimers, higher than random (3.8%), that have three or more mRNAs without *Camk2a*. Lastly, 13.6% of FMRP-containing *Psd* RNPs are segregated from the other 11 mRNAs, which is remarkably lower than observed at random (74.6%). These data suggest that for these 12 neuropil localized mRNAs, when associated with FMRP, have a higher likelihood than random to multimerize with each other, with a bias for the highly abundant ones.

### Variation in neuropil mRNA abundance is sufficient to scale mRNA colocalization across cell types

To further explore the relationship of mRNA colocalization with mRNA abundance, we examined whether differences in the abundance of one mRNA within a pair (across different cell types) influenced the colocalization patterns of that mRNA. We performed 3Plex smFISH for *Rgs14*, *Adcy1*, and *Ppp1r9b* which are known hippocampal dendritic mRNAs (Farris et al., 2019) downstream of group I metabotropic glutamate receptor (mGluR1/5) Gq-mediated signaling (Wang et al., 2007, 2008; Wang and Zhuo, 2012; Evans et al., 2018; Morris et al., 2023; Samadi et al., 2023). Consistent with previous observations, all three mRNAs localized throughout the neuropil, both proximal and distal layers, in CA2 and CA1 cell types as well as the molecular layer of dentate gyrus (DG; Fig. 5*A*,*B*). However, each mRNA has a distinct expression pattern reflected by differences in the number of mRNA puncta, which is consistent with previous RNAseq studies (Farris et al., 2019; Hale et al., 2021). mRNA puncta number varies from hundreds (*Rgs14*) to thousands (*Adcy1* and *Ppp1r9b*) of transcripts (Fig. 5*C*). When comparing the same sized region of neuropil, the number of *Ppp1r9b* mRNA was similar in CA2, CA1, and DG [# of *Ppp1r9b* DG: 8,579 ± 1,685, CA1: 6,679 ± 1,619, CA2: 9,409 ± 1,881, no effect of cell type, one-way repeated-measures analysis of variance (RM ANOVA): *F* = 4.566, *p* = 0.132, *N* = 4 mice]. *Rgs14* mRNA count was significantly higher in CA2 (# of *Rgs14* mRNA DG: 204 ± 30, CA1: 196 ± 28, CA2: 490 ± 145, significant effect of cell type, RM ANOVA: *F* = 21.36, *p* = 0.0030) than that in CA1 (*p* = 0.0306, Tukey's post hoc test) and DG (*p* = 0.0191, Tukey's post hoc test). *Adcy1* mRNA count was almost 2.5-fold higher in DG (# of *Adcy1* DG: 2,969 ± 501, CA1: 943 ± 187, CA2: 1,202 ± 180, significant effect of cell type, RM ANOVA: *F* = 108.3, *p* = 0.0007) than that in CA1 (*p* = 0.007, Tukey's post hoc test) and CA2 (*p* = 0.0018, Tukey's post hoc test).

10.1523/ENEURO.0184-25.2025.f5-1 Figure 5-1 **(Refers to Figure 5) 2D centroid-to-centroid distance based analysis reveals mRNA pairwise colocalization stratifies by abundance.** **A.** 3Plex mRNA pairwise colocalization is expressed as a percentage of the combined total mRNA puncta count in DG. The two more abundant mRNAs, *Adcy1* and *Ppp1r9b,* are colocalized (7.62 ± 1.45%) significantly more than *Adcy1/Rgs14* (0.89 ± 0.22%) and *Rgs14/Ppp1r9b* (0.57 ± 0.07%) when colocalization is defined as >1% overlap (overall effect of mRNA pair, RM ANOVA: F = 55.86, p = 0.0001, N=4 mice) or >50% overlap (overall effect of mRNA pair, RM ANOVA, F = 28.80, p = 0.0008, N=4 mice). Stats were run on the transformed (log_10_) values as plotted to meet the normality assumption. Tukey’s post hoc tests reported on the plot. **p<0.01; ***p<0.001 **B.** Heatmap showing mRNA puncta colocalized at >1% and >50% overlap plotted as a percentage of each mRNA (averaged across N=4 mice +/- SEM, “_50” represents >50% overlap). Data hierarchically cluster by RNA pair and stratify by abundance (purple shading). Download Figure 5-1, TIF file.

10.1523/ENEURO.0184-25.2025.f5-2 Figure 5-2 **(Refers to Figure 5) *Rgs14*, *Adcy1* and *Ppp1r9b* are variable in fluorescent puncta areas across mRNAs but not cell types.** Hierarchical clustering of relative percent area distribution of *Rgs14*, *Adcy1* and *Ppp1r9b* in CA2, CA1 and DG of adult mouse hippocampus. Median puncta areas were not significantly different across cell types for each mRNA although heterogeneity in area distribution across mRNAs was observed similar to the HiPlex data. *Rgs14* median puncta area CA1: 0.10 ± 0.01 µm^2^, CA2: 0.10 ± 0.01 µm^2^, DG: 0.10 ± 0.02 µm^2^ (no effect of cell-type, RM ANOVA, F = 0.026, p = 0.9744, N=4 mice). *Adcy1* median puncta area CA1: 0.22 ± 0.03 µm^2^, CA2: 0.22 ± 0.03 µm^2^, DG: 0.19 ± 0.02 µm^2^ (no effect of cell type, RM ANOVA: F = 0.5406, p = 0.6083, N=4 mice). *Ppp1r9b* median puncta area CA1: 0.22 ± 0.01 µm^2^, CA2: 0.24 ± 0.02 µm^2^, DG: 0.22 ± 0.03 µm^2^ (no-effect of cell type, RM ANOVA, F = 0.5507, p = 0.6032, N=4 mice). Since *Adcy1* and *Ppp1r9b* median puncta area data were more likely to be a lognormal distribution, we repeated the RM ANOVA on log_10_ transformed values, which did not change the statistical result. Download Figure 5-2, TIF file.

We then tested whether differences in mRNA expression influences mRNA pairwise colocalization values across these three cell types (Fig. 5*D*). We quantified the number of overlapping puncta (>1 pixel overlap) between two channels and expressed that number as a percentage of the total number of mRNA puncta in the pair (Fig. 5*D*). Based on our HiPlex data, we predicted that the *Adcy1*/*Ppp1r9b* mRNA pair would demonstrate higher colocalization compared with other pairs (*Adcy1*/*Rgs14*, *Rgs14*/*Ppp1r9b*) in all three cell types, and this effect would be significantly higher in DG compared with that in CA2 and CA1 due to the significantly higher number of *Adcy1* mRNA in DG. Indeed, we observed 5.85 ± 1.14% of total *Adcy1*/*Ppp1r9b* mRNA were colocalized in DG compared with 2.35 ± 0.56% in CA1 and 3.04 ± 0.34% in CA2 (significant effect of cell type, rank-based ANOVA, Friedman statistic: 8.00, *p* = 0.0046, *N* = 4 mice), and this difference was significant for DG versus CA1 (*p* = 0.0140, Dunn's multiple-comparison test). Similarly, we also predicted that *Adcy1*/*Rgs14* would be highly colocalized in DG compared with CA1 and CA2 due to the higher abundance of *Adcy1* in DG (significant effect of cell type, rank-based ANOVA, Friedman statistic: 6.500, *p* = 0.0417). Instead, *Adcy1*/*Rgs14* demonstrated higher colocalization in CA2 (1.69 ± 0.38%), which has the highest number of *Rgs14* mRNA among these three cell types, and this effect was significant compared with CA1 (colocalized *Adcy1*/*Rgs14* = 0.56 ± 0.13%, *p* = 0.0400, Dunn's post hoc test, *N* = 4 mice).

Acknowledging the limitations of our definition of colocalization, we next tested whether using a 2D centroid-based definition of colocalization (Batish et al., 2012; Eliscovich et al., 2017) would reproduce the effect of abundance on colocalization. For every puncta pair, a 2D centroid-based distance <50% of the sum of the radii (>50% overlap) was considered colocalized. A similar calculation was done to compare puncta colocalization with >1% overlap. Defining colocalization at >50% or >1% overlap did not change the relationship of mRNA colocalization with abundance (Extended Data Fig. 5-1). Two highly abundant mRNAs, *Adcy1* and *Ppp1r9b*, colocalized significantly more than *Rgs14*/*Adcy1* and *Rgs14*/*Ppp1r9b* pairs using both definitions of colocalization (Extended Data Fig. 5-1*A*). Moreover, when expressed as a % of mRNA colocalized, hierarchical clustering revealed that mRNA pairs clustered together regardless of % overlap indicating a similar influence of abundance on pairwise colocalization (Extended Data Fig. 5-1*B*). It should be noted that the more stringent definition of overlap decreased the number of colocalized mRNA puncta indicating that 1% overlap likely overestimates the true mRNA–mRNA colocalization within heterotypic RNPs.

We then tested whether simulating an increase in mRNA abundance would reproduce the expected increase in pairwise colocalization. Specifically, we randomly added *Adcy1* mRNA puncta to CA2 proximal dendrite images to make them have the equivalent number of *Adcy1* puncta as DG images taken from the same brain section, while keeping the number of other two mRNAs constant (# of *Adcy1* in DG: 2,973 ± 726, CA2 proximal: 1,248 ± 106, CA2 proximal simulated: 2,973 ± 726; # of *Ppp1r9b* in DG: 8,346 ± 1,449, CA2 proximal: 8,840 ± 1,698, CA2 simulated: 8,840 ± 1,698; # of *Rgs14* in DG: 235 ± 37, CA2 proximal: 622 ± 175, CA2 proximal simulated: 622 ± 175, *N* = 4 mice). The increased *Adcy1* mRNA in simulated CA2 resulted in a significant increase in pairwise % colocalization of *Adcy1*/*Ppp1r9b* mRNAs similar to experimental values in DG (% *Adcy1*/*Ppp1r9b* colocalized in CA2: 3.6 ± 0.11%, DG: 6.1 ± 1.51%, simulated CA2: 7.4 ± 1.65%; significant effect of cell type, Friedman statistic = 8.00, *p* = 0.0046, *N* = 4 mice; Dunn's post hoc tests: CA2 vs CA2 simulated: *p* = 0.0140, DG vs CA2 simulated: *p* = 0.4719; Fig. 5*E*). The slightly higher *Adcy1*/*Ppp1r9b* % colocalization in simulated CA2 compared with DG is likely due to the increase in *Ppp1r9b* mRNA puncta count in the chosen ROIs compared with DG. Thus, the most likely explanation of the observed colocalization is due to random overlaps driven by spatial proximity of mRNAs which in turn is dictated by mRNA abundance.

Lastly, building on our previous observation of fluorescent mRNA puncta area heterogeneity, we also quantified the fluorescent mRNA puncta area distributions of these mRNAs in CA1 and CA2 proximal and distal neuropil layers and DG. We found no differences between proximal and distal layers for puncta area, and therefore the data were collapsed and represented as one neuropil population per animal for CA2 and CA1. Across animals, we consistently observed the same pattern of puncta area heterogeneity for each mRNA species in all three hippocampal cell types (Extended Data Fig. 5-2). However, the distributions of mRNA puncta area were quite heterogeneous across mRNAs; in particular, *Rgs14* mRNA puncta were consistently small sized puncta in all three cell types with their largest relative % peak at 0.1 µm^2^ (68.5 ± 3.1%, averaged across mice for each cell type and then averaged across cell types). *Adcy1* and *Ppp1r9b*, however, showed a large broad distribution with the largest relative % peak at 0.20 µm^2^ (*Adcy1*: 34.2 ± 3.1%; *Ppp1r9b*: 27.9 ± 1.1%) and a qualitatively distinct larger population of mRNAs that are greater than 0.5 µm^2^ (*Adcy1*: 5.35 ± 2.04%; *Ppp1r9b*: 10.7 ± 1.2%). These large mRNA particles were not present for *Rgs14* mRNA (*Rgs14* median puncta area CA1: 0.10 ± 0.01 µm^2^, CA2: 0.10 ± 0.02 µm^2^, DG: 0.10 ± 0.02 µm^2^; no effect of cell type, RM ANOVA, *F* = 0.026, *p* = 0.9744, *N* = 4 mice; *Adcy1* mRNA median puncta area CA1: 0.22 ± 0.03 µm^2^, CA2: 0.22 ± 0.03 µm^2^, DG: 0.19 ± 0.02 µm^2^; no effect of cell type, RM ANOVA: *F* = 0.5406, *p* = 0.6083, *N* = 4 mice; *Ppp1r9b* median puncta area CA1: 0.22 ± 0.01 µm^2^, CA2: 0.24 ± 0.02 µm^2^, DG: 0.22 ± 0.03 µm^2^; no effect of cell type, RM ANOVA, *F* = 0.5507, *p* = 0.6032, *N* = 4 mice). Compared with the area distribution in our HiPlex data, the histogram of mRNA puncta area in this experiment was skewed smaller although the relative distinction between “small” and “large broad” clusters based on fluorescent puncta area were consistent. We compared the median puncta area of *Adcy1* mRNA in the CA2 distal neuropil images from this experiment with the HiPlex CA2 distal neuropil images and found no significant difference between *Adcy1* median puncta area between the two experiments (*Adcy1* median puncta area in CA2 distal neuropil measured in HiPlex smFISH: 0.25 ± 0.01 µm^2^, and 3Plex smFISH: 0.24 ± 0.04 µm^2^, two-tailed unpaired *t* test, *p* = 0.7304). These data suggest that these mRNAs can exist in consistently similar sized populations (both small and large homotypic mRNA complexes) across multiple cell types despite endogenous differences in their expression.

## Discussion

In this study, we visualized 15 neuropil localized mRNAs to investigate how mRNAs are spatially organized for delivery to synapses in intact rodent hippocampus. First, we provide evidence supporting the heterogeneity of neuropil mRNAs by describing differences in mRNA fluorescent puncta area and intensity. We interpret this data to reflect differences in the amount of individual mRNA transcripts per smFISH puncta. Second, by simultaneously visualizing a dozen neuropil localized FMRP-target mRNAs, we found that every mRNA we investigated, regardless of its abundance, colocalizes more with the highly abundant mRNAs compared with the lower abundance mRNAs. This result stands after correcting for random colocalization or the total fraction of the two mRNAs being compared. Our findings were similar for RNPs defined by the presence of FMRP. Third, the data suggests that mRNA colocalization correlates with mRNA abundance across multiple hippocampal cell types, an effect that can be recapitulated by simulations of mRNA abundance. Thus, we failed to identify selectivity in how these mRNAs associate with each other in the neuropil. Instead, the probability of these mRNAs spatially interacting within the neuropil is consistent with stochastic overlaps linked to mRNA neuropil abundance—a model predicted by mathematical modeling studies to be energetically cost efficient (Wagle et al., 2023; Bergmann et al., 2025). However, this conclusion is based on a rather lenient definition of colocalization (1 pixel spatial overlap in 2D between puncta). We did not investigate additional biological factors besides RNA abundance that can also potentially contribute to the association of these mRNAs into heterotypic FMRP granules. Therefore, further experiments are needed to evaluate the contribution of other plausible mechanisms involved in the selective association of RNAs in the neuropil.

### Localized mRNAs contain varying amounts of a single mRNA species

Neurons localize thousands of different mRNAs of variable abundance and subcellular distributions to support synaptic function. Yet, few studies have systematically characterized how mRNAs in the hippocampal neuropil are sorted into RNPs and how their compositions in vivo could support the delivery of thousands of mRNAs encoding proteins involved in many different biological processes. Mikl et al. (2011) investigated the localization of *Map2* and *Camk2a* mRNAs in hippocampal neurons in culture, showing that these mRNAs are present in dendrites in distinct RNPs, each containing as few as one or only a few copies of the same transcript with minimal colocalization between the two transcripts. Another study by Batish et al. (2012), visualized pairwise combinations of eight dendritically localized transcripts with smFISH in hippocampal cultured neurons, also showing unimodal distribution of mRNA puncta fluorescence intensities and ∼4% of colocalization between pairs of mRNAs, suggesting that mRNA molecules are trafficked singly and independently of others in neurons. In addition, there is evidence from in situ studies assessing individual mRNA content that supports the idea that mRNAs localize in variable copy number states. Single-molecule FISH detected β-actin mRNA in live hippocampal cultured neurons showed that RNPs may contain single as well as multiple copies of β-actin mRNA and the copy number decreased with increasing distance from the cell soma (Park et al., 2014). Variations in size and intensity of individual *Camk2a*, *Arc*, and neurogranin (*Ng*) RNA granules in developing neurons (fixed) were also reported by Gao et al. (2008). Further, a recent study by Donlin-Asp et al. (2021) used molecular beacons in cultured neurons to individually track endogenous mRNAs (*Camk2a* and *Psd95*) by live cell imaging. In addition to detecting single mRNA transport events, they observed mRNA–mRNA fusion events within the same transcript resulting in heterogeneous copy number states of mRNAs in neuronal dendrites. While extremely informative, most of these studies were done in primary neuronal cultures and limited in the number of species of localized mRNAs investigated, demonstrating a need to evaluate RNA copy number and composition for the growing list of localized mRNAs in intact neuronal circuits.

Our data on neuropil localized mRNA fluorescent puncta area and intensity in fixed rat and mouse hippocampus (DG, CA1, CA2) corroborate previous observations in culture that mRNA content varies from low copy number mRNAs to higher order homotypic mRNA clusters of the same transcript (multiple copies of the mRNA derived from the same gene). For the *Arc* dilution experiment, the decrease in *Arc* puncta intensity, diameter, and count suggest there are multiple pools of *Arc* puncta with different amounts of *Arc* mRNA. However, these data are relative to saturating probe conditions (1×), not to puncta with a known RNA copy number, so we cannot conclusively state that the loss of *Arc* puncta equates to a loss of puncta containing a single RNA. The differences in puncta area distributions across mRNAs were an unexpected finding that became obvious when visualizing a dozen mRNAs simultaneously in the same tissue section. However, without similar dilution calibration curves for each mRNA and/or fiduciary standards to control for different fluorophores and image acquisition parameters across mRNAs, we are not able to make strong claims about the significance of the observed differences. We did not observe any particular round of imaging or fluorophore wavelength to behave in a certain way that would explain the observed differences in puncta area distributions though. We also cannot exclude the possibility that the differences in fluorescent mRNA puncta intensity, diameter, or area could be due to differences in the number of probes bound to a target mRNA. However, because we compare puncta area and intensity distributions, which include all possible probe sizes/intensities, it is unlikely for different transcript probes (which all have 20 ZZ probe pairs) to result in different distributions (small, large, broad) unless there is something about the specific transcript (secondary structure, etc.) driving the bias. Further, it is difficult to imagine a technical explanation for observed puncta area and intensity differences within the same transcript, unless there is a biological mechanism restricting probe access to specific populations of transcripts that results in a non-Gaussian distribution of sizes. Lastly, we consistently observed that mRNA abundance did not exhibit any relation with its copy number states, regardless of whether we analyzed different mRNA transcripts with variable abundance or the same transcript with different levels of expression across cell types.

Our evidence in support of heterogeneous copy number of mRNAs (within and across 15 neuropil localized mRNAs) is consistent with the idea that multiple mRNA assembly states coexist for localizing at least these mRNAs, which may provide flexibility in regulating synaptic activity-induced changes in translation (Fernandez-Moya et al., 2014). Observations from non-neuronal systems, such as drosophila mRNA germ granules (Niepielko et al., 2018; Trcek et al., 2020), also indicate that localized mRNAs sort into homotypic clusters. However, with the limited number of visualized neuropil localized mRNAs, it is not yet clear whether the existence of distinct copy number states is a transcript-specific feature or a transcriptome-wide phenomenon. mRNA constituents of transporting or localized mRNA granules identified by synaptoneurosome or brain lysate fractionation are present in monosomes as well as in translationally silent stalled polysomes (Krichevsky and Kosik, 2001; Kanai et al., 2004; Hafner et al., 2019). Therefore, whether different sized mRNAs in our dataset reflect functional differences such as their association with other mRNAs and/or ribosomes or translational status is yet to be determined. Work on other types of cytoplasmic granules (p-bodies and stress granules) in living cell lines show that granule size correlates with increased granule stability (Moon et al., 2019). Further studies are needed to identify whether differences in mRNA puncta area/intensity (i.e., RNP size) and composition reflect different structural properties and/or functional mRNA states.

### mRNA colocalization within the neuropil scales with mRNA abundance

The composition of certain types of specialized mRNA granules (e.g., stress granules, germ granules) is influenced by mRNA abundance (Van Treeck et al., 2018; Trcek et al., 2020; Bauer et al., 2022). Experiments in *Drosophila* germ cell granules show that highly abundant mRNAs have higher seeding events to initiate homotypic RNP formations through self-recruitment and subsequently recruit other mRNAs to the RNPs (Niepielko et al., 2018). Data on localized neuronal mRNAs being influenced by mRNA abundance is comparatively limited. Bauer et al. visualized DDX6-positive mRNA granules in primary neurons in culture and showed that, independent of mRNA identity, assembly of these granules is facilitated by availability of free cytoplasmic mRNA levels and translational activity (Bauer et al., 2022). Wang and colleagues used barcode-based imaging method, MERFISH, combined with expansion microscopy to visualize 950 mRNA transcripts in neuronal culture (18 d in vitro) and showed that highly abundant mRNAs (*Camk2a*, *Ddn*, *Dlg4*, *Ppp1r9b*, *Shank1*, *Palm*) spatially cluster together in dendrites (Wang et al., 2020). Consistently, our pairwise colocalization data, using P17 mouse brain, shows that highly abundant neuropil mRNAs (*Camk2a*, *Ddn*, *Dlg4*) are spatially distributed as such that they colocalize the most with the other neuropil mRNAs in our dataset. This data suggests that the higher availability of these transcripts in spatial proximity to others can possibly promote clustering into RNPs with other mRNAs. However, there are several caveats to our approach that should be taken into consideration when interpreting the data.

First, our data relies on a lenient colocalization metric (1 pixel overlap) based on 2D overlapping in situ signals limited by 250 nm *x*–*y* resolution. This method presumably overestimates true levels of colocalization for heterotypic granules as noted by the high colocalization values from both experimental and random quantifications. Overestimating colocalization in this way may have masked underlying selectivity in granule composition. Indeed, when we analyzed the 3Plex data using lenient (>1% overlap) versus more stringent colocalization criteria (>50% overlap), we observed a reduced number of colocalization events. However, the stringently colocalized granules continued to stratify by abundance, validating our primary results. Nevertheless, additional super-resolution techniques (i.e., STORM) are required to prove whether any mRNAs investigated in this study are physically clustering within the same granule (<250 nm). Such methods, with higher sensitivity for detecting RNA–RNA interactions, could reveal selectivity within granules that was unable to be detected with the methods used here.

Second, the image rotation method for random subtraction does not fully recapitulate the cytoarchitecture (e.g., extracellular spaces, organelles, etc.) present in properly registered images that theoretically restricts the potential locations for overlap, which will result in an underestimation of random colocalization, thereby inflating the random-subtracted colocalization percentage observed. This is evident in the pairwise comparisons near random that are always above 0. To address this, we also plotted the data as a percentage of total mRNA puncta in the pair, as done previously (Tübing et al., 2010; Batish et al., 2012) to better account for the effect of mRNA abundance. This analysis showed that 24.5% of *Camk2a* and *Ddn* (two highest abundant in our dataset) are colocalized in CA2 neuropil whereas, only 3.5% of *Pum2* and *Ppfia3* (two least abundant in our dataset) are colocalized. Thus, the relationship between abundance and RNA colocalization is also evident in analyses that control for abundance substantiating our initial results.

Third, in contrast to the previous point, the image rotation method also results in some very high levels of random colocalization (>50%), in particular for the highly expressed mRNAs, calling into question the robustness of the metric and/or the resulting random-subtracted percentages. To account for this in the multitranscript colocalization analyses, where ∼65% of any mRNA randomly colocalized with at least one other mRNA, we subsetted the data to puncta defined by the presence of FMRP protein. The percent of FMRP-defined *Psd* puncta colocalized with any mRNA at random was considerably lower (25.3%) compared with all *Psd* puncta (65.1%). However, in experimental images, the percent of FMRP-defined *Psd* puncta colocalized with any mRNA (86.4%) was similar to the percent without FMRP (91.9%), indicating the robustness of assessing multi-RNA containing puncta compared with pairs. Within this subset of FMRP-defined *Psd* mRNA puncta, we also found that mRNA colocalization stratified by abundance. For example, FMRP-defined *Psd* puncta containing highly abundant *Camk2a* colocalized more (∼20% vs random 5%) than those containing lower abundant *Ppfia3* (0.6% vs random 0.3%). These data indicate that the high level of random colocalization was reduced when mRNA puncta were categorized as FMRP-defined RNPs, further validating our primary result.

Fourth, we only observed ∼40% colocalization when RNAscope probes were targeted against a single transcript. Thus, it is plausible that colocalization across transcripts may also be constrained by probe inaccessibility for tightly packed heterotypic granules. There are several alternative explanations that suggest otherwise. First, the samples are heavily digested with a protease. There is evidence that the proteolytic step before hybridization can increase mRNA detection efficiency by permeabilizing tissues after fixation and releasing RNAs from RBPs (Buxbaum et al., 2014; Young et al., 2020). Secondly, the RNAscope puncta counts are very similar to quantities reported from RNAseq datasets and FISH studies detecting the same transcripts but with different probe designs (Farris et al., 2019; Glock et al., 2021). This indicates that we can reliably detect the vast majority of neuropil localized mRNAs for each species, even when multiplexed, making it unlikely that we are missing (or not detecting) significant populations of mRNAs due to inaccessibility. Lastly, we were able to detect two mRNAs (*Psd*, *Camk2a*) and 1 granule marker protein (FMRP) spatially overlapping at levels higher than random. Even within this subset of FMRP-defined RNPs, we observed a similar relationship of mRNA colocalization with abundance that we identified earlier from visualizing the entire neuropil mRNA population. Therefore, if all heterotypic granules are similarly affected by inaccessibility, we assume that the relative differences in pairwise colocalization between these granules are an informed estimate of the underlying biological scenario.

### mRNA spatial distributions influence stochastic interactions

As proposed in the neuronal mRNA transport sushi-belt model, mRNAs patrol the neuronal processes in a multidirectional fashion with intermittent rest and run times and dynamic transient interactions (Song et al., 2018; Bauer et al., 2019; Ahn et al., 2023). Such a model would then predict, highly abundant mRNAs have a higher likelihood of random transient interactions during localization in a cell-autonomous fashion and the precision of sorting is obtained locally at the synapse level. Consistently, when we simulated higher mRNA abundance by increasing low *Adcy1* levels in CA2 to moderately high *Adcy1* levels as seen in DG, as predicted, it resulted in an increased % of *Adcy1*/*Ppp1r9b* mRNA colocalization. In the HiPlex data, the most abundant mRNA in our dataset, *Camk2a*, dominated in spatial overlaps with other mRNAs whether the puncta contained FMRP or not. *Camk2a* mRNA interacts with multiple other RBPs in addition to FMRP [RNG105 (Nakayama et al., n.d.; Shiina et al., 2005), CPEB (Wu et al., 1998), and Staufen (Ortiz et al., 2017)]. It seems reasonable to hypothesize that *Camk2a*-containing heterotypic RNPs might achieve some degree of mRNA selectivity based on coregulation with *Camk2a*-associated RBP(s). It is likely that RBPs influence RNP organization, through directly regulating mRNA abundance or through other mechanisms. However, we were unable to experimentally address this due to the technical limitations of staining for multiple RBPs with HiPlex.

We also did not determine whether other mRNA-specific features (such as sequence length, 5′ and 3′ UTR properties, RNA folding, translation efficiency, RBP interactions, etc.), may also be contributing to the observed high levels of colocalization for highly abundant mRNAs. It is important to investigate whether additional factors, such as the mRNA-specific features listed above, can also influence the association of different mRNA species into the heterotypic granules. Biophysical studies provide evidence that, in addition to mRNA concentration, mRNA sequence, structure, and stability influence in vitro mRNA–protein condensate formation (Boeynaems et al., 2019; Garcia-Jove Navarro et al., 2019; Roden and Gladfelter, 2021; Ripin and Parker, 2023). In addition to being highly abundant in the neuropil, *Camk2a*, *Ddn*, *Dlg4*, and *Ppp1r9b* transcripts are also highly translated in the dendrite as shown by their high ribosomal densities (Glock et al., 2020). *Camk2a* and *Dlg4* have also been predicted by in silico tools to strongly interact with FMRP (Cirillo et al., 2013). Thus, there are certain features specific to these mRNAs, other than their high expression, that may also contribute to their noticeable presence in the majority of neuropil localized heterotypic mRNA puncta in our data. Thus, further investigation into the sequence-specific features of the 15 neuropil localized mRNAs in our dataset is needed to determine whether and/or how those properties contribute to spatial overlaps of highly abundant FMRP-target and nontarget mRNAs in the neuropil. It is certainly possible that our findings of stochastic neuropil mRNA interactions and the relationship with mRNA abundance may not translate to RNPs composed of other mRNAs, or other FMRP-target RNAs, or RNPs defined by specific sets of RBPs that may confer specificity not detectable to the methods and analyses used here.

### Relevance to FMRP

There are multiple lines of evidence that show FMRP targets are differentially altered (or not) in the absence of FMRP at the level of mRNA localization (Steward et al., 1998; Miyashiro et al., 2003; Dictenberg et al., 2008). While it is beyond the scope of the current study, future perturbation experiments are required to assess whether mRNA colocalization is affected by the loss of FMRP. There is radioactive in situ hybridization evidence for a trending reduction in *Dlg4* mRNA in CA1 distal dendrites of adult Fmr1 knock-out (KO) mice (Zalfa et al., 2007). Several recent transcriptomic studies have also reported small, consistent decreases in FMRP-target mRNA abundance in Fmr1 KO mice hippocampus (Thomson et al., 2017; Sawicka et al., 2019; Hale et al., 2021). Combining these findings with our data, we expect that a lack of FMRP will decrease hippocampal neuropil mRNA abundance for a subset of these 12 mRNA targets and therefore, decrease mRNA colocalization patterns selectively for those affected mRNAs in relation to others. This would suggest that FMRP may regulate heterotypic RNP compositions through mRNA abundance, although other FMRP-mediated mechanisms may also contribute (Korb et al., 2017). Follow-up studies are needed to elucidate whether and how mRNA spatial colocalization patterns are influenced by RBPs or other factors that contribute to the localization and translation of messages at synapses.

## Synthesis

Reviewing Editor: Matthew Grubb, King's College London Decisions are customarily a result of the Reviewing Editor and the peer reviewers coming together and discussing their recommendations until a consensus is reached. When revisions are invited, a fact-based synthesis statement explaining their decision and outlining what is needed to prepare a revision will be listed below. The following reviewer(s) agreed to reveal their identity: Maria Vera.

Both Reviewers have positive views of the manuscript, and both also raise concerns that need to be addressed in order for the study's conclusions to be sufficiently supported. Please ensure that all of the individual comments below are fully addressed in any revised submission.

Reviewer 1

This study addresses an important unanswered question in the field of RNA localization in neurons about the nature of the RNA granules. The authors utilize an imaging approach of 12 synaptically localized mRNAs and utilize 2D co-localization as a metric for determining whether mRNAs associates with each other in same or different granules and identified new insights into the heterogeneity of dendritic RNA puncta in the hippocampus. They focused on transcripts that are FMRP targets and found that mRNAs are present in heterogenous copy number states and exhibit spatial clustering that is dependent on their abundance in the dendrites. While this is a descriptive study using 15 synaptically localized mRNAs, it performs extensive characterization on the distribution of different FMRP targets in the neuropil across three different hippocampal regions. Their conclusions are that the colocalization is guided by abundance only. However, further analysis and experiments are needed to prove that indeed stochastic interactions and not the RNA or RBP biology is the main driver for RNA granule organization. Here are some of the major comments:

1. The study assumes that overlapping signals represent true heterotypic granules, which may not be the case. The authors quantify overlapping puncta which are >1% overlap between two channels. Should it be at least >50% overlap, maybe even 75%. How did the authors choose 1%? Although they mention that the effect of RNA abundance remained the same with 50% overlap, one would expect that they would carry out the analysis with at least 50% overlap to begin with. Does the classification of the granules change once this overlapping criterion is used? Also, what is the 2D distance the 1% overlap refers to?

2. It seems that maximum extent of colocalization they can achieve with the smFISH RNAscope probes is approx. 30- 35%, and it is a technical limitation of the system. The fact that it happened for Shank2 and Arc mRNAs suggest that technical difficulties is accessing the transcripts effectively is a hurdle to achieving actual co-localization frequencies. For example, if two mRNAs are tightly packed in a granule, the probe accessibility may be an issue and report a number than is lower than the actual biological scenario.

3. Fig 2C- The clustering analysis revealed four different patterns of mRNA distributions - however, the way the clusters have been defined is extremely confusing. The definitions of small broad, and consistently small, and so on and so forth needs to be stated clearly. What are the thresholds being used to distinguish small from large, and broad? Also how are these thresholds determined?

4. The random colocalization percentages are very high (Suppl Fig 5B). What accounts for such large variability - the expression levels of the different transcripts?

5. What is a bit confusing is that even after subtracting the random colocalization, the colocalization still dependent completely on the abundance levels of the transcripts rather than a more controlled colocalization. This prompts the question whether the background subtraction was sufficient to account for the chance interactions based on numbers only.

6. Besides the puncta area distributions, did the authors plot the puncta intensity distributions. Can they be fitted to a single or multiple Gaussian distribution? And how does that distribution correlate with the puncta area distribution?

7. The details about the colocalization analysis and the calculations of the intensity and the distributions of the puncta should be elaborated further in Materials and Methods.

8. For the association of mRNAs with the FMRP granules, it would be ideal to show how these PSD95 RNA granules change in their size and colocalization with other mRNAs in a FMRP KO background. This would provide some indication about the biology, whether RBPs are truly driving the organization of the granules or are these co-localization frequencies just stochastic interactions.

Reviewer 2

In this manuscript, the authors investigate the spatial distribution of 15 neuropil-localized mRNAs in the rodent hippocampus through the use of highly multiplexed single-molecule fluorescence in situ hybridization (HiPlex smFISH) and colocalization analyses. They focus on mRNAs that are targets of FMRP and show that colocalization of these mRNAs is largely dependent on their abundance in the neuropil. Simulations are also performed and analyzed to complement the experimental data shown.

The techniques used provide an efficient method of studying multiple RNAs at once, and the findings are generally well conveyed. It seems that the main conclusion is that abundance determines colocalization. The manuscript has several gaps, such as missing controls and unclear explanations, that, once addressed, will significantly improve its clarity and scientific validity.

Major comments:

• Introduction: "These data from intact rodent hippocampal neuropil are generally in agreement with imaging studies from cultured neurons..." The use of the word "generally" is vague, and which studies are being referred to here is unclear.

• Materials and methods: Explanation as to why Sprague Dawley rats were used for Arc dilution studies, while C57BL/6J mice were used for HiPlex, Shank2, and 3plex studies is lacking. Also missing information as to whether male and female mice were used in equal quantities for experiments involving mice, or if one sex was used over the other at times.

• Definition of colocalization and explain why different from other published colocalization methods that use the distance between the light centroids of two signals in nm, for example, PMID: 28223507.

• Figure 1E: Quantifications of # Arc puncta claim an increase in the number of puncta more distally for 1 and 1:2 dilutions; however, images from Figure 1D do not support this.

• Supplementary figure 1B: Much more (~471) Shank2-Pan mRNAs are detected than Shank2e and 2a mRNAs. One would expect the numbers to be more similar given than each mRNA isoform is labelled at both ends. It would be good to show if the Shank2-Pan probe is binding any other mRNAs and giving false signals, or test different probe sequences or regions to ensure the specificity of the Shank2-Pan probes.

• Supplementary figure 1: Quantification of the number of Shank2 mRNA detected is provided in the text, but it would be good to include in the figure.

• HiPlex smFISH: Lacks a control image and quantification to show that there is no signal remaining after cleaving fluorophores from the previous round and prior to fluorescent labelling of the next four targets. The authors chose to probe first for the most abundant mRNAs (for example, pffla3 after camk2a), and therefore, this control is an example. In this case, the negative control provided is not applicable since you do not expect to see DapB RNA in any conditions. Thus, one can't discern the difference between signal being cleaved and washed between rounds or signal having never been there in the first place from the DapB images provided.

• Figure 2AB: The text mentions that Figure 2AB are plots of the relative percent size distributions; however, on the figure, they are actually representative images.

• Figure 4E: Missing explanation or proof that RNA puncta are added in a truly random fashion.

• Fig 3D and Supplementary Figure 8: The nature of the data is unclear (e.g., mean, median, SEM, confidence intervals).

• Correcting multiple comparisons: Corrections such as Holm-Bonferroni should be performed when multiple t-tests are performed on one data set to control the family-wise error rate.

Minor comments:

• HiPlex smFISH: % ethanol samples were dehydrated in is missing

• Arc mRNAs contain multiple copies of Arc transcripts header is confusing

• There is no sup fig 1

## Author Response

Synthesis of Reviews:

Both Reviewers have positive views of the manuscript, and both also raise concerns that need to be addressed in order for the study's conclusions to be sufficiently supported. Please ensure that all of the individual comments below are fully addressed in any revised submission.

We sincerely thank the reviewing editor and both reviewers for the positive and constructive feedback on our manuscript. The insightful comments were helpful as we revised the manuscript to more clearly and impactfully present our work. Below, we addressed each of the reviewers' concerns thoroughly, including additional analyses, supplemental figures, and text edits. In addition to providing excerpts from the revised manuscript, we include line numbers referencing the edits (highlighted in yellow) in the revised word document. Thank you for the opportunity to be considered for publication in eNeuro.

Reviewer 1 This study addresses an important unanswered question in the field of RNA localization in neurons about the nature of the RNA granules. The authors utilize an imaging approach of 12 synaptically localized mRNAs and utilize 2D co-localization as a metric for determining whether mRNAs associates with each other in same or different granules and identified new insights into the heterogeneity of dendritic RNA puncta in the hippocampus. They focused on transcripts that are FMRP targets and found that mRNAs are present in heterogeneous copy number states and exhibit spatial clustering that is dependent on their abundance in the dendrites. While this is a descriptive study using 15 synaptically localized mRNAs, it performs extensive characterization on the distribution of different FMRP targets in the neuropil across three different hippocampal regions. Their conclusions are that the colocalization is guided by abundance only. However, further analysis and experiments are needed to prove that indeed stochastic interactions and not the RNA or RBP biology is the main driver for RNA granule organization. Here are some of the major comments:

We regret that our conclusion (neuropil RNA colocalization is best explained by abundance) came across as the only factor guiding colocalization. We softened descriptions where this strong of a conclusion may have inadvertently been drawn and addressed these alternatives in the discussion. [Line 919-925] Discussion/ mRNA spatial distributions influence stochastic interactions "It is likely that RBPs influence RNP organization, through directly regulating mRNA abundance or through other mechanisms...We also cannot rule out whether other mRNA-specific features (such as sequence length, 5' and 3' UTR properties, RNA folding, translation efficiency etc.), may also be contributing to the observed high levels of colocalization for highly abundant mRNAs." We totally agree with the reviewer's point that further experiments are required to prove whether other factors, such as RBPs, influence RNA granule organization. Indeed, we have begun experiments comparing FMRP KO and WT mice under different behavioral conditions, which are intended for a follow up manuscript and thus out of the scope of the current study. To address this critique, we provide additional discussion of the anticipated outcomes in the revised manuscript (see full response to similar critique Reviewer 1 #8 below).

1. The study assumes that overlapping signals represent true heterotypic granules, which may not be the case. The authors quantify overlapping puncta which are >1% overlap between two channels. Should it be at least >50% overlap, maybe even 75%. How did the authors choose 1%? Although they mention that the effect of RNA abundance remained the same with 50% overlap, one would expect that they would carry out the analysis with at least 50% overlap to begin with. Does the classification of the granules change once this overlapping criterion is used? Also, what is the 2D distance the 1% overlap refers to? We chose a lenient definition of colocalization in order to facilitate an efficient implementation of pairwise and multiple RNA colocalization analyses across 12 mRNA channels. The scale of our dataset imposed a challenge for identifying software capable of streamlining visualization, segmentation, and colocalization analysis for 14 channels (including DAPI and FMRP). NIS Elements allowed us to process this large dataset efficiently, but did not have customizable commands to perform centroid based colocalization analysis between channels. We regret that in our initial submission the threshold for colocalized puncta between channels was mistakenly described as 1% pixel overlap instead of 1 pixel overlap. This is the default and non-editable setting for assessing object-based colocalization in the NIS elements image analysis software (v5.41.01). We revised the methods and results sections to clearly describe the rationale and methods for colocalization. See excerpts below for the better description of the colocalization metric and our enhanced discussion of its limitations. [Line 193-198] Methods/ RNAscope Shank2 smFISH, image acquisition, and analysis "Colocalization analysis: To quantify colocalized mRNA puncta in between two individual channels, the "having" command in NIS elements AR software (v5.41.01) was used to create a new intersection binary layer that includes any object in channel 1 that overlaps any object in channel 2 by at least 1 pixel. This binary layer was then used to count the number of overlapping puncta between two mRNA channels. Thus, colocalization was defined as any segmented mRNA puncta in channel 1 having at least 1 pixel overlap with any segmented mRNA puncta in channel 2." [Line 860-865] Discussion/ mRNA colocalization within the neuropil scales with mRNA abundance "...our data relies on a lenient colocalization metric (1 pixel overlap) based on 2D overlapping in situ signals limited by 250 nm x-y resolution. This method presumably overestimates true levels of colocalization for heterotypic granules as noted by the high colocalization values from both experimental and random quantifications. Indeed, when we analyzed the 3Plex data using lenient (>1% overlap) versus more stringent colocalization criteria (>50% overlap), we observed a reduced number of colocalization events. However, the colocalized granules are still stratified by abundance, validating our primary results. Nevertheless, additional super-resolution techniques (i.e., STORM) are required to prove whether any mRNAs investigated in this study are physically clustering within the same granule (<250 nm)." With regards to 1% versus 50% overlap, this analysis was performed in response to a previous reviewer's comment with similar concerns. Due to the complexity of analyzing the entire HiPlex dataset, we used the 3plex dataset and compared 1% vs 50% colocalization calculated in Python using centroid to centroid 2D distance between two puncta from two channels. Although applying a more stringent cut off resulted in a decreased number of observations, the correlation we uncovered with RNA abundance from the HiPlex analysis (1 pixel overlap) did not change when using 1% or 50% overlap as assessed by similar hierarchical clustering of the colocalization values. Thus, the stratification of granules by abundance did not change with a more stringent % colocalization as Adcy1/Ppp1r9b colocalized significantly more than Adcy1/Rgs14 and Ppp1r9b/Rgs14 at both >1% and >50% overlap definitions. [Line 709-720] Results/ Variation in neuropil mRNA abundance is sufficient to scale mRNA colocalization across cell types "Acknowledging the limitations of our definition of colocalization, we next tested whether using a 2D centroid-based definition of colocalization (Batish et al., 2012; Eliscovich et al., 2017) would reproduce the effect of abundance on colocalization. For every puncta pair, a 2D centroid-based distance less than 50% of the sum of the radii (>50% overlap) was considered colocalized. A similar calculation was done to compare puncta colocalization with >1% overlap. Defining colocalization at >50% or >1% overlap did not change the relationship of mRNA colocalization with abundance (Figure 5-1). Two highly abundant mRNAs, Adcy1 and Ppp1r9b, colocalized significantly more than Rgs14/Adcy1 and Rgs14/Ppp1r9b pairs using both definitions of colocalization (Figure 5-1A). Moreover, when expressed as a % of mRNA colocalized, hierarchical clustering revealed that mRNA pairs clustered together regardless of % overlap indicating a similar influence of abundance on pairwise colocalization (Figure 5-1B). It should be noted that the more stringent definition of overlap decreased the number of colocalized mRNA puncta indicating that 1% overlap likely overestimates the true mRNA-mRNA colocalization within heterotypic RNPs." Figure 5-1 (Refers to Figure 5): 2D centroid-to-centroid distance based analysis reveals mRNA pairwise colocalization stratifies by abundance.

A. 3Plex mRNA pairwise colocalization is expressed as a percentage of the combined total mRNA puncta count in DG. The two more abundant mRNAs, Adcy1 and Ppp1r9b, are colocalized (7.62 {plus minus} 1.45%) significantly more than Adcy1/Rgs14 (0.89 {plus minus} 0.22%) and Rgs14/Ppp1r9b (0.57 {plus minus} 0.07%) when colocalization is defined as >1% overlap (overall effect of mRNA pair, RM ANOVA: F = 55.86, p = 0.0001, N=4 mice) or >50% overlap (overall effect of mRNA pair, RM ANOVA, F = 28.80, p = 0.0008, N=4 mice). Stats were run on the transformed (log10) values as plotted to meet the normality assumption. Tukey's post hoc tests are shown on the plot. **p<0.01; ***p<0.001 B. Heatmap showing the percent mRNA puncta colocalized at >1% and >50% overlap for each mRNA (averaged across N=4 mice +/- SEM, "_50" represents >50% overlap). Data hierarchically cluster by RNA and stratify by abundance (purple shading).

2. It seems that maximum extent of colocalization they can achieve with the smFISH RNAscope probes is approx. 30- 35%, and it is a technical limitation of the system. The fact that it happened for Shank2 and Arc mRNAs suggests that technical difficulties in accessing the transcripts effectively is a hurdle to achieving actual co-localization frequencies. For example, if two mRNAs are tightly packed in a granule, the probe accessibility may be an issue and report a number that is lower than the actual biological scenario.

We think several factors contribute to this technical caveat (see below). Nevertheless, we take the reviewers point that accessibility could, in theory, be an issue for tightly packed heterotypic granules as well. We now explicitly cover this possibility in the discussion (see below). While the absolute numbers and/or percentages of colocalization may be underestimated, the relative differences between RNAs may still be informative, if granules are similarly inaccessible. We include discussion of this alternative as well. [Lines 890-905] Discussion/ mRNA colocalization within the neuropil scales with mRNA abundance "...we only observed ~40% colocalization when RNAscope probes were targeted against a single transcript. Thus, it is plausible that colocalization across transcripts may also be constrained by probe inaccessibility for tightly packed heterotypic granules. There are several alternative explanations that suggest otherwise. First, the samples are heavily digested with a protease. There is evidence that the proteolytic step before hybridization can increase mRNA detection efficiency by permeabilizing tissues after fixation and releasing RNAs from RBPs (Buxbaum et al., 2014; Young et al., 2020). Secondly, the RNAscope puncta counts are very similar to quantities reported from RNA-seq datasets and FISH studies detecting the same transcripts but with different probe designs (Farris et al., 2019; Glock et al., 2021). This indicates that we can reliably detect the vast majority of neuropil localized mRNAs for each species, even when multiplexed, making it unlikely that we are missing (or not detecting) significant populations of mRNAs due to inaccessibility. Lastly, we were able to detect 2 mRNAs (Psd, Camk2a) and 1 granule marker protein (FMRP) spatially overlapping at levels higher than random. Even within this subset of FMRP-defined RNPs, we observed a similar relationship of mRNA colocalization with abundance that we identified earlier from visualizing the entire neuropil mRNA population. Therefore, if all heterotypic granules are similarly affected by inaccessibility, we assume that the relative differences in pairwise colocalization between these granules is an informed estimate of the underlying biological scenario." 3. Figure 2C - The clustering analysis revealed four different patterns of mRNA distributions - however, the way the clusters have been defined is extremely confusing. The definitions of "small broad", and consistently small, and so on and so forth need to be stated clearly. What are the thresholds being used to distinguish small from large, and broad? Also how are these thresholds determined? We regret that our description of the methods for clustering the mRNA area distributions were not clear. The clusters were defined based on the dendrogram that represents the Euclidean distances of the relative % area distributions. The cluster descriptions (peak and average total relative percent across area bins 0.6-1.0 µm2 and >1.0 µm2) were based on the RNA averages within each cluster. We revised the methods, results, and added more information in Table 2 to make these points clearer (see below). [Lines 282-288] Methods/ RNAscope HiPlex smFISH image analysis/ HiPlex Puncta area analysis "...To hierarchically cluster the data based on similarities in their puncta area distribution patterns, we imported the average relative % area distribution data for all 12 mRNAs written as rows in a csv file to R (v4.2.2) and used "Euclidean" distance (function: "dist" in R base package) and "ward.D2" clustering (function "hclust" in R base package) technique. Heatmaps were generated in R using the ComplexHeatmap package (v2.15.2). We describe each of the four clusters by their peak relative % area bin (0.1-1.0 µm2) and the average total relative % across area bins 0.6-1.0 µm2 and >1.0 µm2. The data are presented as the average relative % +/- SEM of the RNAs per cluster." Table 2 (Refers to Figure 3): Average median fluorescent puncta area and normalized total puncta intensity of the HiPlex mRNAs by size cluster. Puncta area data are from N=4 mice and intensity data from N=2 mice. *Clusters were determined via hierarchical clustering of the relative % area distributions.

4. The random colocalization percentages are very high (previous Suppl Fig. 5B, current Figure 3-1B). What accounts for such large variability - the expression levels of the different transcripts? The high percentage of random colocalization, in particular for the most abundant transcripts as noted by the reviewer, is likely a consequence of our lenient definition of colocalization. This is now explicitly discussed as a technical limitation (see excerpt below). We also present data whereby subsetting for FMRP-defined puncta considerably decreased the random colocalization percentages, but not the experimental colocalization percentages, resulting in a similar correlation between abundance and colocalization as described for all puncta. We provide more context to address these points in the discussion as detailed below. [Lines 876 - 889] Discussion/ mRNA colocalization within the neuropil scales with mRNA abundance "...the image rotation method also results in some very high levels of random colocalization (>50%), in particular for the highly expressed mRNAs, calling into question the robustness of the metric and/or the resulting random subtracted percentages. To account for this in the multi-transcript colocalization analyses, where ~65% of any mRNA randomly colocalized with at least one other mRNA, we subsetted the data to puncta defined by the presence of FMRP protein. The percent of FMRP-defined Psd puncta colocalized with any mRNA at random was considerably lower (25.3%) compared to all Psd puncta (65.1%). However, in experimental images, the percent of FMRP-defined Psd puncta colocalized with any mRNA (86.4%) was similar to the percent without FMRP (91.9%), indicating the robustness of assessing multi-RNA containing puncta compared to pairs. Within this subset of FMRP-defined Psd mRNA puncta, we also found that mRNA colocalization stratified by abundance. For example, FMRP- defined Psd puncta containing highly abundant Camk2a colocalized more (~20% vs random 5%) than those containing lower abundant Ppfia3 (0.6% vs random 0.3%). These data indicate that the high level of random colocalization was reduced when mRNA puncta were categorized as FMRP-defined RNPs, further validating our primary result." 5. What is a bit confusing is that even after subtracting the random colocalization, the colocalization still dependent completely on the abundance levels of the transcripts rather than a more controlled colocalization. This prompts the question whether the background subtraction was sufficient to account for the chance interactions based on numbers only.

We acknowledge this limitation in the results and discussion. To better control for the effect of abundance on colocalization, we also present that data as a percentage of the sum of both RNAs. The relationship between abundance and RNA colocalization is also evident in this analysis. [Lines 866 - 875] Discussion/ mRNA colocalization within the neuropil scales with mRNA abundance "...the image rotation method for random subtraction does not fully recapitulate the cytoarchitecture (e.g., extracellular spaces, organelles, etc.) present in properly registered images that theoretically restricts the potential locations for overlap, which will result in an underestimation of random colocalization, thereby inflating the random-subtracted colocalization percentage observed. This is evident in the pairwise comparisons near random that are always above 0. To address this, we also plotted the data as a percentage of total mRNA puncta in the pair, as done previously (Batish et al., 2012) to better account for the effect of mRNA abundance. This analysis showed that 24.5 % of Camk2a and Ddn (two highest abundant in our dataset) are colocalized in CA2 neuropil whereas, only 3.5% of Pum2 and Ppfia3 (two least abundant in our dataset) are colocalized. Thus, the relationship between abundance and RNA colocalization is also evident in analyses that control for abundance substantiating our initial results." 6. Besides the puncta area distributions, did the authors plot the puncta intensity distributions? Can they be fitted to a single or multiple Gaussian distribution? And how does that distribution correlate with the puncta area distribution? Based on the reviewer's comment, we plotted the puncta intensity distributions for the 12 mRNAs from N=2 mice (one mouse per HiPlex run) and included the analyses as Extended Data Figure 3-4. The total intensity values per puncta were normalized to the mean puncta intensity per channel. The distributions were non- normal. We correlated the raw and normalized puncta intensity versus puncta area for one representative mRNA per cluster (Figure 3-4AB). Hierarchical clustering of the relative % intensity distributions produced the same 4 clusters of mRNAs as the area distributions (Figure 3-4C). (see excerpt below) Figure 3-4 (Refers to Figure 3): mRNA puncta intensity distributions mirror mRNA puncta area distributions A. Correlation plots of individual mRNA puncta area and raw total intensity from representative candidate mRNAs from each cluster from a representative mouse (small: Calm1, small broad: Psd, large broad: Adcy1, large: Dlg4).

B. Same as (A) but with puncta total intensity normalized by mean intensity.

C. Heatmap of relative % distribution of normalized puncta total intensity for 12 mRNA channels (Ave +/- SEM, N= 2 mice). Hierarchical clustering of the intensity % distributions revealed the same 4 clusters as the puncta area distributions.

We also added clear descriptions of how the intensity distribution data were quantified and plotted (see methods below). [Lines 290-303] Methods/ RNAscope HiPlex smFISH image analysis "HiPlex Puncta intensity analysis: To assess fluorescent puncta intensity distributions, we pasted the segmented puncta binary layer from the processed images onto the raw images and exported the mean intensity (average pixel intensity in each punctum) and total fluorescent intensity (total of all pixel intensities in that punctum) of each binary object from each channel for N=2 mice (1 mouse per HiPlex run). Although similar probe designs (20 zz pairs, ~1100 basepair target region length) were used for each mRNA, the range of total intensities per puncta varied based on image acquisition parameters that varied per mRNA per experimental run. To compare fluorescent puncta intensities across mRNAs, we divided the puncta total intensity value by the mean intensity of the puncta in each mRNA channel that resulted in a normalized range of total intensity per puncta. For each individual punctum, the normalized total intensity theoretically should give an arbitrary value that correlates with the area of the puncta on a given image (9.6679 pixels per µm). Therefore, we then correlated raw and normalized total intensity values with puncta areas. Next, we plotted the relative % intensity distributions with the same bin width for all 12 mRNAs. The average relative % distribution of normalized total intensity per puncta of each mRNA was then plotted on a heatmap. The same hierarchical clustering (distance: Euclidean, clustering: ward.D2) was used as above to identify mRNAs with similar puncta intensity distributions." 7. The details about the colocalization analysis and the calculations of the intensity and the distributions of the puncta should be elaborated further in Materials and Methods.

We thoroughly revised the text to clearly describe the area and intensity distribution data and colocalization analyses. For methods excerpts, see previous responses to Reviewer 1 #1 for colocalization analyses, #3 for puncta area, and #6 for puncta intensity analyses.

8. For the association of mRNAs with the FMRP granules, it would be ideal to show how these PSD95 RNA granules change in their size and colocalization with other mRNAs in a FMRP KO background. This would provide some indication about the biology, whether RBPs are truly driving the organization of the granules or are these co-localization frequencies just stochastic interactions.

We completely agree that data from FMRP KO mice would be compelling to assess the role of FMRP in regulating target RNA puncta size and colocalization patterns. While out of the scope of the current study, we discussed potential anticipated outcomes based on our data and the current literature on the effects of FMRP KO on target mRNA abundance and colocalization (see excerpt below). [Lines 944 - 952] Discussion/ Relevance to FMRP "While it is beyond the scope of the current study, future perturbation experiments are required to assess whether mRNA colocalization is affected by the loss of FMRP. There is radioactive in situ hybridization evidence for a trending reduction in Dlg4 mRNA in CA1 distal dendrites of adult Fmr1 knockout (KO) mice (Zalfa et al., 2007). Several recent transcriptomic studies have also reported small, consistent decreases in FMRP target mRNA abundance in Fmr1 KO mice hippocampus (Hale et al., 2021; Sawicka et al., 2019; Thomson et al., 2017). Combining these findings with our data, we expect that a lack of FMRP will decrease hippocampal neuropil mRNA abundance for a subset of these 12 mRNA targets, and therefore, decrease mRNA colocalization patterns selectively for those affected mRNAs in relation to others. This would suggest that FMRP may regulate heterotypic RNP compositions through mRNA abundance, although other FMRP-mediated mechanisms may also contribute (Korb et al., 2017)." Reviewer 2 In this manuscript, the authors investigate the spatial distribution of 15 neuropil-localized mRNAs in the rodent hippocampus through the use of highly multiplexed single-molecule fluorescence in situ hybridization (HiPlex smFISH) and colocalization analyses. They focus on mRNAs that are targets of FMRP and show that colocalization of these mRNAs is largely dependent on their abundance in the neuropil. Simulations are also performed and analyzed to complement the experimental data shown.

The techniques used provide an efficient method of studying multiple RNAs at once, and the findings are generally well conveyed. It seems that the main conclusion is that abundance determines colocalization. The manuscript has several gaps, such as missing controls and unclear explanations, that once addressed, will significantly improve its clarity and scientific validity.

Major comments: imaging studies from cultured neurons..." The use of the word "generally" is vague, and which studies are being referred to here is unclear.

We revised the statement to provide additional details on the data in agreement with our findings and cited relevant literature. [Lines 99-102] Introduction "These data from intact rodent hippocampal neuropil are generally in agreement with imaging studies from cultured neurons that show mRNAs in neuronal dendrites are present in distinctly sized mRNPs (Donlin-Asp et al., 2021; Tübing et al., 2010) and dendritic mRNA spatial proximity clustering can be partially explained by their distribution (Wang et al., 2020), suggesting that both systems are subject to similar intrinsic mechanisms that favor independent localization with stochastic overlaps as opposed to coordinated assembly of these RNAs into selective multimeric granules in the neuropil." 1. Materials and methods: Explanation as to why Sprague Dawley rats were used for Arc dilution studies, while C57BL/6J mice were used for HiPlex, Shank2, and 3plex studies is lacking. Also missing information as to whether male and female mice were used in equal quantities for experiments involving mice, or if one sex was used over the other at times.

We revised the methods section to include information on species and sex. The rat study was completed several years ago in a different laboratory (Steward lab) by the senior author and served as motivation for investigating mRNA puncta size. [Lines 108-110] Methods/ Animals "An adult female Sprague Dawley rat was used for the Arc dilution study. P17 male mice were used for HiPlex RNAscope experiments. Adult (8-16 weeks) male mice were used for Shank2 and 3plex/ multiplex RNAscope experiments." 2. Definition of colocalization and explain why it is different from other published colocalization methods that use the distance between the light centroids of two signals in nm, for example, PMID: 28223507.

We revised the methods and discussion sections to better describe how colocalization was defined and the rationale for using such a method. Please see our response to Reviewer 1 # 1 for the definition of colocalization. We also added rationale for choosing this particular method over other published methods (see excerpt below). [Lines 224 - 230] Methods/ RNAscope Shank2 smFISH/ Random colocalization "...due to the scale of our dataset (66 pairwise quantification for 12 mRNA channels) and our goal to quantify multi-transcript containing RNPs in addition to pairwise colocalization, we chose the semi- automated pixel overlap approach within NIS elements (defined above) as opposed to the commonly used centroid-to-centroid distance-based calculation of colocalization (Batish et al., 2012; Eliscovich et al., 2017). Although, for a subset of our data, we performed the 2D centroid distance based colocalization as described below for the 3plex dataset analysis." [Lines 373-378] Methods/ RNAscope 3Plex smFISH image analysis/ Centroid-based colocalization analysis "Centroid to centroid 2D distance between two puncta from two channels was calculated using "cdist" function from scipy.spatial package. Colocalization was defined as >1% overlap (if the distance between centroid XY coordinates of two puncta from two channels is less than 0.99 X sum of radii (feret's diameter/2) of each puncta pair) and >50% overlap (if the distance between centroid XY each puncta pair) as a syntax using NumPy in python." 3. Figure 1E: Quantifications of # Arc puncta claims an increase in the number of puncta more distally for 1 and 1:2 dilutions; however, images from Figure 1D do not support this.

Thanks to the reviewer for pointing out this inconsistency. We re-inspected the images and determined that the juxtaposition of the inner molecular layer images to the cell body layer negatively impacted threshold based segmentation. The signal in the cell body layer in 1X and 0.5X dilutions is oversaturated in order to visualize Arc mRNA puncta in the neuropil. There are a few cells in each inner molecular image that skew the signal to noise such that fewer puncta were segmented. We repeated the dilution count analyses with smaller regions of interest placed farther from the cell body layer. The resulting updated plot in Figure 1E is normalized to the average number of puncta across 1X ECS images and shows the trending decrease in count more distally as visually represented in Figure 1D. Plots in Figure 1B and 1F were also updated with data generated from the revised thresholds based on the smaller ROIs. Notably, none of the conclusions drawn from this data were impacted by the revised analyses. [Figure 1 Legend Line 1262] Figure 1: Arc mRNA fluorescent puncta diameter, number, and intensity upon probe dilution reveal multiple pools of mRNAs.

A. Representative images of Arc mRNA localization to the middle molecular layer (MM) of the rat dentate gyrus following ECS and 60 min unilateral HFS. The fluorescence signal in the cell body layer is saturated to visualize the fluorescence signal in the dendrites. Scale = 25 µm.

B. Quantification of the Feret's diameter of localized Arc mRNA puncta (HFS condition) vs. non-localized puncta (ECS) by distance from the cell body layer (CB, white dashed lines). Average diameter (normalized to ECS) {plus minus} SEM per layer from n=3 technical replicates. number and intensity or apparent size of fluorescent puncta.

D. Representative images after ECS only labeled with 1X undiluted full length Arc probe or serially diluted with unlabeled full length Arc probe and imaged with identical exposure times, 300 ms (Left). Images acquired with doubling exposure times (600, 1200, 2400 ms) revealed undetected puncta at 300 ms (Right), indicating a decrease in puncta intensity as would be expected with multiple RNAs per puncta. Inverted inset scale = 2.5 µm.

E. Quantification of Arc puncta number for each dilution at 300 ms (normalized to 1X). Stepwise decrease in Arc puncta number suggests low copy number containing puncta. Average number {plus minus} SEM per layer from n=3 technical replicates.

F. Quantification of Arc puncta Feret's diameter, intensity, number for the middle molecular layer of each dilution at 300 ms. Normalized average {plus minus} SEM from n=3 technical replicates. Representative MM layer inverted images inset in D.

4. Supplementary figure 1B: Much more (~471) Shank2-Pan mRNAs are detected than Shank2e and 2a mRNAs. One would expect the numbers to be more similar given that each mRNA isoform is labelled at both ends. It would be good to show if the Shank2-Pan probe is binding any other mRNAs and giving false signals, or test different probe sequences or regions to ensure the specificity of the Shank2-Pan probes.

Per the RNAscope product website (see except below), it is highly unlikely for a non-target molecule to have a ~44 nt sequence that can bind two ZZ probe pairs for signal amplification. If the greater number of Shank2-Pan signals is not due to increased accessibility of the 3'UTR compared to the 5'UTR, it is most likely due to additional Shank2 mRNA isoforms not different mRNA species or false signals. (https://acdbio.com/science/how-it-works) "RNAscope employs a probe design strategy much akin to fluorescence resonance energy transfer (FRET), in which two independent probes (double Z probes) have to hybridize to the target sequence in tandem in order for signal amplification to occur. As it is highly unlikely that two independent probes will hybridize to a non- specific target right next to each other, this design concept ensures selective amplification of target-specific signals. For each target RNA species, ~20 double Z target probe pairs are designed to specifically hybridize to the target molecule, but not to non-targeted molecules." 5. Supplementary figure 1: Quantification of the number of Shank2 mRNA detected is provided in the text, but it would be good to include in the figure.

As suggested, we now include the number of Shank2 mRNA in Figure 2D. [Figure 2 Legend Line 1262]. Based on editorial policies for extended data figures, we moved this figure into the main text.

Figure 2. Shank2 isoform-specific 5' probes are highly colocalized with the Pan 3' probe.

A. Shank2 isoform gene models with RNAseq read depth data showing the relative expression levels in hippocampal CA2. Sequences from either long (Shank2e) or short (Shank2a) transcripts targeted by different 5' probes (magenta and green, respectively) and both targeted by the Pan 3' probe (yellow) are shown.

B. Representative image of the three Shank2 probes in CA2 cell bodies. Nuclei are labeled with DAPI (blue).

White arrows indicate example transcriptional foci. Dashed white box is the inset showing a transcriptional focus labeled by all three probes.

C. High-magnification images of (i) Shank2e, (ii) Shank2a, (iii) Shank2-Pan and (iv) the merged image.

Arrows indicate example colocalization of Shank2-Pan 3' probe with either Shank2e 5' probe (white arrows) or Shank2a 5' probe (cyan arrows) as shown below.

D. Shank2e, Shank2a and Shank2-pan mRNA puncta count in the CA2 cell body layer.

E. Quantification of the % colocalization between the Shank2e (magenta) and Shank2a (green) or both (yellow) with the Shank2-Pan probe (open circles) compared to that observed by random colocalization (closed circles). % of Shank2-Pan colocalized with either Shank2a or Shank2e (orange) compared to random colocalization and the % of Shank2e colocalizing with Shank2a (red) compared to random, many of which are transcription foci, as shown in B.

Error bars indicate SEM; N=3 mice; * denotes p <0.05 from paired one-tailed t-test. Scale bars: B) 5 µm, 1 µm, C) 5 µm, 1 µm.

6. HiPlex smFISH: Lacks a control image and quantification to show that there is no signal remaining after cleaving fluorophores from the previous round and prior to fluorescent labelling of the next four targets. The authors chose to probe first for the most abundant mRNAs (for example, ppfia3 after camk2a), and therefore, this control is an example. In this case, the negative control provided is not applicable since you do not expect to see DapB RNA in any conditions. Thus, one can't discern the difference between signal being cleaved and washed between rounds or signal having never been there in the first place from the DapB images provided.

Because of the time involved for iteratively imaging HiPlex RNAscope (~1 week for running 2 replicates with appropriate controls) we did not image in between every round after cleavage of fluorophores as that would add 3 more rounds of imaging on top of the current 4 rounds. However, we imaged slides after cleavage of round 3 fluorophores to use for background subtraction and provide representative images for the reviewer below. Fluorophore cleavage successfully removed all signals irrespective of mRNA signal abundance.

7. Figure 2AB: The text mentions that Figure 2AB are plots of the relative percent size distributions; however, on the figure, they are actually representative images.

We revised the text to refer to Figure 3C (previously referred to as Fig 2C). [Lines 523-525] "Based on the findings from our previous experiments (Figure 1), which indicated the presence of distinctly sized populations of homotypic RNA puncta within the same mRNA species, we first calculated the median fluorescent puncta area (Table 2) and plotted the relative % area distributions (Figure 3C)." 8. Figure 4E: Missing explanation or proof that RNA puncta are added in a truly random fashion.

We revised the methods to include details regarding the function(s) used to generate random Adcy1 puncta. We provide the resulting bootstrapped images from one iteration as a figure for the reviewers to show the puncta are randomly distributed. [Lines 387-396] Methods/ RNAscope 3Plex smFISH image analysis/ Simulated colocalization analysis "A list of synthetic data points (the number of which equaled the difference of Adcy1 mRNA puncta between CA2 and DG images in the same section) was generated from bootstrap-sampled values from the minimum-to- maximum range for CA2 image puncta area, feret's diameter, and total intensity per puncta using "sample(n=..., replace=True).values" function. Centroid (X,Y) coordinates for these synthetic data points were uniformly drawn at random (without replacement) within the 0-180 µm X and Y ROIs using "np.random.uniform" function (no duplicate centroid coordinates were used, either in the randomly generated values or the experimental dataset). Then, this simulated data was appended to the CA2 experimental dataset to generate the CA2 simulated dataset. We plotted CA2 experimental and simulated data points using the "sns.scatterplot" function to visually inspect that the XY coordinates were added randomly." 9. Fig 3D and Supplementary Figure 8: The nature of the data is unclear (e.g., mean, median, SEM, confidence intervals).

The experimental and random pie charts are presented as mean {plus minus} SEM. This information was added to the figure legends (Figure 4, Figure 4-4). [Legend, Line 1262] 10. Correcting multiple comparisons: Corrections such as Holm-Bonferroni should be performed when multiple t-tests are performed on one data set to control the family-wise error rate.

We regret the oversight. We used the two-stage step-up method of Benjamini, Krieger, Yekutieli with 1% false discovery rate (FDR) to correct for multiple comparisons. Figure 4-5 legend and the statistical analyses section have been updated accordingly. [Lines 411-414] Methods/ Statistical analyses "........When ANOVA statistical tests are reported, Tukey's or Dunn's multiple comparison post-hoc tests are shown on the figure plots and described in the figure legend accordingly. When multiple t-tests are reported, the two-stage step-up method of Benjamini, Krieger, Yekutieli with 1% false discovery rate (FDR) was used for correcting for multiple comparisons." Minor comments: • HiPlex smFISH: % ethanol samples were dehydrated in is missing 50%, 70%, and 100% ethanol (twice) were used for sequential dehydration steps. This information was added to the methods. [Lines 234-235] • Arc mRNAs contain multiple copies of Arc transcripts header is confusing The header was edited to more accurately state "Arc mRNA puncta contain..." [Line 417] • There is no sup fig 1 We confirmed that Figure 2 (previously referred to as Supplemental Figure 1 i.e Shank2 figure) is included [Legend, Line 1264].

References:

Batish, M., van den Bogaard, P., Kramer, F. R., &Tyagi, S. (2012). Neuronal mRNAs travel singly into dendrites. Proceedings of the National Academy of Sciences, 109(12), 4645-4650. https://doi.org/10.1073/pnas.1111226109 Eliscovich, C., Shenoy, S. M., &Singer, R. H. (2017). Imaging mRNA and protein interactions within neurons.

Proceedings of the National Academy of Sciences of the United States of America, 114(10), E1875- E1884. https://doi.org/10.1073/pnas.1621440114 Hale, C. R., Sawicka, K., Mora, K., Fak, J. J., Kang, J. J., Cutrim, P., Cialowicz, K., Carroll, T. S., &Darnell, R.

B. (2021, December 23). FMRP regulates mRNAs encoding distinct functions in the cell body and dendrites of CA1 pyramidal neurons. eLife; eLife Sciences Publications Limited. https://doi.org/10.7554/eLife.71892 Sawicka, K., Hale, C. R., Park, C. Y., Fak, J. J., Gresack, J. E., Van Driesche, S. J., Kang, J. J., Darnell, J. C., &Darnell, R. B. (2019). FMRP has a cell-type-specific role in CA1 pyramidal neurons to regulate autism-related transcripts and circadian memory. eLife, 8, e46919. https://doi.org/10.7554/eLife.46919 Thomson, S. R., Seo, S. S., Barnes, S. A., Louros, S. R., Muscas, M., Dando, O., Kirby, C., Wyllie, D. J. A., Hardingham, G. E., Kind, P. C., &Osterweil, E. K. (2017). Cell-Type-Specific Translation Profiling Reveals a Novel Strategy for Treating Fragile X Syndrome. Neuron, 95(3), 550-563.e5. https://doi.org/10.1016/j.neuron.2017.07.013

## References

[B1] Ahn H, Durang X, Shim JY, Park G, Jeon J, Park HY (2023) Statistical modeling of mRNP transport in dendrites: a comparative analysis of β-actin and Arc mRNP dynamics. Traffic 24:522–532. 10.1111/tra.12913 37545033 PMC10946522

[B2] Ainsley JA, Drane L, Jacobs J, Kittelberger KA, Reijmers LG (2014) Functionally diverse dendritic mRNAs rapidly associate with ribosomes following a novel experience. Nat Commun 5:4510. 10.1038/ncomms5510 25072471 PMC4160876

[B3] Antar LN, Dictenberg JB, Plociniak M, Afroz R, Bassell GJ (2005) Localization of FMRP-associated mRNA granules and requirement of microtubules for activity-dependent trafficking in hippocampal neurons. Genes Brain Behav 4:350–359. 10.1111/j.1601-183X.2005.00128.x 16098134

[B4] Batish M, van den Bogaard P, Kramer FR, Tyagi S (2012) Neuronal mRNAs travel singly into dendrites. Proc Natl Acad Sci U S A 109:4645–4650. 10.1073/pnas.1111226109 22392993 PMC3311338

[B5] Bauer KE, et al. (2019) Live cell imaging reveals 3′-UTR dependent mRNA sorting to synapses. Nat Commun 10:3178. 10.1038/s41467-019-11123-x 31320644 PMC6639396

[B6] Bauer KE, Bargenda N, Schieweck R, Illig C, Segura I, Harner M, Kiebler MA (2022) RNA supply drives physiological granule assembly in neurons. Nat Commun 13:2781. 10.1038/s41467-022-30067-3 35589693 PMC9120520

[B7] Bauer KE, de Queiroz BR, Kiebler MA, Besse F (2023) RNA granules in neuronal plasticity and disease. Trends Neurosci 46:525–538. 10.1016/j.tins.2023.04.004 37202301

[B8] Bergmann C, Mousaei K, Rizzoli SO, Tchumatchenko T (2025) How energy determines spatial localisation and copy number of molecules in neurons. Nat Commun 16:1424. 10.1038/s41467-025-56640-0 39915472 PMC11802781

[B9] Boeynaems S, et al. (2019) Spontaneous driving forces give rise to protein-RNA condensates with coexisting phases and complex material properties. Proc Natl Acad Sci U S A 116:7889–7898. 10.1073/pnas.1821038116 30926670 PMC6475405

[B10] Bolte S, Cordelières FP (2006) A guided tour into subcellular colocalization analysis in light microscopy. J Microsc 224:213–232. 10.1111/j.1365-2818.2006.01706.x 17210054

[B11] Bradshaw KD, Emptage NJ, Bliss TVP (2003) A role for dendritic protein synthesis in hippocampal late LTP. Eur J Neurosci 18:3150–3152. 10.1111/j.1460-9568.2003.03054.x 14656312

[B12] Buxbaum AR, Wu B, Singer RH (2014) Single β-actin mRNA detection in neurons reveals a mechanism for regulating its translatability. Science 343:419–422. 10.1126/science.1242939 24458642 PMC4121734

[B13] Cajigas IJ, Tushev G, Will TJ, tom Dieck S, Fuerst N, Schuman EM (2012) The local transcriptome in the synaptic neuropil revealed by deep sequencing and high-resolution imaging. Neuron 74:453–466. 10.1016/j.neuron.2012.02.036 22578497 PMC3627340

[B14] Carson JH, Gao Y, Tatavarty V, Levin MK, Korza G, Francone VP, Kosturko LD, Maggipinto MJ, Barbarese E (2008) Multiplexed RNA trafficking in oligodendrocytes and neurons. Biochim Biophys Acta 1779:453–458. 10.1016/j.bbagrm.2008.04.002 18442491 PMC2584806

[B15] Cirillo D, Agostini F, Klus P, Marchese D, Rodriguez S, Bolognesi B, Tartaglia GG (2013) Neurodegenerative diseases: quantitative predictions of protein–RNA interactions. RNA 19:129–140. 10.1261/rna.034777.112 23264567 PMC3543085

[B16] Cougot N, Bhattacharyya SN, Tapia-Arancibia L, Bordonné R, Filipowicz W, Bertrand E, Rage F (2008) Dendrites of mammalian neurons contain specialized P-body-like structures that respond to neuronal activation. J Neurosci 28:13793–13804. 10.1523/JNEUROSCI.4155-08.2008 19091970 PMC6671906

[B17] Darnell JC, et al. (2011) FMRP stalls ribosomal translocation on mRNAs linked to synaptic function and autism. Cell 146:247–261. 10.1016/j.cell.2011.06.013 21784246 PMC3232425

[B18] Dictenberg JB, Swanger SA, Antar LN, Singer RH, Bassell GJ (2008) A direct role for FMRP in activity-dependent dendritic mRNA transport links filopodial-spine morphogenesis to fragile X syndrome. Dev Cell 14:926–939. 10.1016/j.devcel.2008.04.003 18539120 PMC2453222

[B19] Donlin-Asp PG, Polisseni C, Klimek R, Heckel A, Schuman EM (2021) Differential regulation of local mRNA dynamics and translation following long-term potentiation and depression. Proc Natl Acad Sci U S A 118:e2017578118. 10.1073/pnas.2017578118 33771924 PMC8020670

[B20] Dunn KW, Kamocka MM, McDonald JH (2011) A practical guide to evaluating colocalization in biological microscopy. Am J Physiol Cell Physiol 300:C723–C742. 10.1152/ajpcell.00462.2010 21209361 PMC3074624

[B21] Eliscovich C, Shenoy SM, Singer RH (2017) Imaging mRNA and protein interactions within neurons. Proc Natl Acad Sci U S A 114:E1875–E1884. 10.1073/pnas.1621440114 28223507 PMC5347572

[B22] Elvira G, et al. (2006) Characterization of an RNA granule from developing brain *. Mol Cell Proteomics 5:635–651. 10.1074/mcp.M500255-MCP200 16352523

[B23] Evans PR, et al. (2018) Interactome analysis reveals regulator of G protein signaling 14 (RGS14) is a novel calcium/calmodulin (Ca2+/CaM) and CaM kinase II (CaMKII) binding partner. J Proteome Res 17:1700–1711. 10.1021/acs.jproteome.8b00027 29518331 PMC6554723

[B24] Farris S, Lewandowski G, Cox CD, Steward O (2014) Selective localization of Arc mRNA in dendrites involves activity- and translation-dependent mRNA degradation. J Neurosci 34:4481–4493. 10.1523/JNEUROSCI.4944-13.2014 24671994 PMC3965778

[B25] Farris S, Ward JM, Carstens KE, Samadi M, Wang Y, Dudek SM (2019) Hippocampal subregions express distinct dendritic transcriptomes that reveal differences in mitochondrial function in CA2. Cell Rep 29:522–539.e6. 10.1016/j.celrep.2019.08.093 31597108 PMC6894405

[B26] Fatimy RE, Davidovic L, Tremblay S, Jaglin X, Dury A, Robert C, Koninck PD, Khandjian EW (2016) Tracking the fragile X mental retardation protein in a highly ordered neuronal ribonucleoparticles population: a link between stalled polyribosomes and RNA granules. PLoS Genet 12:e1006192. 10.1371/journal.pgen.1006192 27462983 PMC4963131

[B27] Fernandez-Moya SM, Bauer KE, Kiebler MA (2014) Meet the players: local translation at the synapse. Front Mol Neurosci 7:84. 10.3389/fnmol.2014.00084 25426019 PMC4227489

[B28] Fernandopulle MS, Lippincott-Schwartz J, Ward ME (2021) RNA transport and local translation in neurodevelopmental and neurodegenerative disease. Nat Neurosci 24:622–632. 10.1038/s41593-020-00785-2 33510479 PMC8860725

[B29] Ford L, Ling E, Kandel ER, Fioriti L (2019) CPEB3 inhibits translation of mRNA targets by localizing them to P bodies. Proc Natl Acad Sci U S A 116:18078–18087. 10.1073/pnas.1815275116 31416913 PMC6731686

[B30] Fritzsche R, et al. (2013) Interactome of two diverse RNA granules links mRNA localization to translational repression in neurons. Cell Rep 5:1749–1762. 10.1016/j.celrep.2013.11.023 24360960

[B31] Frye HE, et al. (2021) Sex differences in the role of CNIH3 on spatial memory and synaptic plasticity. Biol Psychiatry 90:766–780. 10.1016/j.biopsych.2021.07.014 34548146 PMC8571071

[B32] Gao Y, Tatavarty V, Korza G, Levin MK, Carson JH (2008) Multiplexed dendritic targeting of α calcium calmodulin-dependent protein kinase II, neurogranin, and activity-regulated cytoskeleton-associated protein RNAs by the A2 pathway. Mol Biol Cell 19:2311–2327. 10.1091/mbc.e07-09-0914 18305102 PMC2366844

[B33] Garcia-Jove Navarro M, Kashida S, Chouaib R, Souquere S, Pierron G, Weil D, Gueroui Z (2019) RNA is a critical element for the sizing and the composition of phase-separated RNA–protein condensates. Nat Commun 10:3230. 10.1038/s41467-019-11241-6 31324804 PMC6642089

[B34] Glock C, Biever A, Tushev G, Bartnik I, Nassim-Assir B, tom Dieck S, Schuman EM (2020) The mRNA translation landscape in the synaptic neuropil (p. 2020.06.09.141960). bioRxiv. 10.1101/2020.06.09.141960 PMC8639352 34670838

[B35] Glock C, Biever A, Tushev G, Nassim-Assir B, Kao A, Bartnik I, Tom Dieck S, Schuman EM (2021) The translatome of neuronal cell bodies, dendrites, and axons. Proc Natl Acad Sci U S A 118:e2113929118. 10.1073/pnas.2113929118 34670838 PMC8639352

[B36] Hafner A-S, Donlin-Asp PG, Leitch B, Herzog E, Schuman EM (2019) Local protein synthesis is a ubiquitous feature of neuronal pre- and postsynaptic compartments. Science 364:eaau3644. 10.1126/science.aau3644 31097639

[B37] Hale CR, Sawicka K, Mora K, Fak JJ, Kang JJ, Cutrim P, Cialowicz K, Carroll TS, Darnell RB (2021) FMRP regulates mRNAs encoding distinct functions in the cell body and dendrites of CA1 pyramidal neurons. Elife 10:e71892. 10.7554/eLife.71892 34939924 PMC8820740

[B38] Heraud-Farlow JE, et al. (2013) Staufen2 regulates neuronal target RNAs. Cell Rep 5:1511–1518. 10.1016/j.celrep.2013.11.039 24360961

[B39] Hildyard JCW, Rawson F, Wells DJ, Piercy RJ (2020) Multiplex in situ hybridization within a single transcript: RNAscope reveals dystrophin mRNA dynamics. PLoS One 15:e0239467. 10.1371/journal.pone.0239467 32970731 PMC7514052

[B40] Huang Y-S, Carson JH, Barbarese E, Richter JD (2003) Facilitation of dendritic mRNA transport by CPEB. Genes Dev 17:638–653. 10.1101/gad.1053003 12629046 PMC196011

[B41] Huber KM, Kayser MS, Bear MF (2000) Role for rapid dendritic protein synthesis in hippocampal mGluR-dependent long-term depression. Science 288:1254–1257. 10.1126/science.288.5469.1254 10818003

[B42] Huber KM, Gallagher SM, Warren ST, Bear MF (2002) Altered synaptic plasticity in a mouse model of fragile X mental retardation. Proc Natl Acad Sci U S A 99:7746–7750. 10.1073/pnas.122205699 12032354 PMC124340

[B43] Jiang Y-H, Ehlers MD (2013) Modeling autism by SHANK gene mutations in mice. Neuron 78:8–27. 10.1016/j.neuron.2013.03.016 23583105 PMC3659167

[B44] Kanai Y, Dohmae N, Hirokawa N (2004) Kinesin transports RNA: isolation and characterization of an RNA-transporting granule. Neuron 43:513–525. 10.1016/j.neuron.2004.07.022 15312650

[B45] Kiebler MA, Bassell GJ (2006) Neuronal RNA granules: movers and makers. Neuron 51:685–690. 10.1016/j.neuron.2006.08.021 16982415

[B46] Kiebler MA, Bauer KE (2024) RNA granules in flux: dynamics to balance physiology and pathology. Nat Rev Neurosci 25:711–725. 10.1038/s41583-024-00859-1 39367081

[B47] Knowles RB, Sabry JH, Martone ME, Deerinck TJ, Ellisman MH, Bassell GJ, Kosik KS (1996) Translocation of RNA granules in living neurons. J Neurosci 16:7812–7820. 10.1523/JNEUROSCI.16-24-07812.1996 8987809 PMC6579227

[B48] Korb E, Herre M, Zucker-Scharff I, Gresack J, Allis CD, Darnell RB (2017) Excess translation of epigenetic regulators contributes to fragile X syndrome and is alleviated by Brd4 inhibition. Cell 170:1209–1223.e20. 10.1016/j.cell.2017.07.033 28823556 PMC5740873

[B49] Krichevsky AM, Kosik KS (2001) Neuronal RNA granules: a link between RNA localization and stimulation-dependent translation. Neuron 32:683–696. 10.1016/s0896-6273(01)00508-6 11719208

[B50] Lewis YE, Moskovitz A, Mutlak M, Heineke J, Caspi LH, Kehat I (2018) Localization of transcripts, translation, and degradation for spatiotemporal sarcomere maintenance. J Mol Cell Cardiol 116:16–28. 10.1016/j.yjmcc.2018.01.012 29371135

[B51] Maharana S, et al. (2018) RNA buffers the phase separation behavior of prion-like RNA binding proteins. Science 360:918–921. 10.1126/science.aar7366 29650702 PMC6091854

[B52] Martin KC, Ephrussi A (2009) mRNA localization: gene expression in the spatial dimension. Cell 136:719–730. 10.1016/j.cell.2009.01.044 19239891 PMC2819924

[B53] McDonald JH, Dunn KW (2013) Statistical tests for measures of colocalization in biological microscopy. J Microsc 252:295–302. 10.1111/jmi.12093 24117417 PMC4428547

[B54] Mikl M, Vendra G, Kiebler MA (2011) Independent localization of MAP2, CaMKIIα and β-actin RNAs in low copy numbers. EMBO Rep 12:1077–1084. 10.1038/embor.2011.149 21869818 PMC3185336

[B55] Mitsumori K, Takei Y, Hirokawa N (2017) Components of RNA granules affect their localization and dynamics in neuronal dendrites. Mol Biol Cell 28:1412–1417. 10.1091/mbc.e16-07-0497 28404748 PMC5449141

[B56] Miyashiro KY, Beckel-Mitchener A, Purk TP, Becker KG, Barret T, Liu L, Carbonetto S, Weiler IJ, Greenough WT, Eberwine J (2003) RNA cargoes associating with FMRP reveal deficits in cellular functioning in Fmr1 null mice. Neuron 37:417–431. 10.1016/S0896-6273(03)00034-5 12575950

[B57] Monteiro P, Feng G (2017) SHANK proteins: roles at the synapse and in autism spectrum disorder. Nat Rev Neurosci 18:147–157. 10.1038/nrn.2016.183 28179641

[B58] Moon SL, Morisaki T, Khong A, Lyon K, Parker R, Stasevich TJ (2019) Multicolor single-molecule tracking of mRNA interactions with RNP granules. Nat Cell Biol 21:162–168. 10.1038/s41556-018-0263-4 30664789 PMC6375083

[B59] Morris CW, Watkins DS, Shah NR, Pennington T, Hens B, Qi G, Doud EH, Mosley AL, Atwood BK, Baucum AJ (2023) Spinophilin limits metabotropic glutamate receptor 5 scaffolding to the postsynaptic density and cell type specifically mediates excessive grooming. Biol Psychiatry 93:976–988. 10.1016/j.biopsych.2022.12.008 36822932 PMC10191892

[B60] Nakayama K, et al. (n.d.) RNG105/caprin1, an RNA granule protein for dendritic mRNA localization, is essential for long-term memory formation. Elife 6:e29677. 10.7554/eLife.29677 29157358 PMC5697933

[B61] Napoli I, et al. (2008) The fragile X syndrome protein represses activity-dependent translation through CYFIP1, a new 4E-BP. Cell 134:1042–1054. 10.1016/j.cell.2008.07.031 18805096

[B62] Niepielko MG, Eagle WVI, Gavis ER (2018) Stochastic seeding coupled with mRNA self-recruitment generates heterogeneous Drosophila germ granules. Curr Biol 28:1872–1881.e3. 10.1016/j.cub.2018.04.037 29861136 PMC6008217

[B63] Ortiz R, Georgieva MV, Gutiérrez S, Pedraza N, Fernández-Moya SM, Gallego C (2017) Recruitment of Staufen2 enhances dendritic localization of an intron-containing CaMKIIα mRNA. Cell Rep 20:13–20. 10.1016/j.celrep.2017.06.026 28683307

[B64] Park HY, Lim H, Yoon YJ, Follenzi A, Nwokafor C, Lopez-Jones M, Meng X, Singer RH (2014) Visualization of dynamics of single endogenous mRNA labeled in live mouse. Science 343:422–424. 10.1126/science.1239200 24458643 PMC4111226

[B65] Rao VR, Pintchovski SA, Chin J, Peebles CL, Mitra S, Finkbeiner S (2006) AMPA receptors regulate transcription of the plasticity-related immediate-early gene Arc. Nat Neurosci 9:887–895. 10.1038/nn1708 16732277

[B66] Richter JD, Bassell GJ, Klann E (2015) Dysregulation and restoration of translational homeostasis in fragile X syndrome. Nat Rev Neurosci 16:595–605. 10.1038/nrn4001 26350240 PMC4688896

[B67] Ripin N, Parker R (2023) Formation, function, and pathology of RNP granules. Cell 186:4737–4756. 10.1016/j.cell.2023.09.006 37890457 PMC10617657

[B68] Roden C, Gladfelter AS (2021) RNA contributions to the form and function of biomolecular condensates. Nat Rev Mol Cell Biol 22:183–195. 10.1038/s41580-020-0264-6 32632317 PMC7785677

[B69] Sahoo PK, et al. (2018) Axonal G3BP1 stress granule protein limits axonal mRNA translation and nerve regeneration. Nat Commun 9:3358. 10.1038/s41467-018-05647-x 30135423 PMC6105716

[B70] Samadi M, et al. (2023) Mechanisms of mGluR-dependent plasticity in hippocampal area CA2. Hippocampus 33:730–744. 10.1002/hipo.23529 36971428 PMC10213158

[B71] Sauerbeck AD, et al. (2020) SEQUIN multiscale imaging of mammalian central synapses reveals loss of synaptic connectivity resulting from diffuse traumatic brain injury. Neuron 107:257–273.e5. 10.1016/j.neuron.2020.04.012 32392471 PMC7381374

[B72] Sawicka K, Hale CR, Park CY, Fak JJ, Gresack JE, Van Driesche SJ, Kang JJ, Darnell JC, Darnell RB (2019) FMRP has a cell-type-specific role in CA1 pyramidal neurons to regulate autism-related transcripts and circadian memory. Elife 8:e46919. 10.7554/eLife.46919 31860442 PMC6924960

[B73] Sharangdhar T, Sugimoto Y, Heraud-Farlow J, Fernández-Moya SM, Ehses J, Ruiz de los Mozos I, Ule J, Kiebler MA (2017) A retained intron in the 3′-UTR of Calm3 mRNA mediates its Staufen2- and activity-dependent localization to neuronal dendrites. EMBO Rep 18:1762–1774. 10.15252/embr.201744334 28765142 PMC5623867

[B74] Shiina N, Shinkura K, Tokunaga M (2005) A novel RNA-binding protein in neuronal RNA granules: regulatory machinery for local translation. J Neurosci 25:4420–4434. 10.1523/JNEUROSCI.0382-05.2005 15858068 PMC6725113

[B75] Song MS, Moon HC, Jeon J-H, Park HY (2018) Neuronal messenger ribonucleoprotein transport follows an aging Lévy walk. Nat Commun 9:344. 10.1038/s41467-017-02700-z 29367597 PMC5783941

[B76] Steward O, Bakker CE, Willems PJ, Oostra BA (1998) No evidence for disruption of normal patterns of mRNA localization in dendrites or dendritic transport of recently synthesized mRNA in FMR1 knockout mice, a model for human fragile-X mental retardation syndrome. Neuroreport 9:477. 10.1097/00001756-199802160-00022 9512393

[B77] Thomson SR, et al. (2017) Cell-type-specific translation profiling reveals a novel strategy for treating fragile X syndrome. Neuron 95:550–563.e5. 10.1016/j.neuron.2017.07.013 28772121 PMC5548955

[B78] Trcek T, Grosch M, York A, Shroff H, Lionnet T, Lehmann R (2015) Drosophila germ granules are structured and contain homotypic mRNA clusters. Nat Commun 6:7962. 10.1038/ncomms8962 26242323 PMC4918342

[B79] Trcek T, Douglas TE, Grosch M, Yin Y, Eagle WVI, Gavis ER, Shroff H, Rothenberg E, Lehmann R (2020) Sequence-independent self-assembly of germ granule mRNAs into homotypic clusters. Mol Cell 78:941–950.e12. 10.1016/j.molcel.2020.05.008 32464092 PMC7325742

[B80] Tübing F, Vendra G, Mikl M, Macchi P, Thomas S, Kiebler MA (2010) Dendritically localized transcripts are sorted into distinct ribonucleoprotein particles that display fast directional motility along dendrites of hippocampal neurons. J Neurosci 30:4160–4170. 10.1523/JNEUROSCI.3537-09.2010 20237286 PMC6632293

[B81] Van Treeck B, Protter DSW, Matheny T, Khong A, Link CD, Parker R (2018) RNA self-assembly contributes to stress granule formation and defining the stress granule transcriptome. Proc Natl Acad Sci U S A 115:2734–2739. 10.1073/pnas.1800038115 29483269 PMC5856561

[B82] Virtanen P, et al. (2020) Scipy 1.0: fundamental algorithms for scientific computing in Python. Nat Methods 17:261–272. 10.1038/s41592-019-0686-2 32015543 PMC7056644

[B83] Wagle S, Kraynyukova N, Hafner A-S, Tchumatchenko T (2023) Computational insights into mRNA and protein dynamics underlying synaptic plasticity rules. Mol Cell Neurosci 125:103846. 10.1016/j.mcn.2023.103846 36963534 PMC10274545

[B84] Wang F, Flanagan J, Su N, Wang LC, Bui S, Nielson A, Wu X, Vo HT, Ma XJ, Luo Y (2012) RNAscope: a novel in situ RNA analysis platform for formalin-fixed, paraffin-embedded tissues. J Mol Diagn 14:22–29. 10.1016/j.jmoldx.2011.08.002 22166544 PMC3338343

[B85] Wang G, Ang C-E, Fan J, Wang A, Moffitt JR, Zhuang X (2020) Spatial organization of the transcriptome in individual neurons (p. 2020.12.07.414060). bioRxiv. 10.1101/2020.12.07.414060

[B87] Wang H, Zhuo M (2012) Group I metabotropic glutamate receptor-mediated gene transcription and implications for synaptic plasticity and diseases. Front Pharmacol 3:189. 10.3389/fphar.2012.00189 23125836 PMC3485740

[B86] Wang H, Wu L-J, Zhang F, Zhuo M (2008) Roles of calcium-stimulated adenylyl cyclase and calmodulin-dependent protein kinase IV in the regulation of FMRP by group I metabotropic glutamate receptors. J Neurosci 28:4385–4397. 10.1523/JNEUROSCI.0646-08.2008 18434517 PMC6670940

[B88] Wang X, Zeng W, Kim MS, Allen PB, Greengard P, Muallem S (2007) Spinophilin/neurabin reciprocally regulate signaling intensity by G protein-coupled receptors. EMBO J 26:2768–2776. 10.1038/sj.emboj.7601701 17464283 PMC1888664

[B89] Wu L, Wells D, Tay J, Mendis D, Abbott M-A, Barnitt A, Quinlan E, Heynen A, Fallon JR, Richter JD (1998) CPEB-mediated cytoplasmic polyadenylation and the regulation of experience-dependent translation of α-CaMKII mRNA at synapses. Neuron 21:1129–1139. 10.1016/S0896-6273(00)80630-3 9856468

[B90] Young AP, Jackson DJ, Wyeth RC (2020) A technical review and guide to RNA fluorescence in situ hybridization. PeerJ 8:e8806. 10.7717/peerj.8806 32219032 PMC7085896

[B91] Zalfa F, et al. (2007) A new function for the fragile X mental retardation protein in regulation of PSD-95 mRNA stability. Nat Neurosci 10:578–587. 10.1038/nn1893 17417632 PMC2804293

[B92] Zang JB, et al. (2009) A mouse model of the human fragile X syndrome I304N mutation. PLoS Genet 5:e1000758. 10.1371/journal.pgen.1000758 20011099 PMC2779495

[B93] Zeitelhofer M, Karra D, Macchi P, Tolino M, Thomas S, Schwarz M, Kiebler M, Dahm R (2008) Dynamic interaction between P-bodies and transport ribonucleoprotein particles in dendrites of mature hippocampal neurons. J Neurosci 28:7555–7562. 10.1523/JNEUROSCI.0104-08.2008 18650333 PMC6670838

