# Supplementary figures and images for: Multiplexed smFISH Reveals the Spatial Organization of Neuropil Localized mRNAs Is Linked to Abundance
Source: eNeuro. 2025 Dec 9;12(12):ENEURO.0184-25.2025. doi: 10.1523/ENEURO.0184-25.2025 (PMC12700706; doi:10.1523/ENEURO.0184-25.2025)

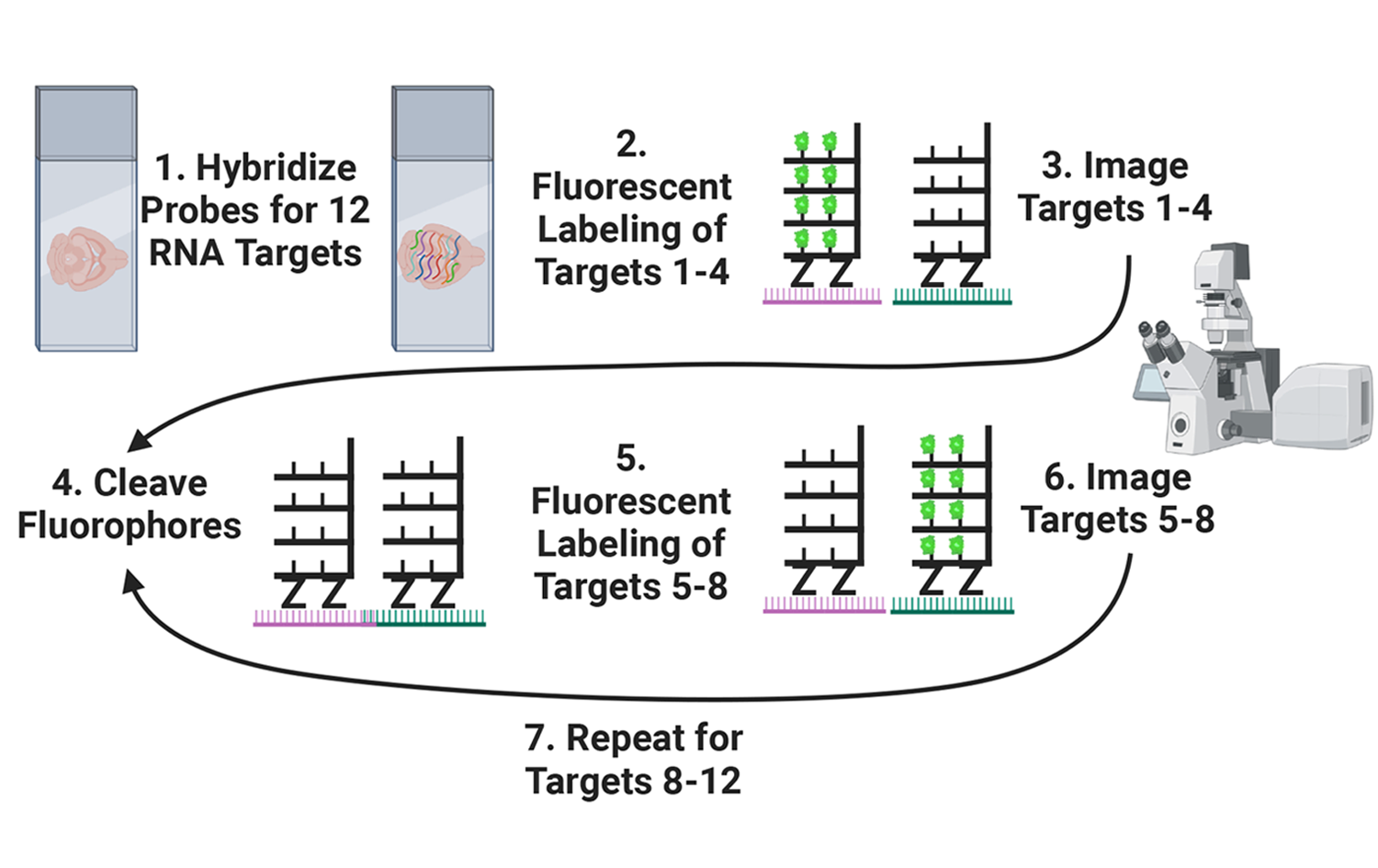

Supplement: Figure 3-1 — (Refers to Figure 3 & 4) Schematic showing workflow of HiPlex smFISH. Download Figure 3-1, TIF file. [file eneuro-12-ENEURO.0184-25.2025-s002.tif]

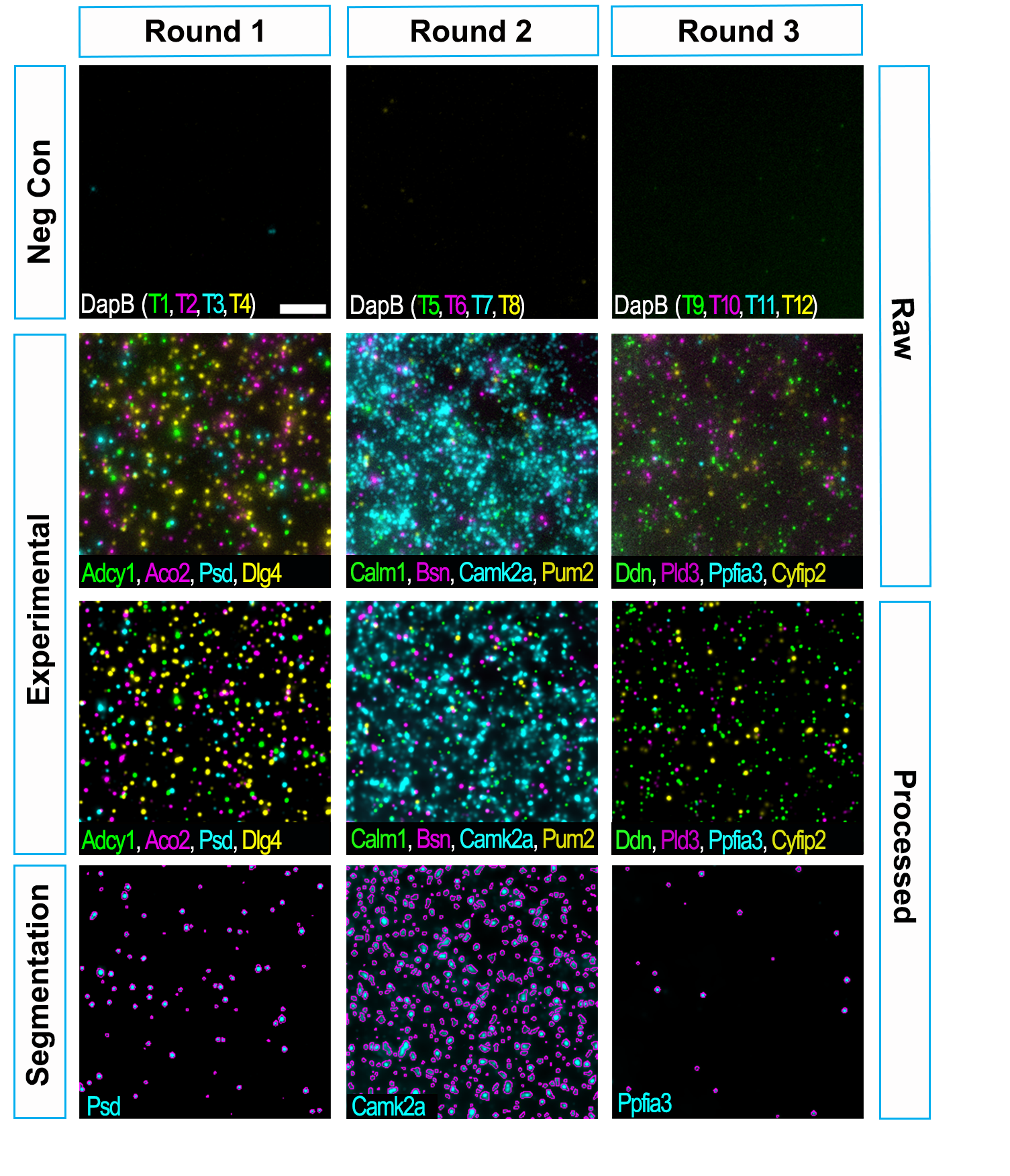

Supplement: Figure 3-2 — (Refers to Figure 3 & 4) HiPlex image processing and segmentation. Raw and processed negative control images probed for the bacterial RNA DapB in each channel. Negative control images were acquired with identical acquisition parameters as experimental images shown below from all three rounds of HiPlex smFISH. Experimental images are presented with the same intensity thresholds as the corresponding negative control channels. The last row displays segmented binary layers for the Psd, Camk2a, and Ppfia3 channels, created using intensity thresholds determined from the negative control image of the corresponding channels in each round. Scale: 5 µm. Download Figure 3-2, TIF file. [file eneuro-12-ENEURO.0184-25.2025-s003.tif]

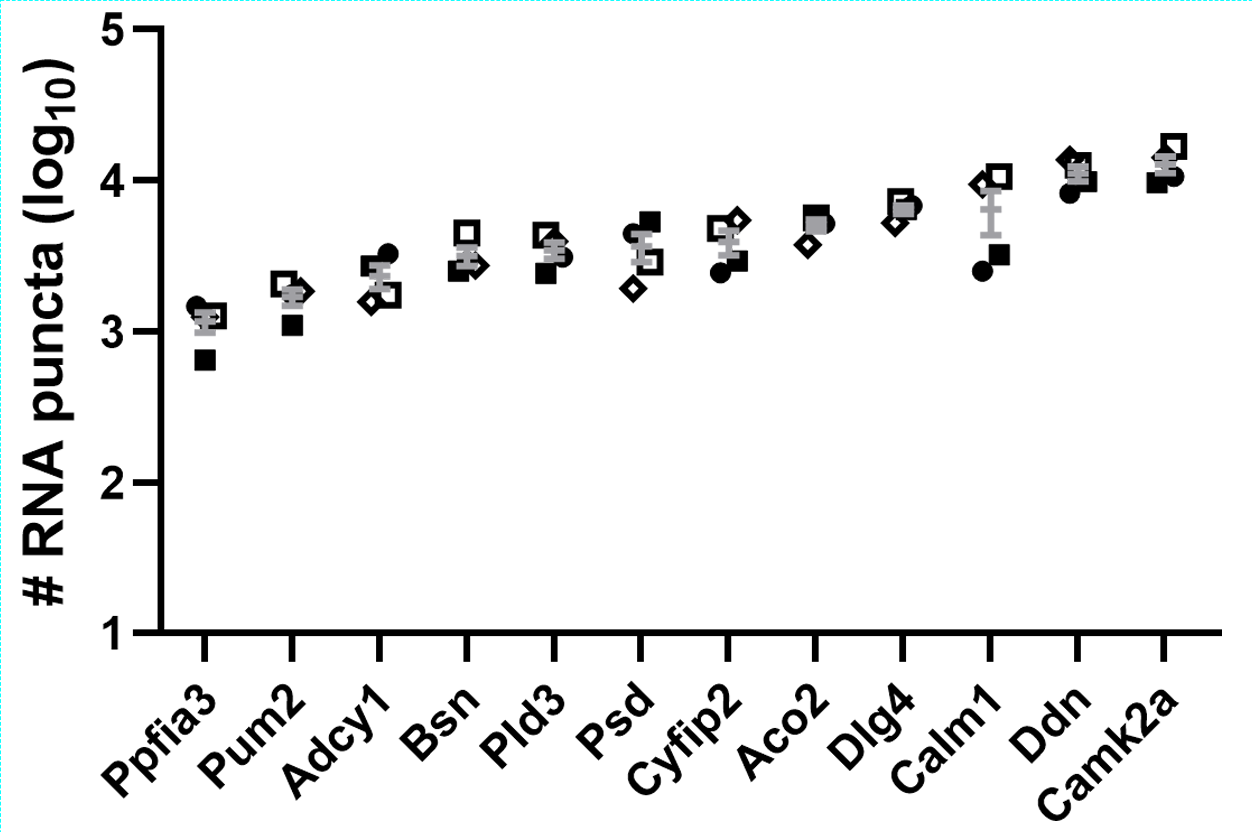

Supplement: Figure 3-3 — (Refers to Figure 3 & 4) Abundance of each mRNA in the CA2 neuropil. Each symbol represents data from a biological replicate (N=4 mice). Error bars indicate SEM. Download Figure 3-3, TIF file. [file eneuro-12-ENEURO.0184-25.2025-s004.tif]

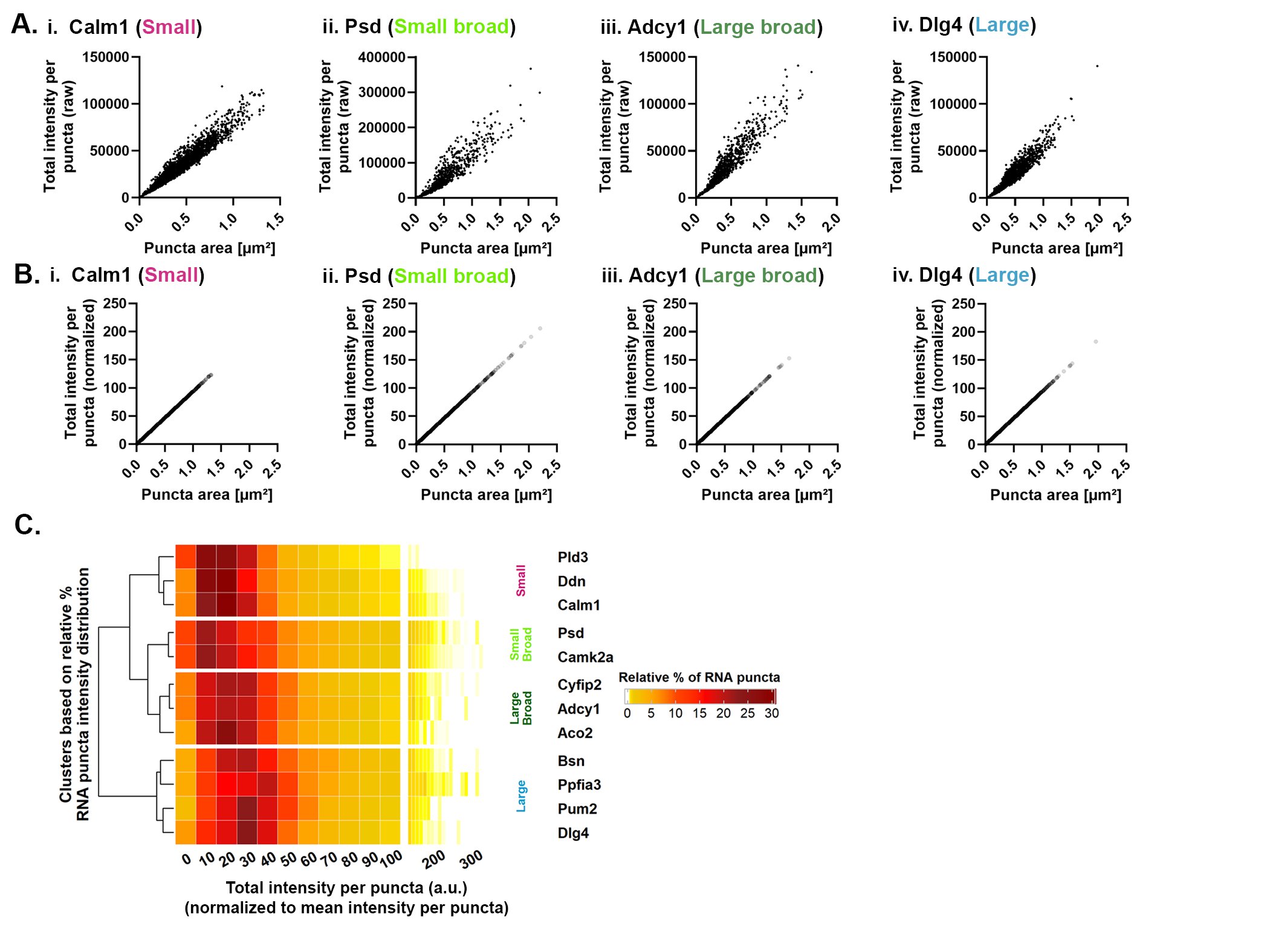

Supplement: Figure 3-4 — (Refers to Figure 3): mRNA puncta intensity distributions mirror mRNA puncta area distributions A. Correlation plots of individual mRNA puncta area and raw total intensity from representative candidate mRNAs from each cluster from a representative mouse (small: Calm1, small broad: Psd, large broad: Adcy1, large: Dlg4). B. Same as (A) but with puncta total intensity normalized by mean intensity. C. Heatmap of relative % distribution of normalized puncta total intensity for 12 mRNA channels (Ave +/- SEM, N = 2 mice). Hierarchical clustering of the intensity % distributions revealed the same 4 clusters as the puncta area distributions. Download Figure 3-4, TIF file. [file eneuro-12-ENEURO.0184-25.2025-s005.tif]

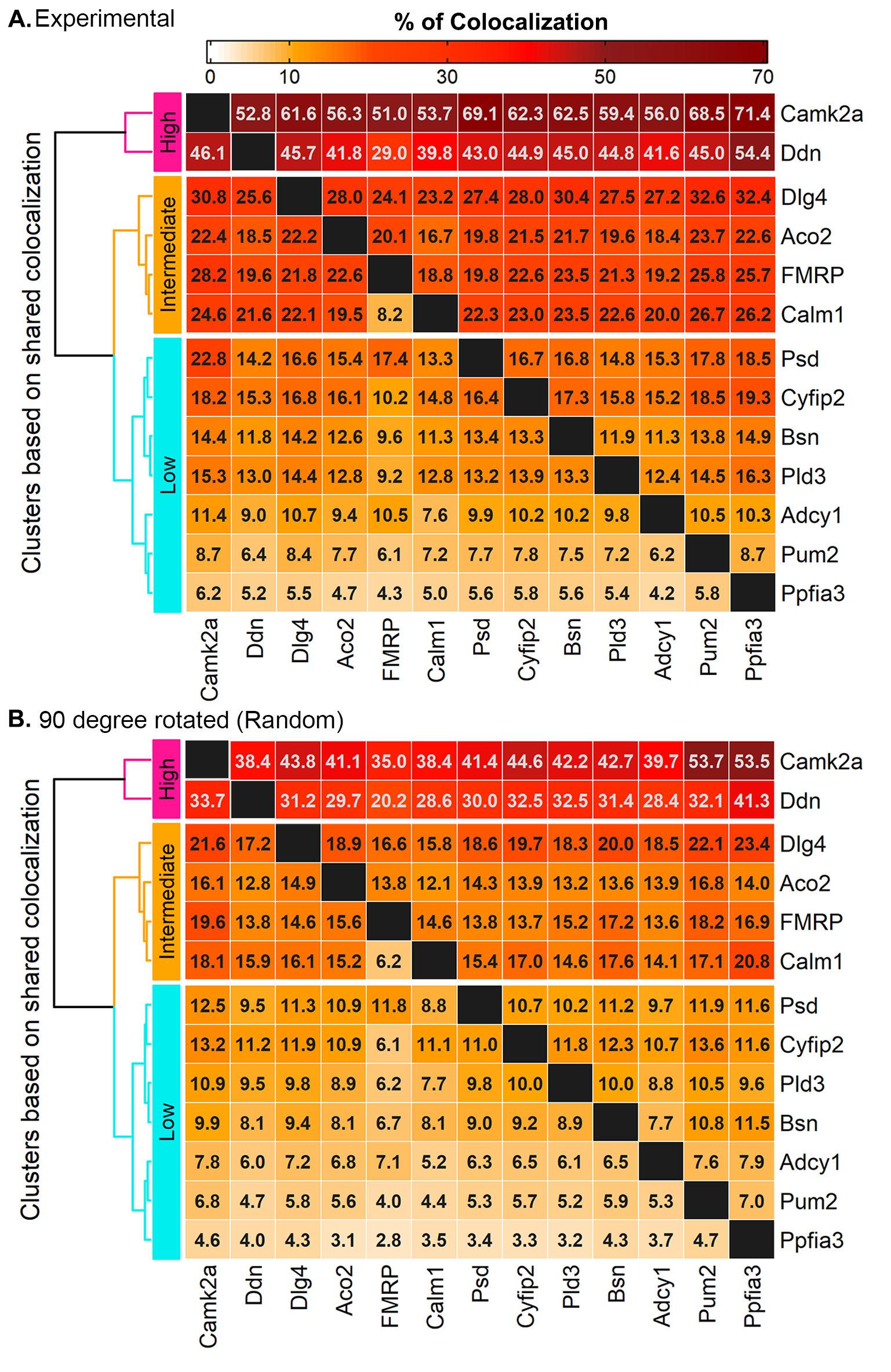

Supplement: Figure 4-1 — (Refers to Figure 4A) Heatmaps showing the total average pairwise colocalization of mRNAs in properly registered (experimental) images (A) and in rotated (random) images (B). The percentage values in each column are calculated by dividing the number of column mRNAs colocalizing with each row mRNA by the total number of column mRNAs, i.e. 5.2% of Ddn colocalizes with Ppfia3, whereas 54.4% of Ppfia3 colocalizes with Ddn before random colocalization subtraction (see Figure 4A). Values are the average of N=4 mice (four 52 X 52 µm2 images averaged per mouse). Download Figure 4-1, TIF file. [file eneuro-12-ENEURO.0184-25.2025-s006.tif]

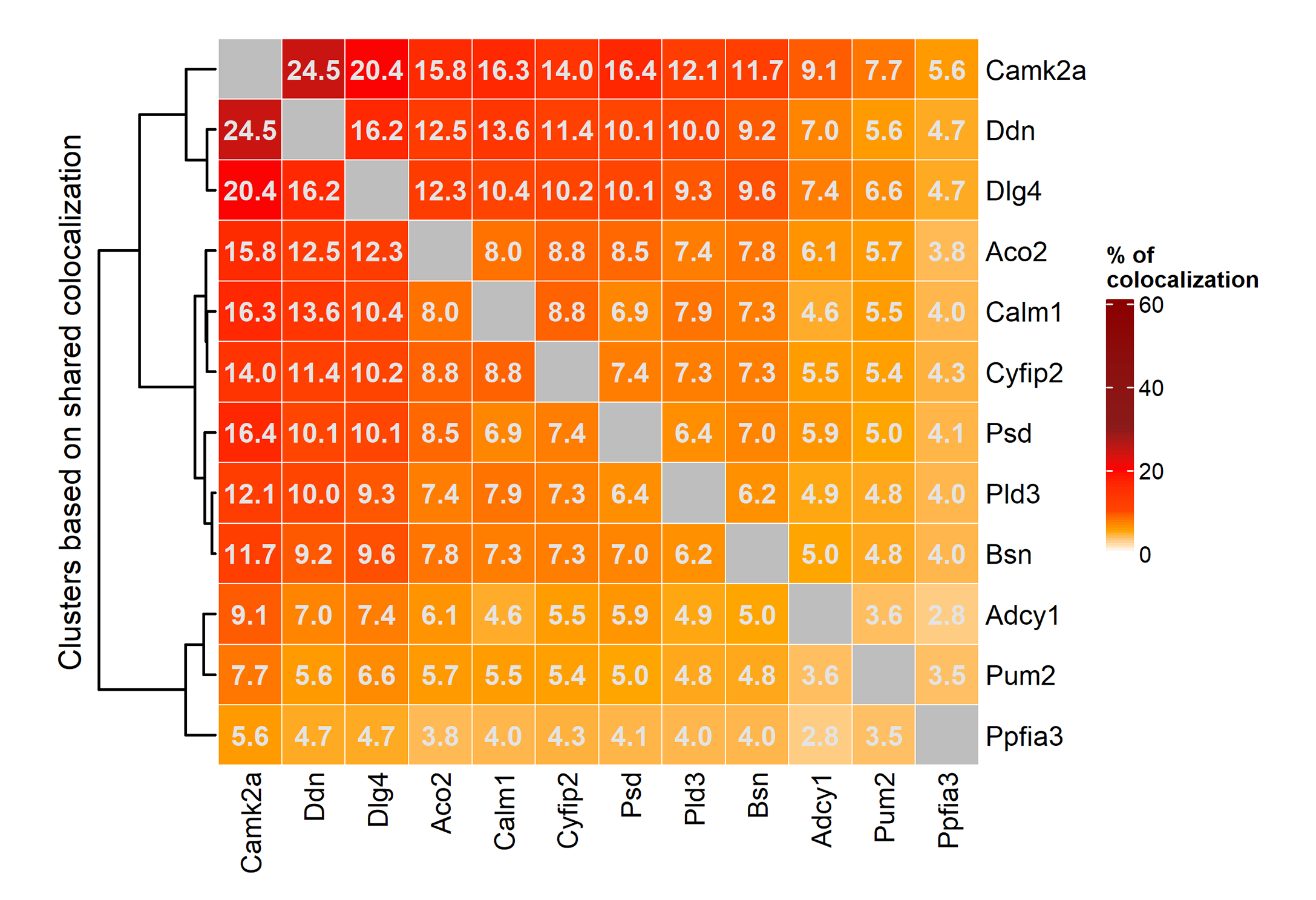

Supplement: Figure 4-2 — (Refers to Figure 4) Pairwise colocalization of neuropil localized mRNAs analyzed as in Batish et al. For each pair of comparisons, the number of overlapping mRNA puncta between two channels was divided by the combined count of the two mRNAs being compared and expressed as a percentage (average of N=4 mice). Hierarchical clustering of the data revealed a very similar pattern (as shown in Figure 4A) showing that every mRNA is colocalized more with highly abundant mRNAs (Camk2a, Ddn, Dlg4) and show fewer instances of colocalization with mRNAs that are of lower abundance (Pum2, Ppfia3). mRNAs in intermediary clusters also show a similar trend although their specific orders are more variable compared to Figure 4A. Download Figure 4-2, TIF file. [file eneuro-12-ENEURO.0184-25.2025-s007.tif]

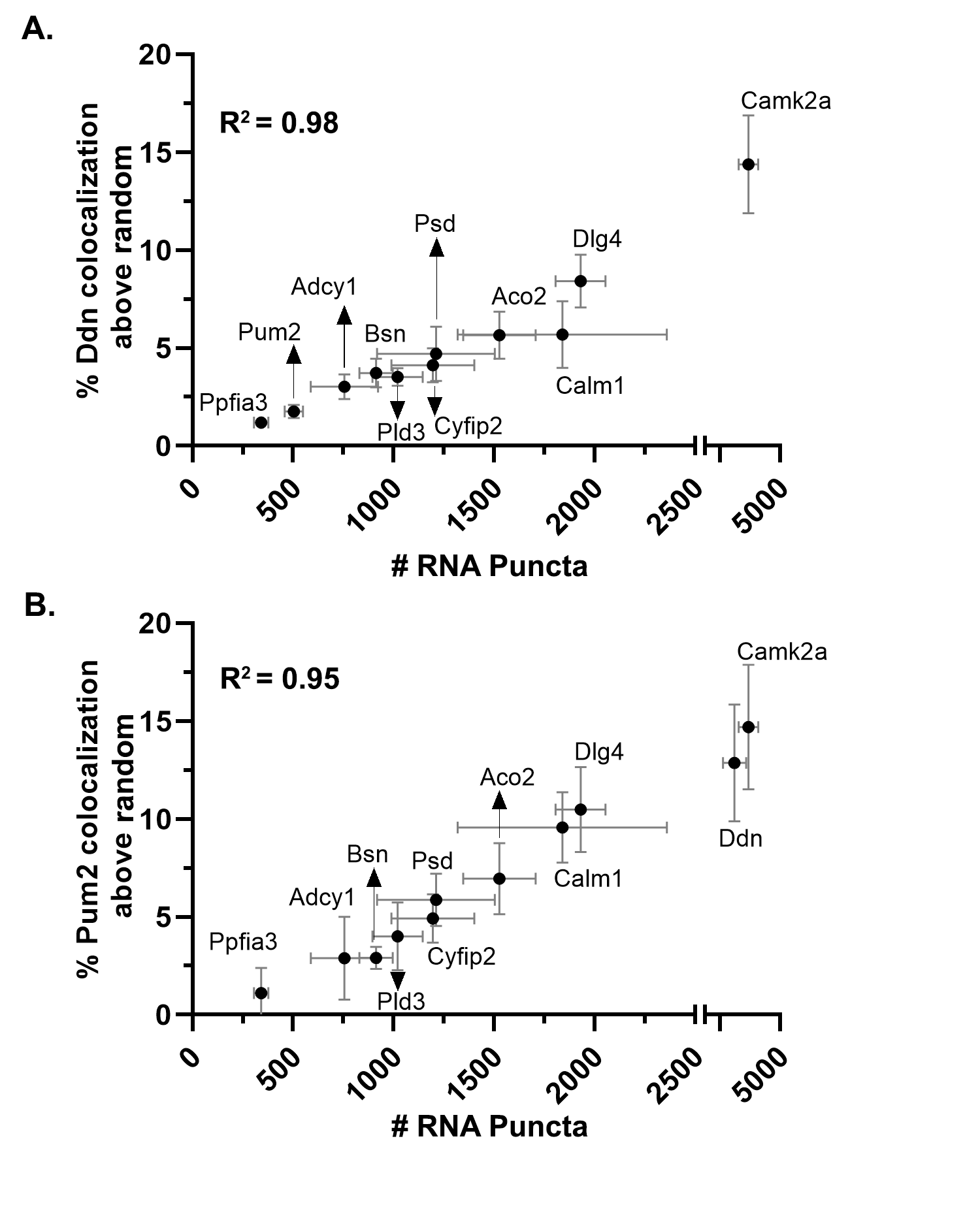

Supplement: Figure 4-3 — (Refers to Figure 4C) The positive correlation between pairwise colocalization and mRNA abundance exists regardless of expression Ddn (A) and Pum2 (B) exhibit high and low abundance, respectively, in CA2 neuropil. However, these mRNAs display a consistent positive correlation between % colocalization (random colocalization subtracted) and the abundance of the 11 paired mRNAs (Ddn R2 = 0.98 and Pum2 R2 = 0.95). (N=4 mice. Error bars indicate SEM.) Download Figure 4-3, TIF file. [file eneuro-12-ENEURO.0184-25.2025-s008.tif]

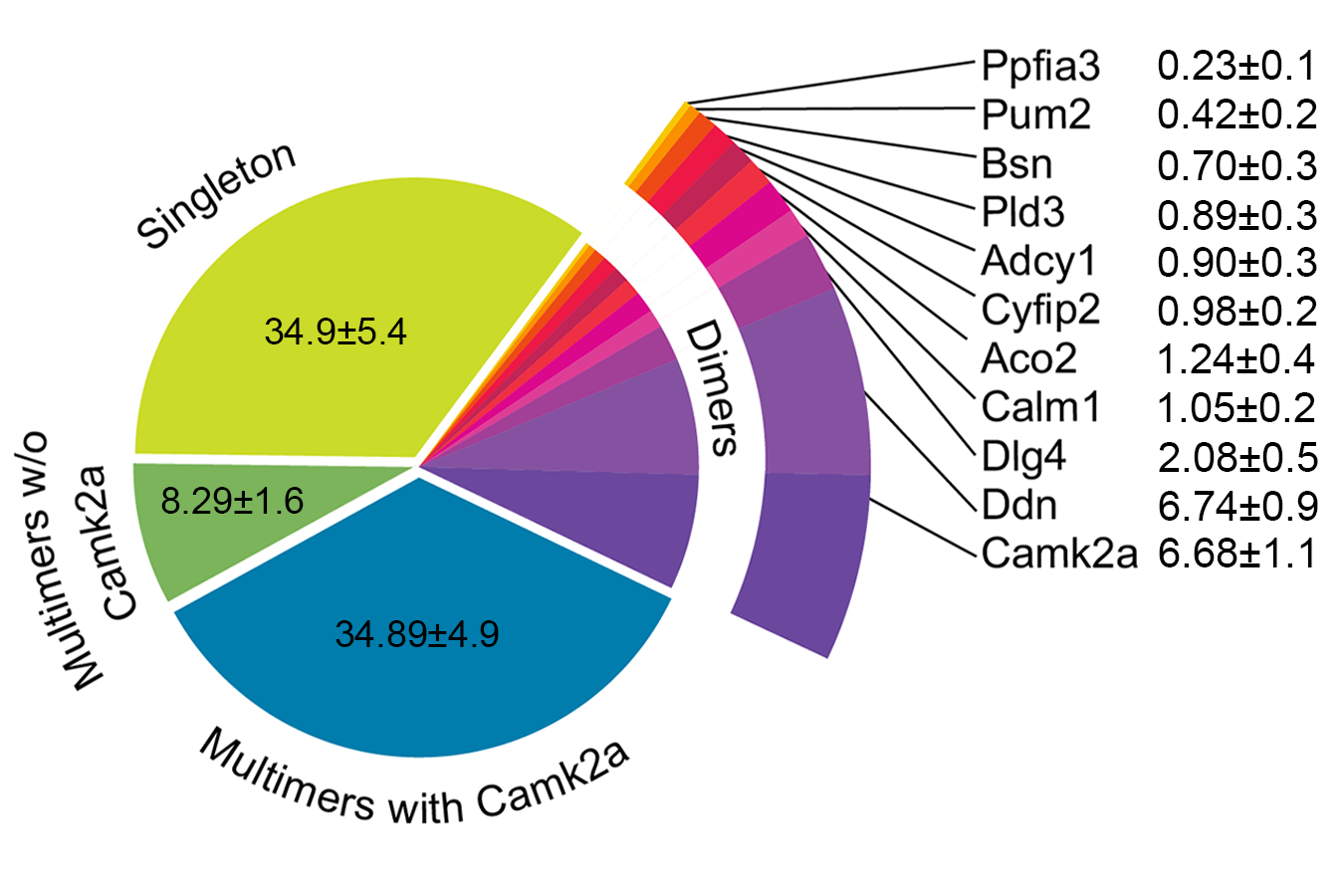

Supplement: Figure 4-4 — (Refers to Figure 4D) Pie chart of Psd mRNA composition that was observed due to random overlap of mRNA fluorescent puncta Psd image was rotated 90 degrees and colocalization of Psd with other eleven mRNAs combined were quantified and averaged from the same four 52X52 µm2 ROIs per animal as done for the registered experimental images. Individual animal averages were then averaged across N=4 mice and presented here as mean ± SEM. 65.1 ± 5.4% of Psd mRNA puncta (vs. 91.86 ± 1.8% in properly registered images) overlap randomly with at least one other mRNA that include dimers (Psd with only one other mRNA) or multimers (Psd with at least two other mRNAs). Consistent with the pairwise colocalization data where the extent of colocalization scales with mRNA abundance, the percentage of randomly colocalized dimers increases as mRNA abundance increases. However, the percentage of random dimers is equal to or greater than the percentage of dimers from properly registered images, with the exception of Psd/Camk2a dimers that are present at lower percentage than experimental (random Psd/Camk2a dimers 6.68 ± 1.1% versus properly registered Psd/Camk2a dimers 11.58 ± 3.1%). Random Psd-multimers with Camk2a (34.9 ± 5.0%) and without Camk2a (8.29 ± 1.6%) are appreciably lower than experimental images (57.91 ± 5.3% and 12.34 ± 2.9%, respectively), indicating multimer populations dominate colocalized Psd mRNA puncta compositions in our data. Download Figure 4-4, TIF file. [file eneuro-12-ENEURO.0184-25.2025-s009.tif]

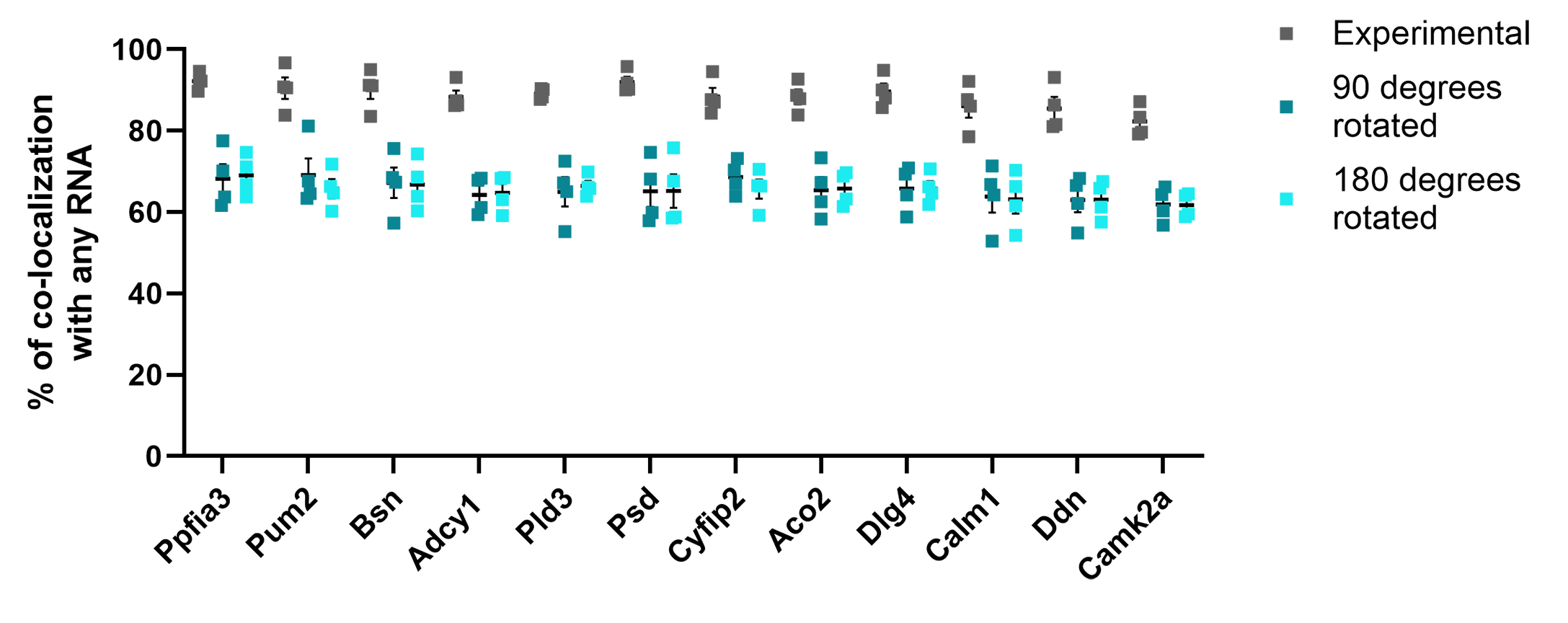

Supplement: Figure 4-5 — (Refers to Figure 4D) The majority of neuropil localized mRNAs spatially interact with at least one other mRNA Total % colocalization of each mRNA with any of the other 11 mRNAs from properly registered experimental images and 90 degree as well as 180-degree rotated images. Each symbol represents mean ± SEM from four biological replicates. % colocalization from experimental images were significantly higher compared to that in 90-degree rotated images (multiple unpaired two sample Welch’s t-test with FDR correction, p<0.01 for every pair) and 180-degree rotated images (multiple unpaired two sample Welch’s t-test with FDR correction, p<0.01 for every pair). N=4 mice. Error bars indicate SEM. Download Figure 4-5, TIF file. [file eneuro-12-ENEURO.0184-25.2025-s010.tif]

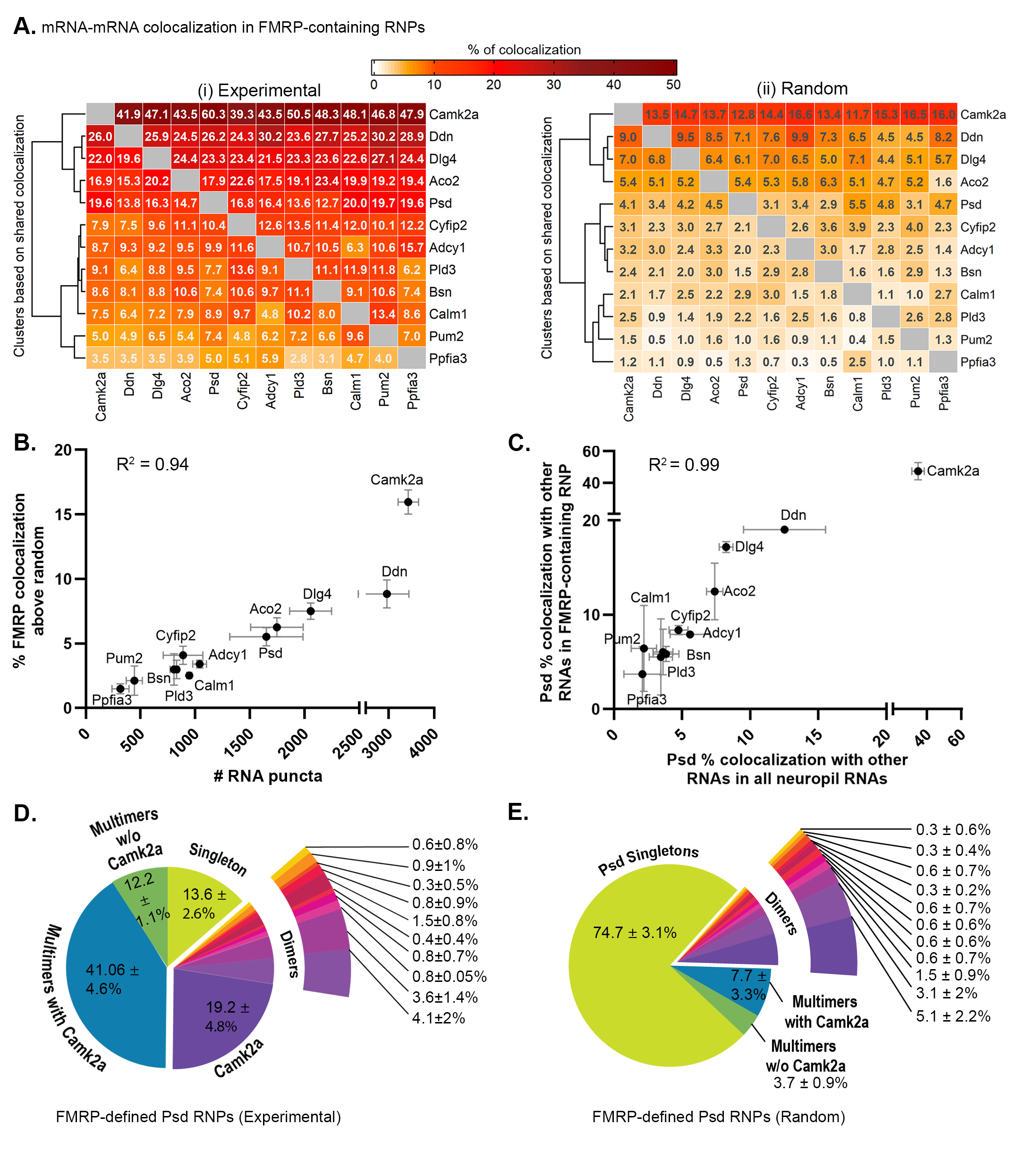

Supplement: Figure 4-6 — (Refers to Figure 4) Colocalization of mRNAs within FMRP-containing RNPs. A. Heatmaps of experimental (Ai. left) and random (Aii. right, 90 degree rotated) % colocalization of each mRNA pair within FMRP containing RNP. Percentage was calculated by dividing the number of overlapping puncta by the total number of the column mRNA puncta. B. Correlation plot of the % FMRP colocalized with each mRNA (random colocalization subtracted) and mRNA abundance (R2 = 0.94). C. Correlation plot of pairwise Psd colocalization percentage with other RNAs within all neuropil RNA puncta (X-axis) and FMRP-containing RNPs (Y-axis). D. FMRP-containing Psd RNP compositions in experimental images. E. FMRP-containing Psd RNP compositions in random 90-degree rotated images. (N=2 mice). Download Figure 4-6, TIF file. [file eneuro-12-ENEURO.0184-25.2025-s011.tif]

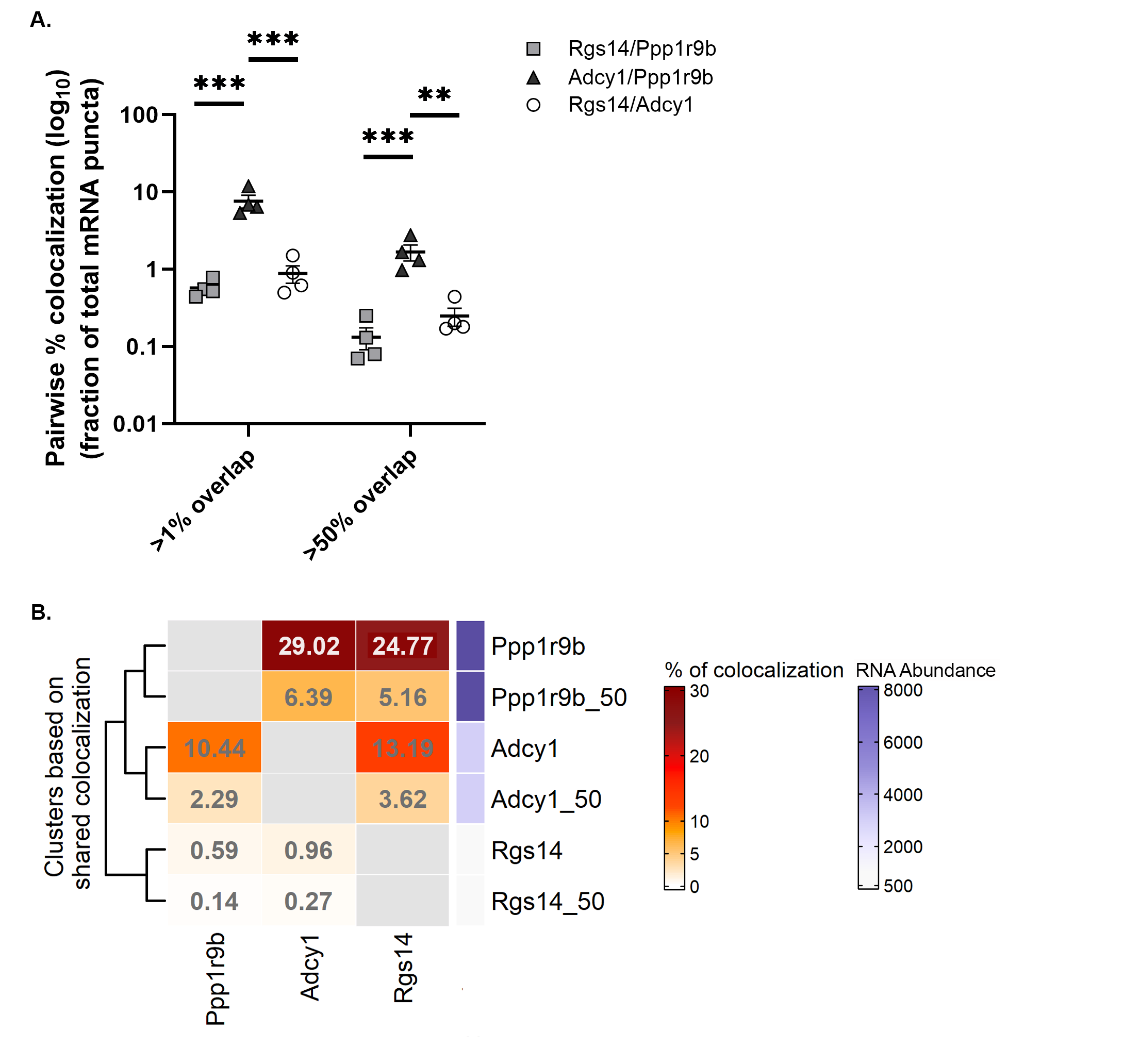

Supplement: Figure 5-1 — (Refers to Figure 5) 2D centroid-to-centroid distance based analysis reveals mRNA pairwise colocalization stratifies by abundance. A. 3Plex mRNA pairwise colocalization is expressed as a percentage of the combined total mRNA puncta count in DG. The two more abundant mRNAs, Adcy1 and Ppp1r9b, are colocalized (7.62 ± 1.45%) significantly more than Adcy1/Rgs14 (0.89 ± 0.22%) and Rgs14/Ppp1r9b (0.57 ± 0.07%) when colocalization is defined as >1% overlap (overall effect of mRNA pair, RM ANOVA: F = 55.86, p = 0.0001, N=4 mice) or >50% overlap (overall effect of mRNA pair, RM ANOVA, F = 28.80, p = 0.0008, N=4 mice). Stats were run on the transformed (log10) values as plotted to meet the normality assumption. Tukey’s post hoc tests reported on the plot. **p<0.01; ***p<0.001 B. Heatmap showing mRNA puncta colocalized at >1% and >50% overlap plotted as a percentage of each mRNA (averaged across N=4 mice +/- SEM, “_50” represents >50% overlap). Data hierarchically cluster by RNA pair and stratify by abundance (purple shading). Download Figure 5-1, TIF file. [file eneuro-12-ENEURO.0184-25.2025-s012.tif]

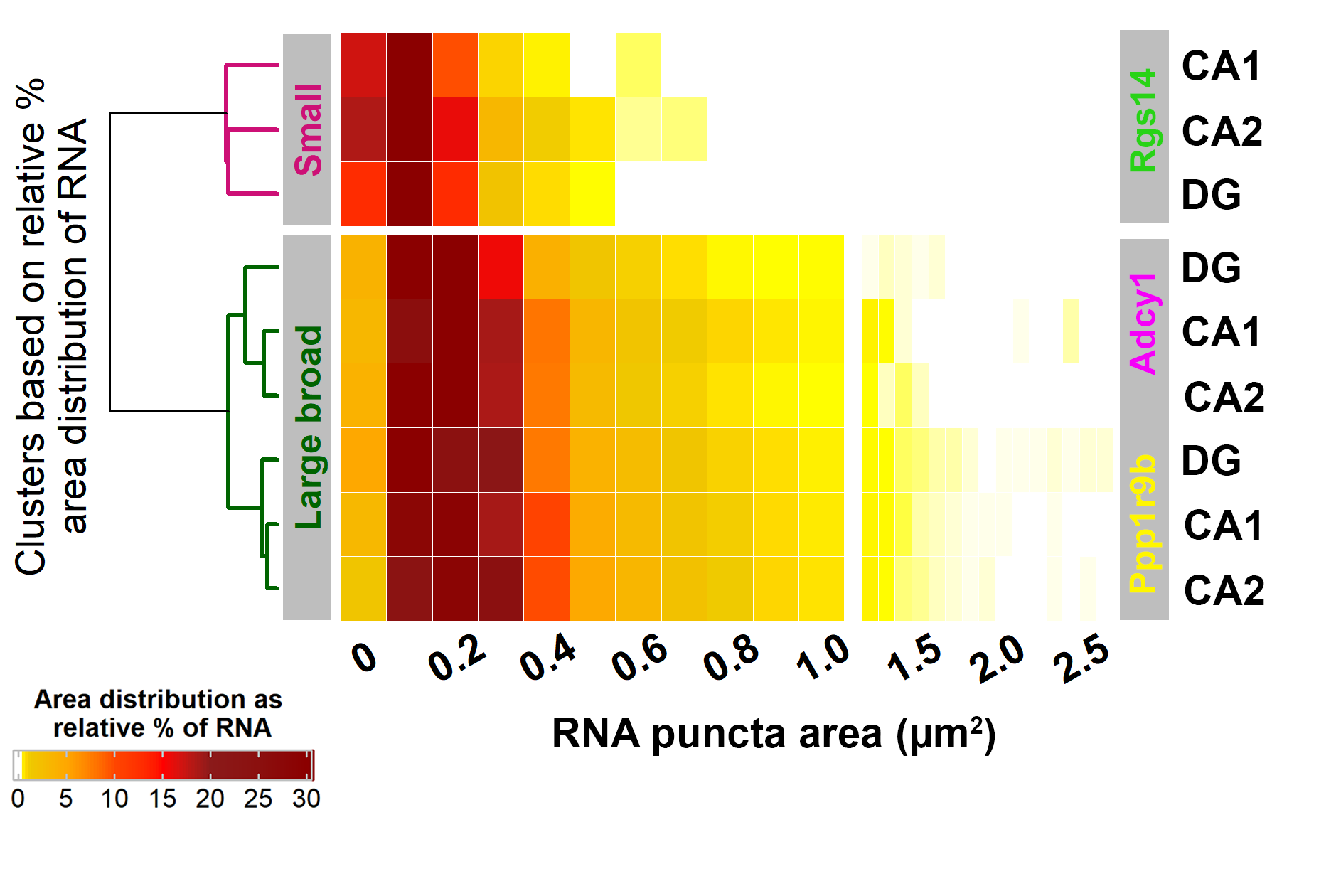

Supplement: Figure 5-2 — (Refers to Figure 5) Rgs14, Adcy1 and Ppp1r9b are variable in fluorescent puncta areas across mRNAs but not cell types. Hierarchical clustering of relative percent area distribution of Rgs14, Adcy1 and Ppp1r9b in CA2, CA1 and DG of adult mouse hippocampus. Median puncta areas were not significantly different across cell types for each mRNA although heterogeneity in area distribution across mRNAs was observed similar to the HiPlex data. Rgs14 median puncta area CA1: 0.10 ± 0.01 µm2, CA2: 0.10 ± 0.01 µm2, DG: 0.10 ± 0.02 µm2 (no effect of cell-type, RM ANOVA, F = 0.026, p = 0.9744, N=4 mice). Adcy1 median puncta area CA1: 0.22 ± 0.03 µm2, CA2: 0.22 ± 0.03 µm2, DG: 0.19 ± 0.02 µm2 (no effect of cell type, RM ANOVA: F = 0.5406, p = 0.6083, N=4 mice). Ppp1r9b median puncta area CA1: 0.22 ± 0.01 µm2, CA2: 0.24 ± 0.02 µm2, DG: 0.22 ± 0.03 µm2 (no-effect of cell type, RM ANOVA, F = 0.5507, p = 0.6032, N=4 mice). Since Adcy1 and Ppp1r9b median puncta area data were more likely to be a lognormal distribution, we repeated the RM ANOVA on log10 transformed values, which did not change the statistical result. Download Figure 5-2, TIF file. [file eneuro-12-ENEURO.0184-25.2025-s013.tif]
